# Muscleblind acts as a modifier of FUS toxicity by modulating stress granule dynamics and SMN localization

**DOI:** 10.1038/s41467-019-13383-z

**Published:** 2019-12-06

**Authors:** Ian Casci, Karthik Krishnamurthy, Sukhleen Kour, Vadreenath Tripathy, Nandini Ramesh, Eric N. Anderson, Lara Marrone, Rogan A. Grant, Stacie Oliver, Lauren Gochenaur, Krishani Patel, Jared Sterneckert, Amanda M. Gleixner, Christopher J. Donnelly, Marc-David Ruepp, Antonella M. Sini, Emanuela Zuccaro, Maria Pennuto, Piera Pasinelli, Udai Bhan Pandey

**Affiliations:** 1 Department of Human Genetics, University of Pittsburgh Graduate School of Public Health, Pittsburgh, PA USA; 2 Department of Pediatrics, Children’s Hospital of Pittsburgh, University of Pittsburgh Medical Center, Pittsburgh, PA USA; 3 Department of Neuroscience, Jefferson Weinberg ALS Center, Vickie and Jack Farber Institute for Neuroscience, Jefferson University, Philadelphia, PA USA; 4 Center for Regenerative Therapies TU Dresden, Technische Universität Dresden, Fetscherstr. 105, 01307 Dresden, Germany; 5 Department of Neurobiology, School of Medicine, University of Pittsburgh, Pittsburgh, PA USA; 6 Live Like Lou Center for ALS Research, Brain Institute, University of Pittsburgh School of Medicine, Pittsburgh, PA USA; 7 UK Dementia Research Institute at King’s College London, Institute of Psychiatry, Psychology and Neuroscience, Maurice Wohl Clinical Neuroscience Institute, King’s College London, London, SE5 9NU UK; 8 Department of Biomedical Sciences (DBS), University of Padova, Padova, Italy; 9 Veneto Institute of Molecular Medicine (VIMM), Padova, Italy

**Keywords:** Genetics, Neuroscience, Amyotrophic lateral sclerosis

## Abstract

Mutations in *fused in sarcoma (FUS)* lead to amyotrophic lateral sclerosis (ALS) with varying ages of onset, progression and severity. This suggests that unknown genetic factors contribute to disease pathogenesis. Here we show the identification of muscleblind as a novel modifier of FUS-mediated neurodegeneration in vivo. Muscleblind regulates cytoplasmic mislocalization of mutant FUS and subsequent accumulation in stress granules, dendritic morphology and toxicity in mammalian neuronal and human iPSC-derived neurons. Interestingly, genetic modulation of endogenous muscleblind was sufficient to restore survival motor neuron (SMN) protein localization in neurons expressing pathogenic mutations in FUS, suggesting a potential mode of suppression of FUS toxicity. Upregulation of SMN suppressed FUS toxicity in *Drosophila* and primary cortical neurons, indicating a link between FUS and SMN. Our data provide in vivo evidence that muscleblind is a dominant modifier of FUS-mediated neurodegeneration by regulating FUS-mediated ALS pathogenesis.

## Abstract (web summary)

The exact molecular mechanisms driving FUS-mediated toxicity remain unclear. Here, the authors demonstrate that muscleblind (Mbl) is a novel modifier of FUS-associated ALS, with knockdown of endogenous Mbl suppressing neuromuscular junction defects and motor dysfunctions associated with FUS expression in *Drosophila,* as well as restoring reduced SMN protein levels in mammalian neuronal and human iPSC-derived motor neurons.

## Introduction

Amyotrophic lateral sclerosis (ALS), also known as Lou Gehrig’s disease, is a devastating, neurodegenerative disorder that causes selective loss of upper and lower motor neurons, which eventually results in progressive paralysis and death owing to respiratory failure^1,2^. The average age of onset is between 50 and 80 years^3–7^, and the disease is usually fatal within 2–5 years following diagnosis^8–11^. Approximately 5–10% of ALS occurrences are inherited, most often in an autosomal dominant manner and are termed familial ALS (fALS)^12,13^, whereas the other 90–95% of cases are sporadic (sALS). However, the clinical and pathological symptoms of fALS and sALS patients are indistinguishable^14^. Mutations in the gene *FUS* (fused in sarcoma) account for ~ 4% of fALS and 1% of sALS cases^15^. FUS is a nuclear, DNA/RNA-binding protein that functions in several stages of RNA processing, including gene transcription^16^, alternative splicing^17,18^, and RNA trafficking^15,19–24^. Pathogenic mutations of *FUS* were first identified in ALS patients in 2009 and were found to cause mislocalization of the disease protein from the nucleus to the cytoplasm and accumulation into cytoplasmic aggregates, now commonly regarded to as stress granules (SGs)^25,26^. These changes mimicked those previously observed in ALS patients with mutations in another RNA-binding protein, TDP-43^20,27^. Interestingly, not only mutations of RNA-binding proteins, but also overexpression of the wild-type counterparts is sufficient to recapitulate pathogenetic pathways in cell and animal models^28–31^. These observations are further supported by the fact that upregulation of the endogenous wild-type proteins either by novel variants in untranslated region of FUS or triplication mutation (alpha synuclein) lead to neurodegenerative symptoms in human patients^32–37^. Cytoplasmic aggregates—specifically the ubiquitin-positive and tau-negative variety—have long been recognized as a pathological hallmark of ALS^38^.

To date, several ALS-linked proteins have been identified as components of cytoplasmic aggregates, which are believed to originate from aberrant SGs^24,39–48^. SGs are dynamic, ribonucleoprotein complexes that form membrane-less cytoplasmic structures in response to cellular stress such as heat, cold, infection, and oxidation. They sequester RNA and RNA-binding proteins to maintain tight control over mRNA processing so that cells can mount an appropriate response to the stress^42,49,50^. In FUS-associated ALS, several disease-causing mutations are located in the nuclear localization signal, potentially hindering its transport into the nucleus, thereby resulting in accumulation in the cytoplasm^39,51,52^. Because the N-terminal domain of FUS contains a prion-like domain, FUS is recruited into SGs, which may be a physiological, albeit uncontrolled and toxic, response^42,45,48,52,53^. ALS-causing mutations in RNA-binding proteins have been shown to perturb SG dynamics, leading to dysregulation of mRNA processing that may be directly related to the cellular toxicity observed in ALS^42,43^.

However, the exact molecular mechanisms driving pathology are still poorly understood. ALS is a heterogeneous disease condition where the age of onset and disease progression varies significantly between individuals who share a single-point mutation in an ALS-causing gene^54^. This is true for both sALS patients and fALS cases where all affected family members have the same point mutation^55^. This suggests that other unknown factors, whether intrinsic or extrinsic, must be contributing to disease pathogenesis. Identification of genetic modifiers of ALS-associated FUS toxicity could help in understanding the molecular mechanisms underlying motor neuron degeneration. We performed an unbiased genetic screen to identify dominant modifiers of neurodegenerative phenotypes in vivo. Here, we show that muscleblind (Mbl), encoded by the *mbl* gene, is a novel modifier of FUS-associated ALS in fruit flies (*Drosophila melanogaster*). Specifically, knocking down endogenous Mbl via RNAi-suppressed eye degeneration caused by ectopic expression of mutant human FUS protein, strongly suppressed neuromuscular junction (NMJ) defects and ameliorated adult locomotor abilities. Importantly, ectopic expression of muscleblind enhanced FUS toxicity in vivo. Interestingly, Mbl RNAi did not suppress toxicity associated with TDP-43, expanded C9orf72 repeats and VCP, suggesting specificity for FUS-mediated neurodegeneration. Knockdown of endogenous mbnl1 suppressed FUS-associated cellular toxicity and defects in dendrite morphology in primary neurons. In a mammalian cell line, knockdown of human muscleblind-like protein 1 (MBNL1) significantly reduced the cytoplasmic mislocalization of mutant FUS and its subsequent incorporation into SGs. We also observed that shRNA-mediated knockdown of MBNL1 strongly reduced FUS-positive SGs in iPSC neurons expressing FUS-P525L mutant. Furthermore, knockdown of endogenous MBNL1 in primary cortical neurons expressing FUS restored localization of survival motor neuron (SMN) protein, establishing a link to another pathway associated with motor neuron pathology. Our data provide further insight into the mechanisms behind FUS toxicity and identified muscleblind as a novel modifier of FUS-ALS that may be useful in identifying potential therapeutic targets for ALS.

## Results

### An unbiased genome-wide screen identifies muscleblind (Mbl) as a novel modifier of FUS toxicity in vivo

**Fig. 1:**
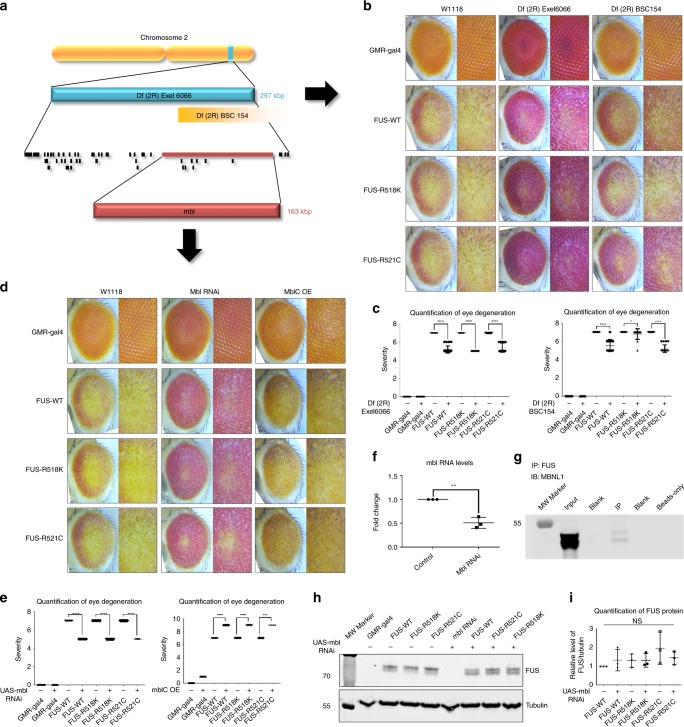
Muscleblind (Mbl) is a novel modifier of FUS toxicity in vivo. An unbiased genetic screen using our *Drosophila* model of ALS revealed candidate modifiers of toxicity caused by expression FUS. **a** Schematic of the Df(2 R)Exel6066 line identified in the screen and the relative locations of the genes within the deficient region. The Df(2 R)BSC154 deficiency region overlapped with *mbl*, beginning in the middle of the gene. **b** Representative panel of adult *Drosophila* eyes showing degeneration caused by expression of wild-type and ALS-linked mutant FUS with and without concurrent heterozygous depletion of the genes within the indicated deficiency lines. **c** Quantification of eye degeneration severity in *Drosophila* indicating significant suppression of FUS-associated toxicity when these flies are crossed with the deficiency lines (****P < 0.0001, *P = 0.0160). **d** Representative images of *Drosophila* eyes expressing the indicated FUS proteins alone (left column), in combination with RNAi-mediated knockdown of *mbl* (middle column), or with overexpression of Mbl isoform C (right column). (*N* = 17–117). **e** Quantification of eye degeneration severity confirms significant suppression of FUS toxicity following depletion of *mbl* (left graph), and significant enhancement of toxicity following overexpression of MblC (right graph) (****P = 0.0001, ***P = 0.0003). **f** qPCR of RNA (*n* = 3) confirms significant knockdown of endogenous *mbl* in the RNAi line (**P = 0.0021). **g** Western blot (WB) analysis following co-immunoprecipitation (Co-IP) experiments using HEK293T cells. Co-IP was performed using anti-FUS antibody to target endogenous FUS (*n* = 3). WBs were probed for human MBNL1, which was present in the input control and the immunoprecipitated samples but absent from the negative control containing only beads. **h** WB showing FUS and tubulin protein levels in *Drosophila* expressing wild-type and mutant FUS (*N* = 3). **i** Quantification of FUS normalized to tubulin, indicating equivalent FUS expression in all FUS-expressing groups. In addition, knockdown of Mbl has no effect on FUS levels (NS = not significant, *P* = 0.3578). Statistical significances in **c** and **e** were determined using two-tailed *t* tests for each FUS pair (Mann–Whitney test). One-tailed *t* test was used in **f**. One-way ANOVA with Tukey’s multiple comparisons test was used for **h**. All quantifications are represented as the mean ± SD.

We performed an unbiased genome-wide screen to identify novel modifiers (enhancers and suppressors) of FUS toxicity in *Drosophila* eyes (Supplementary Fig. 1a, Supplementary Table 1–3). We used FUS R521H transgenic fly line that shows moderate external eye degenerative phenotype for doing our genetic screen. As the *Drosophila* genome has been fully sequenced, the modifying deficiency lines provided a set of candidate genes within the deleted regions potentially responsible for modifying mutant FUS toxicity. We identified two overlapping deficiency lines that strongly suppressed FUS-mediated degeneration, Df(2 R)Exel6066 and Df(2 R)BSC154 (Fig. 1a). To validate these findings, we crossed both deficiency lines with *Drosophila* lines expressing either wild-type FUS or two additional disease-causing mutations FUS-R518K and FUS-R521C (Fig. 1b, c). Both deficiency lines significantly suppressed wild-type and mutant FUS-induced degeneration of *Drosophila* eyes. The deleted region of the Df(2 R)Exel6066 deficiency line contains 44 known and predicted genes (Supplementary Table 3). To identify the gene(s) within this region responsible for modifying FUS toxicity in vivo, we obtained all the available RNAi lines targeting genes mapping within this region. Using two independent RNAi lines, we found that knockdown of *Drosophila* muscleblind (*mbl*), which overlaps both Df(2 R)Exel6066 and Df(2 R)BSC154 deficiency regions, was sufficient to suppress wild-type and FUS-R521H toxicity (Supplementary Fig. 1b and Supplementary Fig. 2a, b). However, knockdown of other genes within the Df(2 R)Exel6066 and *CG12699* deficiency regions did not suppress FUS toxicity (Supplementary Fig. 3). To further validate whether Mbl is a novel modifier of FUS-induced toxicity in vivo, in addition to the loss of function approach, we also undertook a gain of function approach by generating flies that overexpress fly Mbl that showed an enhancement in the FUS toxicity

The human muscleblind-like (MBNL) protein has been previously linked to myotonic dystrophy^56^, fragile X syndrome^57^, spinocerebellar ataxia^58^, and Huntington’s disease^59,60^. In addition, *mbl* RNA contains long intronic regions. As FUS preferentially binds to long intronic regions and has been shown to bind RNA from human orthologs of *mbl* (*MBNL1*, *MBNL2*, and *MBNL3*)^18,61^, *mbl* was further analyzed as a potential modifier of FUS-associated ALS. FUS lines with known site-specific integration (SSI) of the transgenes were used to further validate whether reducing *mbl* RNA levels would suppress FUS toxicity. Wild-type FUS and the two ALS-linked mutant FUS-R518K and FUS-R521C lines with known SSI of the transgenes were crossed with *mbl* RNAi lines, which were also generated using site-specific integration methods. RNAi-mediated knockdown decreased *mbl* RNA by ~ 49% and significantly suppressed external eye degeneration in all FUS-expressing *Drosophila* (Fig. 1c–f). Knockdown of endogenous Mbl did not reduce FUS protein levels in the *Drosophila*, indicating that this effect was not due to a loss of toxic protein (Fig. 1h, i). Knocking down endogenous Mbl alone in these flies was well-tolerated, as it did not cause any obvious external eye degeneration itself (Fig. 1d). Importantly, ectopic expression of fly Mbl in these FUS lines significantly enhanced external eye degeneration, further supporting the idea that Mbl is a modifier of FUS toxicity (Fig. 1d, e). Cross-sectional imaging of the eyes revealed that FUS-mediated loss of tisues beneath the surface were rescued by knocking down Mbl r (Supplementary Fig. 2c). Wild-type and mutant FUS expression caused retinal degradation, length reduction, and separation from the lamina at the basal membrane. Knockdown of Mbl in these flies attenuated these defects, whereas Mbl overexpression had the opposite effect, suggesting Mbl specifically regulates FUS toxicity in vivo. As degenerative effects associated with FUS toxicity in *Drosophila* are known to increase in an age-dependent manner^62^, we tested whether knockdown of Mbl suppresses FUS-induced eye degeneration over time. We observed that Mbl knockdown suppressed FUS-mediated degeneration in aged animals as well (Supplementary Fig. 4).

Based on these results, we sought to further validate the link between FUS and Mbl. To test whether endogenous human FUS and MBNL1 physically interact, we performed co-immunoprecipitation (IP) using human embryonic kidney cells (HEK293T) and found that endogenous FUS and MBNL1 physically interact in these cells (Fig. 1g). We asked if FUS and MBNL1 interaction is RNA-dependent in mammalian cells. We transfected HEK293T cells with HA-tagged FUS (WT and mutant), treated cells with RNase and performed immunostaining of cells with anti-HA and anti-MBNL1 antibodies. We did not see any difference in co-localization pattern of FUS and MBNL1, suggesting that their interaction is not RNA-dependent (Supplementary Fig. 5a). In parallel, we performed IP with anti-HA followed by RNase digestion and western blot (WB) with anti-MBNL1. We found that FUS and MBNL1 interaction is not dependent on RNA (Supplementary figure 5b). FUS localization and aggregation is dependent on its association with transportin 1, phase separation, and posttranslational arginine methylation of FUS^63–67^. We have previously shown that genetic modulation of arginine methyltransferases is sufficient to modify FUS toxicity in vivo^68^. To examine if MBNL1 affects the posttranslational modification (arginine methylation) of FUS, we decided to express FUS (WT and mutant) with green fluorescent protein (GFP)-MBNL1 in HEK293T cells and treated the cells with AdOx (a pan-inhibitor of protein arginine methylation). We performed WB to examine if FUS (lower band) is modulated through arginine modification. We found that neither lower nor upper band intensities are changed with or without MBNL1 RNAi KD in the presence or absence of AdOx, suggesting that MBNL1-mediated suppression of FUS toxicity is likely to be independent of arginine methylation (Supplementary Fig. 5c). Next, we examined the nuclear and cytoplasmic distribution of FUS in response to AdOx treatment, but we did not observe any difference in the FUS distribution with and without AdOx treatment in any groups (Supplementary fig. 5d). These observations suggest that MBNL1-mediated suppression of FUS toxicity does not appear to be dependent of arginine methylation modification.

We next sought to determine whether pathogenic mutations of FUS influence endogenous Mbl expression in *Drosophila* and ALS patient cells. Only *Drosophila* expressing mutant FUS-R518K exhibited a mild increase in *mbl* RNA compared with the controls. However, this increase was not observed in *Drosophila* expressing wild-type FUS or FUS R521C (Supplementary Fig. 6a). Furthermore, no differences in endogenous MBNL1 RNA expression were observed between ALS patient lymphoblastoid cells expressing ALS-causing FUS mutants compared to cells from controls (Supplementary Fig. 6b). In addition, knockdown of endogenous cabeza, the *Drosophila* orthologue of human FUS encoded by the *caz* gene, did not alter Mbl expression in *Drosophila* (Supplementary Fig. 6c). Measurement of Mbl protein is difficult in *Drosophila* owing to a lack of commercially available antibodies. Therefore, these results were further validated by measuring endogenous MBNL1 protein levels in ALS patient lymphoblastoid cells; no differences were observed compared with control cells (Supplementary Fig. 6d).

To rule out whether suppression of FUS toxicity is owing to dilution of gal4 from the additional upstream activating sequence (UAS)-*mbl* RNAi transgenic element in the crosses, *Drosophila* expressing wild-type or mutant FUS were crossed with three different control lines expressing the GFP and luciferase. Expression of either GFP or luciferase did not modify eye degeneration (Supplementary Fig. 7). These results further suggest that depleting endogenous mbl in *Drosophila* suppresses FUS toxicity without changing FUS levels.

To determine whether knockdown of Mbl suppresses degeneration caused by other ALS-linked proteins or was specific to FUS-associated ALS, *mbl* RNAi *Drosophila* were crossed with lines expressing wild-type or ALS-linked mutant forms of several proteins (EWSR1, TDP-43, dVCP, and C9orf72) (Supplementary Fig. 8). Mild suppression of EWSR1-dependent degeneration was observed with Mbl knockdown; however, Mbl knockdown did not suppress the toxicity of other proteins known to cause ALS. Therefore, the rescue observed appears to be specific for FUS-associated ALS and could likely be extended to the FET family of proteins (FUS, EWSR1, and TAF15).

### Mbl knockdown suppresses FUS-associated neuronal defects in vivo

**Fig. 2:**
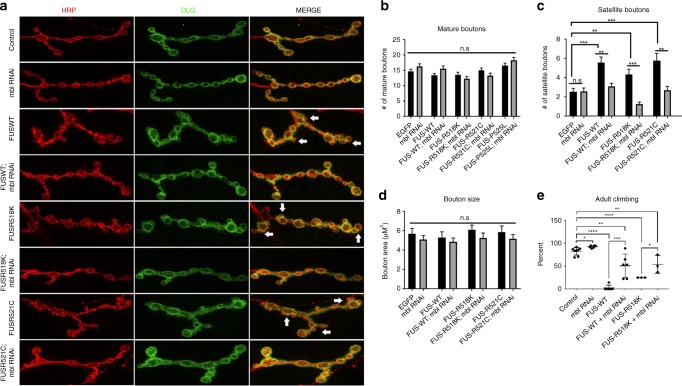
Mbl RNAi suppresses neuromuscular junction defects and motor dysfunction. Neuronal expression of wild-type and ALS-linked mutants of FUS cause defects in larval neuromuscular junction (NMJ) morphology and motor dysfunction, both of which are rescued by knockdown of endogenous Mbl. Tissue-specific expression of FUS in the motor neurons (D42-gal4) of third-instar larvae causes morphological defects of NMJs at muscle 4 from segments A2–A5. **a** Immunofluorescence images of NMJs using presynaptic (HRP) and postsynaptic (DLG) markers. Scale bars = 10 µm. The average number **b** and size of mature boutons **d** are unaffected by FUS expression. **c** Expression of FUS in motor neurons results in an aberrant increase satellite bouton numbers (white arrows), which is significantly rescued by knockdown of endogenous Mbl. (*N* > 25 NMJs from > 8 animals were used for analyses). Values in **b** and **c** are represented as the mean ± SEM. Statistical comparisons in **b**–**d** were determined using one-way ANOVA with Tukey’s multiple comparisons test for each FUS group (*P < 0.05, **P < 0.01, ****P < 0.0001, NS = not significant). Comparisons between control and *mbl* RNAi groups were performed with unbalanced *t* tests. **e** Quantification of the percent of adult flies that climb up to 10 centimeters. Values are represented as the mean ± SD. (*N* > 50 flies with data were collected in triplicate). Statistical comparisons were performed on GraphPad Prism using one-way ANOVA with Tukey’s multiple comparisons test for each FUS group.

As ALS primarily affects upper and lower motor neurons, we sought to determine whether knockdown of Mbl would mitigates FUS-associated toxicity in *Drosophila* motor neurons (D42-gal4 driver). Wild-type FUS and FUS-R518K were expressed in fly motor neurons along with *mbl* RNAi. The NMJs of progeny at the third-instar larval stage were analyzed by confocal microscopy (Fig. 2a). We observed a significant increase in the number of satellite boutons in larvae expressing FUS wild-type, FUS R518K, and FUS R521C compared with controls. No significant differences were observed in the number or size of parent boutons in FUS-expressing animals or Mbl RNAi KD alone (Fig. 2b, c). Knockdown of Mbl significantly reduced the number of satellite boutons in both WT and mutant FUS-expressing larvae. To determine the physiological significance of these morphological defects, we decided to measure the motor function of FUS-expressing animals by measuring their climbing ability. Expression of either wild-type or mutant FUS significantly reduced the percentage of flies able to climb compared with control flies (Fig. 2e). We found that knockdown of endogenous Mbl significantly increased the percentage of climbing flies, thereby rescuing motor dysfunction. Collectively, these results indicate that depletion of endogenous Mbl rescues morphological defects at NMJs as well as motor dysfunction. Based on these findings, we propose Mbl as a novel modifier of ALS-associated FUS toxicity in neurons.

### Knocking down endogenous MBNL1 reduces mutant FUS incorporation into SGs

Cytoplasmic mislocalization of mutant FUS and its subsequent accumulation into cytoplasmic SGs are a pathological hallmark of ALS^25,26,41^. These events have been linked with mutant FUS protein toxicity and motor neuron death^24,69,70^. Therefore, we hypothesized that decreasing muscleblind protein levels in mammalian cells would reduce FUS incorporation into SGs, thereby suppressing toxicity and cell death. To test this, we used HEK293T cells for investigating changes in FUS distribution following knockdown (KD) of endogenous muscleblind proteins. Although only one *mbl* gene exists in *Drosophila*, humans and rats express three muscleblind paralogues, called muscleblind-like 1, 2, and 3 (*MBNL1*, *MBNL2*, and *MBNL3*). In addition, expression of the three paralogues is variable in human tissues. MBNL1 and MBNL2 are the most ubiquitously expressed, including fetal and adult brain, heart, kidney, liver, lung, and skeletal muscle. MBNL3 is not as widely expressed and is found primarily in the heart, liver, pancreas, and placenta^71^. As MBNL1 is the paralogue most frequently linked to other diseases, including neurodegenerative diseases^56–60^, and is well conserved with *Drosophila mbl*, we chose to focus on human MBNL1 for our studies in mammalian systems^72^.

To evaluate the effect of knocking down MBNL1 on wild-type and mutant FUS localization and SG development in mammalian cells, we measured the effect of FUS expression on the distribution of endogenous MBNL1 in HEK293T cells. Untransfected HEK293T cells treated with different external stressors formed MBNL1-positive SGs (Supplementary Fig. 9a). Therefore, we assessed the subcellular distributions of wild-type FUS, as well as FUS-R518K and FUS-R521C in transiently transfected HEK293T cells treated with sodium arsenite to induce SG formation (Supplementary Fig. 9b and 10). By confocal microscopy, we found that in the absence of stress, wild-type FUS primarily localized to the nucleus and was not incorporated into MBNL1-positive SGs. In contrast, FUS-R518K and FUS-R521C mislocalized to the cytoplasm and incorporated into MBNL1-positive SGs (Supplementary Fig. 10). The presence of SGs in the untreated cells suggests that ALS-associated mutations of FUS are sufficient for SG formation, even in the absence of additional cellular stress, as we have previously observed (Scaramuzzino et al., 2013 *Plos ONE*).

**Fig. 3:**
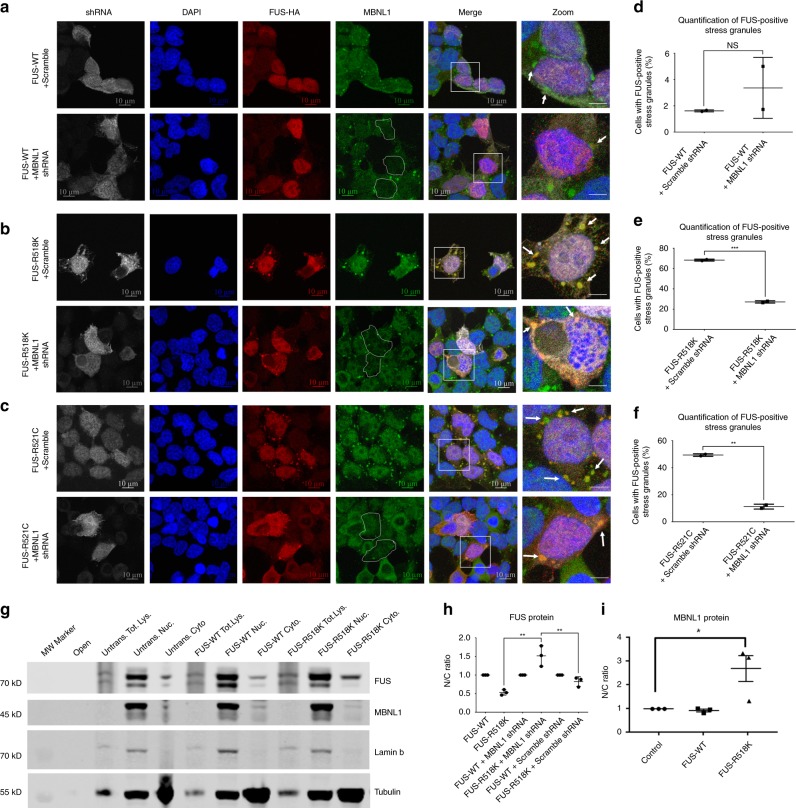
MBNL1 KD reduces mutant FUS mislocalization and incorporation into SGs. HEK293T cells were co-transfected with the indicated FUS constructs (wild-type, R518K, or R521C) and either *MBNL1* or scrambled shRNA and treated with sodium arsenite. Cellular distributions of FUS, MBNL1, and the presence of SGs were assessed by confocal microscopy. **a**–**c** Representative confocal images showing FUS and MBNL1 distributions in HEK293T cells. White outlines in the MBNL1 column indicate representative *MBNL1* shRNA-transfected cells with reduced MBNL1 signal. White boxes indicate the areas shown in the last column. White arrows indicate representative stress granules. Scale bars = 10 µm in columns 1–5; scale bars = 5 µm in the last column. **d**–**f** Quantification of the percentage of cells with FUS-positive SGs confirms that depletion of endogenous MBNL1 significantly reduces mutant FUS integration into SGs (one graph per FUS group; *N* > 20 cells). Statistical analyses were determined using two-tailed *t* tests (***P = 0.0006, **P = 0.0014, NS = not significant). **g** Representative WB probed for FUS, MBNL1, Lamin B, and tubulin proteins. Unstressed HEK293T cells were transfected with FUS alone (either wild-type or mutant). Cytoplasmic and nuclear fractions of transfected cells were isolated, and levels of the indicated proteins were assessed. Lamin B and tubulin were used as loading controls for the nuclear and cytoplasmic fractions, respectively. (Tot. Lys. = Total Lysates, Nuc. = Nuclear Lysates, Cyto. = Cytoplasmic Lysates). **h** Nuclear/cytoplasmic ratios (N/C ratios) of FUS constructs. N/C ratios for FUS mutants were normalized to the ratios for wild-type FUS to compare between WBs. FUS protein levels for each fraction were normalized to lamin B for the nuclear fractions and tubulin for the cytoplasmic fractions. Nuclear values were divided by cytoplasmic values to obtain the N/C ratios. No statistically significant differences were observed between cells expressing mutant FUS alone and cells co-transfected with scrambled shRNA. **i** N/C ratios of MBNL1 in HEK293T cells expressing FUS. One-way ANOVA was performed to determine statistical significance in **h** using Tukey’s multiple comparisons test and in i using Dunnett’s multiple comparisons test (**P = 0.0015, NS = not significant).

We next determined the effect of MBNL1 knockdown in HEK293T cells by transiently transfecting short-hairpin RNAs (shRNA) that specifically targeted all isoforms of MBNL1. After validating successful knockdown of endogenous MBNL1 (Supplementary Fig. 11a–d), cells were co-transfected with either wild-type or mutant FUS-R518K and FUS-R521C (Fig. 3 and Supplementary Fig. 12). Treatment with either MBNL1 shRNA or scramble shRNA did not affect the nuclear localization of WT FUS (Supplementary Fig. 12a). Upon treatment with sodium arsenite the cells formed MBNL1-positive SGs that did not contain wild-type FUS (Fig. 3a, d). In both FUS R518K and FUS R521C-expressing cells, mutant FUS expression was sufficient to induce SG formation (Supplementary Fig. 12b, c). When we depleted endogenous MBNL1, the percentage of cells with FUS-positive SGs was significantly reduced (Supplementary Fig. 12d–11f), and this effect became more obvious upon sodium arsenite treatment (Fig. 3b, c, e, f). Though sodium arsenite induced SG formation in all three groups, the predominantly nuclear localization of wild-type FUS prevented its incorporation into cytoplasmic SGs. These observations support our hypothesis that MBNL1 depletion specifically reduces mutant FUS-positive SGs in the cytoplasm. The reduction of cytoplasmic mislocalization of FUS further supports our hypothesis that MBNL1 as a modifier of mutant FUS toxicity.

To assess whether these SG changes are owing to altered nuclear and cytoplasmic localization of FUS, we measured FUS levels in cellular fractions of unstressed HEK293T cells, with and without MBNL1 knockdown. When normalized to wild-type FUS levels, which exhibited a predominant nuclear localization pattern, the nuclear/cytoplasmic ratio (N/C ratio) of FUS-R518K was significantly increased upon MBNL1 knockdown (Fig. 3g, h, Supplementary Fig. 11e). Thus, depletion of endogenous MBNL1 partially restored the nuclear fraction of FUS compared with cells expressing FUS alone or co-expressing scramble shRNA. A significant change was observed in the N/C ratio of MBNL1 in FUS-R518K expressing cells compared with controls (Fig. 3i), which is consistent with our data in FUS-R518K flies showing a slight elevation in the FUS RNA levels (Supplementary Fig. 6a). These data suggest that the reduction of FUS-positive SGs in the cytoplasm following knockdown of endogenous MBNL1 is due to reduced incorporation of mutant FUS into the SGs.

### MBNL1 KD prevents mutant FUS mislocalization and incorporation into SGs

**Fig. 4:**
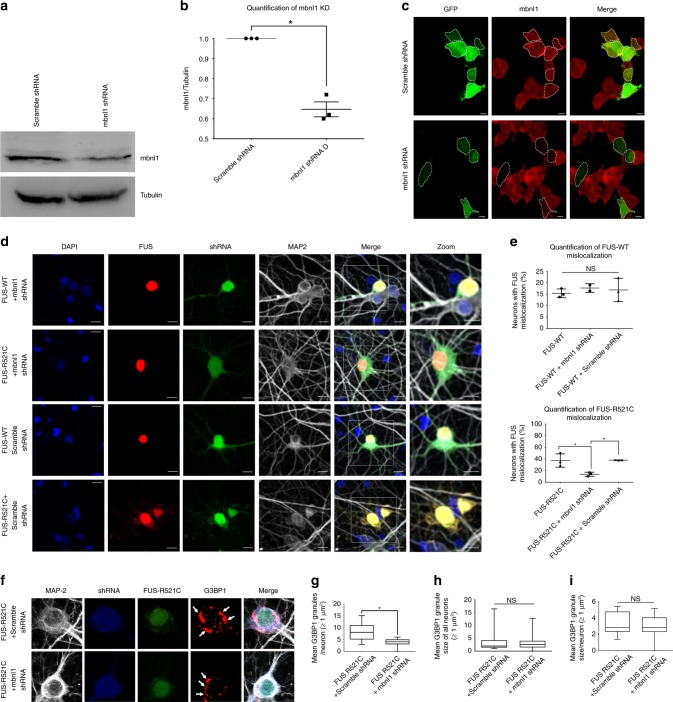
Mbnl1 KD rescues mutant FUS mislocalization in primary cortical neurons. Rat primary cortical neurons (PC12 cells) were co-transfected with FUS (either wild-type or R521C) and either M*bnl1*-specific or scrambled shRNA. FUS-positive cells were analyzed for cellular distribution of exogenous FUS and SG formation. **a**–**c** Validation of mbnl1 knockdown in PC12 cells. **a** Representative WB of PC12 cells transiently transfected with either scramble or *mbnl1*-specific shRNA. **b** Quantification of mbnl1 knockdown in PC12 cells (*N* = 3). **c** mbnl1 immunofluorescence (red) in scrambled and *mbnl1* shRNA-transfected PC12 cells (green). **d** Representative confocal images showing differences in wild-type and mutant FUS distributions in cortical neurons. Similar to cells transfected with FUS alone (Supplementary Fig. 13c), cells expressing scrambled shRNA showed wild-type and ALS-linked mutant FUS distributed in the nucleus and cytoplasm, respectively. However, in cells expressing *mbnl1*-specific shRNA, mutant FUS localizes primarily in the nucleus. White boxes indicate the magnified areas shown in the final column that highlight mislocalization of mutant FUS in scrambled shRNA-treated cells. **e** Quantification of the percentage of neurons with FUS mislocalization in each group, including the controls shown in Supplementary Fig. 13c. The results indicate significant rescue of mutant FUS-R521C mislocalization in cells co-transfected with *mbnl1* shRNA compared with the scramble shRNA control group (*N* > 30 cells/group). **f** Representative fluorescent images of sodium arsenite induced SGs in cortical neurons expressing mutant FUS and either *mbnl1* or scrambled shRNA. MAP2 and G3BP1 are markers of neurons and SGs, respectively. Cells expressing *mbnl1* shRNA show fewer cytoplasmic SGs compared with the scrambled shRNA group. **g**–**i** Quantification of the number and size of SGs in each group. Only SGs ≥ 1 µm^2^ were used. *N* > 15 cells/group. **g** The average number of G3BP1-positive SGs/neuron is significantly lower in *mbnl1* shRNA-expressing cells than in scrambled shRNA control cells. No differences in the average size of FUS-positive SGs occur between shRNA groups **h** across all transfected neurons or **i** averaged per neuron. One-way ANOVA with Tukey’s multiple comparisons test was used in **e** (*P = 0.008, NS = not significant). Two-tailed *t* tests were used for comparisons in **g**–**i** (*P = 0.0028, NS).

To test whether muscleblind modifies FUS toxicity in mammalian neurons, we used rat primary cortical neurons as a model for FUS-associated toxicity^30,73–77^. As with humans, rats have three paralogues of muscleblind, muscleblind-like 1, 2, and 3 (*Mbnl1*, M*bnl2*, and M*bnl3*)^71^. We used rat PC12 and mouse neuro2a (N2A) cells to identify and validate an M*bnl1*-specific shRNA that significantly deplete endogenous MBNL1 levels (Fig. 4a–c, Supplementary Fig. 11c, d. Furthermore, successful knockdown of endogenous Mbn1 in rat primary cortical neurons was validated by fluorescence microscopy (Supplementary Fig. 13a, b). Similar to endogenous MBNL1 in HEK293T cells, MBNL1 localized to G3BP stress granule assembly factor 1 (G3BP1)-positive SGs in primary cortical neurons when stressed (Supplementary Fig. 9c).

Using this cortical neuron model, we tested whether depleting endogenous MBNL1 would reduce cytoplasmic mislocalization of mutant FUS and its subsequent incorporation into SGs. Cortical neurons were co-transfected with either wild-type or the ALS-linked FUS-R521C mutant and MBNL1-specific shRNA or scrambled shRNA. FUS distribution was visualized by fluorescence microscopy with and without knockdown of endogenous mbnl1 (Fig. 4). In cells transfected with FUS alone, wild-type FUS primarily localized to the nucleus, whereas FUS-R521C mislocalized to the cytoplasm (Supplementary Fig. 13c). These localization patterns also occurred in cells co-transfected with scrambled shRNA, indicating no significant change in the percentage of neurons with mislocalized FUS (Fig. 4d, e). However, shRNA-mediated knockdown of endogenous MBNL1 significantly reduced mutant FUS mislocalization, with no effect on wild-type FUS distribution (Fig. 4d, e). An antibody specific for G3BP1 was used to analyze SG formation in cells expressing FUS-R521C treated with sodium arsenite (Fig. 4f). The average number of G3BP1-positive SGs per neuron was significantly reduced in Mbnl1 shRNA-expressing cells compared with scramble controls (Fig. 4g). However, no significant changes were observed in the average size of FUS-positive SGs across all neurons compared with scramble controls (Fig. 4h, i). This rescue appears to be specific for mutant FUS, as no changes in number or size of SGs in *Mbnl1* shRNA-expressing neurons were observed in the absence of exogenous FUS compared with scramble controls (Supplementary Fig. 14). Reduction of endogenous MBNL1 appeared to specifically affect the number, but not the size, of FUS R521C-positive SGs. The decrease in cytoplasmic mislocalization of FUS in cortical neurons expressing *mbnl1* shRNA further supports this hypothesis (Fig. 4e). These results provide evidence that MBNL1 modifies FUS toxicity by altering its cellular distribution and interaction with SGs in the cytoplasm.

### MBNL1 KD suppresses FUS-induced toxicity and morphological defects

**Fig. 5:**
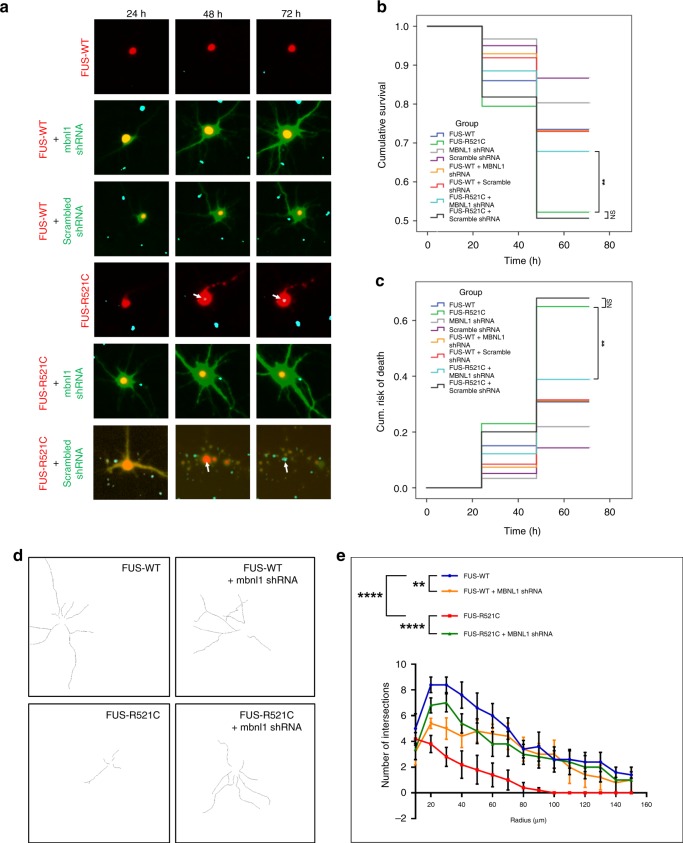
MBNL1 KD suppresses mutant FUS toxicity and morphological defects. Cortical neurons were co-transfected with either FUS-WT or FUS-R521C and either *Mbnl1* or scrambled shRNA and assessed for cell death and morphological changes. **a** Representative live imaging, fluorescence microscopy images of neurons at 24, 48, and 72 h post transfection with FUS and shRNAs. Cell death is indicated by positive staining with DRAQ7 (cyan spots indicated with white arrows). DRAQ7 is not present in cells expressing wild-type FUS but appears within 48 h post transfection with FUS-R521C. Co-transfection with scrambled shRNA does not rescue cell death. **b** Kaplan–Meier cumulative survival curves for quantifying cell death indicate transfection of neurons with FUS-R521C significantly reduces cell survival compared with transfection with wild-type FUS. Co-transfection with MBNL1, but not scrambled, shRNA significantly rescues cell death. *N* > 40 cells per group. Data were graphed using SPSS, and log-rank with Mantel–Cox tests was used to compare groups (**P < 0.01, ****P < 0.0001, NS = not significant). **c** Cumulative risk of death corresponding to the survival analysis; low survival in **b** results in higher cumulative risk of death. **d**–**e** Sholl analyses of dendritic morphologies of neurons transfected with FUS-WT or FUS-R521C with or without concurrent knockdown of endogenous mbnl1. **d** Dendrite tracings to highlight changes in branching or elongation. Neurons expressing FUS-R521C show reduced branching and elongation of dendrites compared with cells expressing wild-type FUS. These changes are rescued when endogenous MBNL1 is knocked down. **e** Quantification of the number of intersections at increasing radii from the neuron cell body. Fewer intersections indicate loss of dendritic branching. Consistent with the qualitative changes shown in **d**, neurons expressing FUS-R521C have a significant decrease in the number of intersections compared with cells expressing FUS-WT. This effect is rescued with knockdown of endogenous mbnl1. Two-way ANOVA with Bonferroni’s, Tukey’s, and Sideak’s multiple comparisons tests were performed for all groups (five cells/group) at each of the radii measured. Asterisks indicate the radii measurements with the highest significant difference between the groups being compared and were consistent between all three multiple comparisons tests (**P < 0.01 and ****P < 0.0001).

To directly test whether knockdown of MBNL1 affects FUS toxicity in primary cortical neurons, we looked for survival and cellular morphology in cortical neurons expressing FUS-R521C in the presence or absence of endogenous MBNL1 (Fig. 5). First, we validated if MBNL1 shRNA and scramble could be visualized by fluorescence live imaging microscopy without being toxic to cells (Supplementary Fig. 15a). Using DRAQ7 as a marker of cell death, we measured toxicity on neurons co-transfected with either wild-type or FUS R521C together with MBNL1 shRNA or scramble (Fig. 5a–c)^78^. Neither expression of wild-type FUS nor knockdown of MBNL1 caused toxicity in primary neurons (Fig. 5a, b). On the other hand, neurons expressing FUS-R521C exhibited signs of cell death as early as 48 h post transfection, and depletion of endogenous Mbnl1 mitigated mutant FUS-induced neuronal toxicity. By Kaplan–Meier analysis of survival and associated cumulative risk of death, knockdown of endogenous *Mbnl1* in FUS R521C-expressing neurons significantly increased survival while reducing their cumulative risk of death (Fig. 5b, c). Consistent with our findings in flies, depletion of endogenous MBNL1 did not suppress cell death in neurons expressing TDP-43 or ALS-associated dipeptide repeat proteins (DPRs) compared with controls (Supplementary Fig. 15b, c). By Sholl analysis cells expressing FUS R521C showed a clear reduction in dendritic branching and elongation compared with cells expressing wild-type FUS, and these defects were partially rescued by *Mbnl1* shRNA (Fig. 5d, e)^79^. These results suggest that MBNL1 protects against FUS-associated neuronal toxicity and morphological defects.

Our experiments in *Drosophila* suggested that depletion of endogenous Mbl suppressed the toxicity associated with FUS mutants, but not other ALS-causing genes (Supplementary Fig. 8). To translate these findings in our mammalian cortical neuronal model, we used DRAQ7 staining to measure whether reduction of endogenous mbnl1 would suppress the toxicity caused by TDP-43 or DPRs that have previously been linked to ALS, specifically PA, GR and PR^78^. We found that depletion of endogenous mbnl1 does not suppress cell death in neurons expressing TDP-43 or ALS-associated DPRs, compared with controls (Supplementary Fig. 15b, c).

Next, we performed a Sholl analysis to determine morphological changes of neurons transfected with wild-type and mutant FUS in the presence and absence of endogenous MBNL1 (Fig. 5d, e)^79^. No significant differences in dendrite morphology were observed between cortical neurons expressing wild-type FUS alone or in combination with Mbnl1 shRNA. However, neurons expressing FUS R521C showed a significant reduction in dendritic branching and elongation compared with cells expressing wild-type FUS, suggesting restoration of neuron morphology by Mbnl1 shRNA (Fig. 5d). These results were confirmed by quantification of the morphological characteristics, which indicated that cells expressing FUS R521C had a statistically significant reduction in the number of intersections at increasing radii from the cell body compared with cells expressing wild-type FUS (Fig. 5e). The effect on the number of intersections was dampened by co-transfecting the neurons with Mbnl1 shRNA. These results suggest that MBNL1 regulates the pathological and morphological defects associated with FUS expression in mammalian models by controlling FUS distribution and interaction with stress granules.

### SMN protein trapped in neuronal cytoplasm by mutant FUS is rescued by MBNL1 KD

**Fig. 6:**
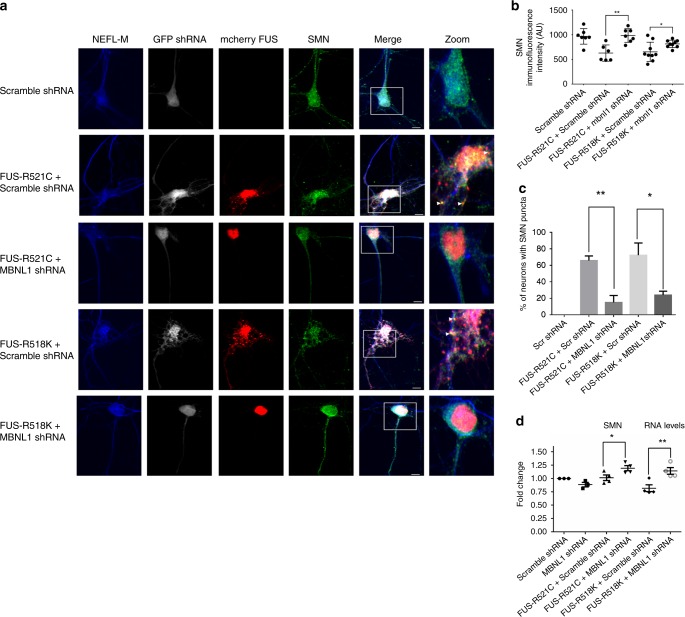
MBNL1 KD prevents mislocalization of axonal SMN protein. Cortical neurons were co-transfected with mCherry tagged mutant FUS constructs (FUS-R518K or FUS-R521C) and either GFP tagged MBNL1 shRNA or scramble shRNA and stained with anti-SMN and anti-NEFL-M. Effect of MBNL1 knockdown on SMN RNA levels was determined by qPCR analysis of NSC-34 cells expressing mutant FUS. **a** Representative confocal images showing SMN and FUS distribution in neuronal cells. NEFL staining was used to trace axons. Labels on the left side of the panel detail which groups were transfected with MBNL1 shRNA or scramble shRNA. White arrows indicate co-localization of mutant FUS and SMN puncta. Scale bars = 5 µm. **b** Quantification of fluorescence intensity of SMN staining along NEFL positive axons confirms that depleting endogenous MBNL1 significantly rescues loss of SMN in mutant FUS neurons. Each graph corresponds to the representative panel above **a**. A minimum of 6–9 neurons were analyzed in biological triplicates for each measurement. Values presented in each graph are means ± SE. Statistical analyses were performed on GraphPad Prism 6 software using two-tailed *t* tests (**P = 0.0014, *P = 0.0331). **c** Quantification of percentage of neurons with cytoplasmic foci containing both FUS and SMN. A minimum of 5–10 cells were analyzed from each group from three independent experiments. Percentage of neurons with FUS and SMN-positive cytoplasmic foci is significantly reduced in mutant FUS neurons (R521C and R518K) co-expressing MBNL1 shRNA compared with cells expressing scramble shRNA two-tailed *t* tests were used for the comparisons in **d** (**P = 0.0072, *P = 0.0352). **d** qPCR analysis from 3–4 biological replicates of NSC-34 cells shows knockdown of mouse endogenous MBNL upregulates SMN RNA in FUS-R518K or FUS-R521C-expressing cells. Statistical analyses were performed on GraphPad Prism 6 software using one-way ANOVA and TUKEY for multiple comparisons (**P = 0.0114, *P = 0.0435).

SMN protein, a known interactor of FUS, has been linked to spinal muscular atrophy (SMA) pathogenesis, and loss of SMN protein leads to RNA splicing defects as well as axonal and synaptic defects in cellular and animal models^80–83^. Human genetic studies supported by experimental models suggest the involvement of SMN in ALS pathogenesis^83^. Interestingly, disease-causing mutations in FUS have been shown to sequester SMN protein in the cytoplasmic puncta of mutant FUS-expressing neurons, thereby reducing SMN levels and mimicking a potential loss of axonal SMN in mammalian primary neurons^80^. In addition, ectopic expression of SMN can suppress mutant FUS-associated axonal defects in primary neurons, suggesting that restoration of SMN is sufficient to reverse the deleterious effects of pathogenic mutations in FUS^80^. We speculated that MBNL1-mediated suppression of FUS toxicity might be linked with SMN protein localization and levels. To test this possibility, we expressed FUS WT, R518K, and R521C in primary cortical neurons and analyzed SMN subcellular localization (Fig. 6, b). As shown by immunofluorescence, SMN was sequestered into puncta containing mutant FUS in neuronal cytoplasm. Interestingly, we observed that knocking down MBNL1 rescued SMN localization by reducing the number of FUS-positive cytoplasmic puncta containing SMN (Fig. 6a, c, Supplementary Fig. 16). We also performed immunoblotting in motor neuron like NSC-34 cells to determine the levels of protein and observed that MBNL1 knockdown significantly increased SMN protein levels in mutant FUS-expressing cells (Supplementary Fig. 17a, b). Furthermore, we also observed a modest increase in Smn RNA levels upon MBNL1 KD in FUS-expressing HEK293T and NSC-34 cells as compared with scramble controls (Fig. 6d, Supplementary Fig. 17c). These findings suggest that MBNL1 knockdown is sufficient to mitigate SMN localization otherwise trapped in the cytoplasmic foci due to pathogenic mutations in FUS.

### MBNL1 KD reduces SGs in mutant FUS-expressing iPSC-derived motor neurons

Human-derived iPSC lines represent a powerful tool to model ALS in vitro and dissect the molecular mechanisms of motor neuron degeneration^84–88^. In fact, iPSC-derived motor neurons have been instrumental for validating genetic modifiers identified in different cellular and animal models^84–91^. We recently utilized CRISPR/Cas9 to generate isogenic iPSC reporter lines with WT and P525L FUS-eGFP^92^. To mimic the heterogeneity in FUS cytoplasmic mislocalization observed in patients, we made two sets of reporters which differed in the length of the linker used to tag the *FUS* C-terminus with eGFP. In particular, in addition to a long and well characterized linker (LL) that does not affect FUS localization, we used a shorter linker (SL) that disrupts the FUS C-terminal domain involved in nuclear relocation, thus causing a basal level of FUS-eGFP mislocalization even in WT FUS cells and exacerbating mislocalization in the mutant counterpart. As expected, WT FUS-eGFP cells carrying the LL localized in the nucleus, whereas isogenic motor neurons with LL P525L FUS-eGFP exhibited a slight cytoplasmic mislocalization. With the SL, we achieved a stronger cytoplasmic mislocalization and accumulation, thus recapitulating the phenotype of motor neurons in advanced ALS pathology^92,93^.

**Fig. 7:**
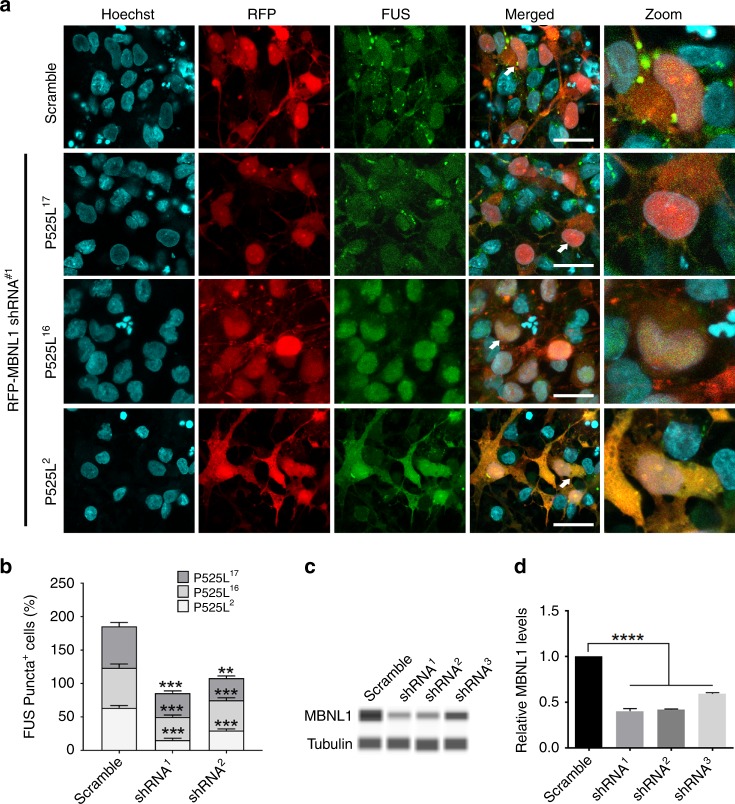
MBNL1 downregulation attenuates FUS-positive SGs in iPSC-derived neurons. We used three different isogenic clones of FUS P525L iPSC line and these isogenic clones are labeled as 2, 16, and 17 as described previously^92^ and were able to generate mixed cultures containing ~ 50% motor neurons (Hb9 + cells). **a** Representative confocal image showing FUS-positive stress granules in control and FUS P525L iPSC-derived neurons. **b** Quantification of the percentage of iPSC-derived neurons showing FUS-positive stress granules following lentiviral delivery of two different MBNL1 shRNAs and scramble control (*n* = 50–60 neurons/line). **c** Protein capillary electrophoresis showing the efficacy of MBNL1 knockdown by three independent MBNL1 shRNAs. **d** Quantification of protein capillary electrophoresis. Statistically significant differences were determined by two-tail, unpaired *t* test in B and two-way ANOVA followed by a Tukey post hoc analysis in **c**: **p ≤ 0.01 are indicated.

To translate our findings in human motor neurons expressing the disease-causing mutation P525L, we differentiated our iPSCs into neurons containing ~ 50% of Hb9 + motor neurons (Supplementary Fig. 19a). To evaluate the impact of MBNL1 on the pathological hallmarks of mutant FUS-expressing neurons, we knocked down endogenous MBNL1 in two independent FUS P525L lines (two SL and one LL clone) and an isogenic control line using scrambled or MBNL1 lentiviral shRNA (Fig. 7, Supplementary Fig. 18). We determined the knockdown efficiency of MBNL1 shRNA constructs and found that two of the tested shRNA vectors allowed ~ 60% knockdown (Fig. 7c, d). We found that FUS P525L mislocalizes in the cytoplasm and incorporates into the SGs as evident from anti-G3BP1 and TIAR staining (Supplementary Fig. 19b). Knocking down endogenous MBNL1 reduced the FUS-positive granules by ~ 50% in iPSC-derived neurons expressing mutant FUS (Fig. 7a, b and Supplementary Fig. 18), further supporting our findings in mammalian primary neurons.

### SMN is a genetic modifier of FUS toxicity

**Fig. 8:**
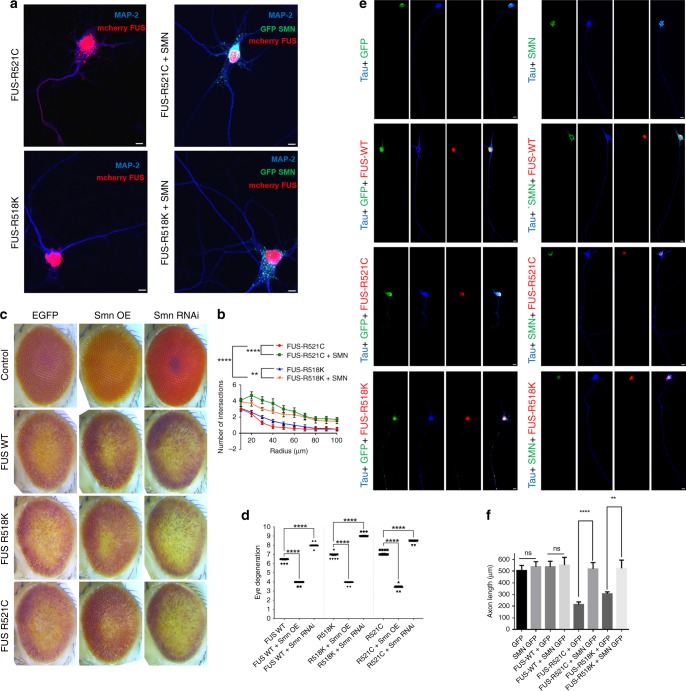
SMN rescues morphological defects and degenerative phenotype in vivo. Cortical neurons were either transfected with FUS-R521C or FUS-R518K alone or co-transfected with SMN and assessed for morphological changes. **a** Representative panel of cortical neurons visualized by confocal microscopy for MAP2 (blue), GFP tagged SMN (green), and mCherry tagged FUS-R521C or FUS-R518K (red). Neurons expressing FUS-R521C and FUS-R518K show reduced branching and elongation of neurites. These changes are rescued when SMN is overexpressed. Scale bars = 5 µm. **b** Quantification of the number of intersections at increasing radii from the neuron cell body. Consistent with the qualitative changes shown in **a**, neurons co-expressing FUS-R521C or FUS-R518K with SMN have a significant increase in the number of intersections compared with mutant FUS neurons (*n* = 10). Two-way ANOVA with Bonferroni’s, Tukey’s and Sideak’s multiple comparisons tests were performed on all groups at each radius measured. Asterisks shown come from the radii measurements with the highest significant difference between the groups being compared and were consistent with all three multiple comparisons tests performed (**P < 0.01 and ****P < 0.0001). **c** Knockdown of endogenous Smn strongly enhanced the FUS toxicity in vivo and ectopic expression of Smn strongly suppressed the FUS-mediated external eye degeneration in flies. **d** Quantification of external eye degenerative phenotype shows that Smn is a strong genetic modifier of FUS toxicity in flies. **e** Motor neurons transfected with GFP, GFP-SMN, mCherry tagged FUS-WT, FUS-R521C or FUS-R518K were assessed for axonal growth defects. Representative panel of motor neurons visualized by confocal microscopy for Tau (blue), GFP tagged SMN (green), and mCherry tagged FUS-WT, FUS-R521C or FUS-R518K (red). Neurons expressing FUS-R521C and FUS-R518K show reduced axonal length as shown by Tau immunostaining. SMN overexpression in mutant FUS motor neurons rescues defects in axon growth. Scale bars = 10 µm. **f** Quantification of the axonal length. Consistent with the qualitative changes shown in **e**, motor neurons co-expressing FUS R521C or FUS-R518K with SMN have a significant rescue in axon length compared with mutant FUS neurons. Student’s *t* test was used to measure statistical significance between FUS neurons co-expressing either GFP or SMN-GFP.

To examine functional link between FUS and SMN, we asked whether modulation of endogenous SMN modifies FUS toxicity in primary cortical neurons and *Drosophila*. We transfected primary cortical neurons with FUS-R521C and FUS-R518K with and without SMN (Fig. 8a, b). As expected, we observed that expression of mutant FUS leads to neurite growth defects with reduced dendritic branching as well as elongation. We found that ectopic expression of SMN significantly reduced the toxicity associated with mutant FUS expression as evident from restoration in dendritic branching and elongation (Fig. 8a, b). Importantly, ectopic expression of SMN significantly reduced incorporation of mutant FUS into G3BP1-positive SGs in primary cortical neurons (Supplementary fig. 17d, e). We also observed that ectopic expression of Smn suppressed the external eye degenerative phenotype associated with FUS expression (Fig. 8c, d). Importantly, knocking down endogenous Smn in flies by RNAi strongly enhanced FUS toxicity, suggesting that SMN is an important determinant of FUS pathology. To examine if mutant FUS overexpression causes axon outgrowth defects as compared with controls, we co-transfected primary motor neurons with GFP, GFP-SMN, and mCherry tagged FUS-WT or R521C or R518K. We found that ectopic expression of FUS R521C and R518K reduced axonal length as evident from anti-tau staining, which is rescued by co-expression of SMN protein (Fig. 8e, f), suggesting that upregulation of SMN is sufficient to protect against mutant FUS-mediated defects. These observations corroborate with our findings showing that MBNL1 KD rescued mutant FUS-mediated dendritic branching defects and neuronal death in primary cortical neurons (Fig. 5).

## Discussion

The heterogeneous clinical and pathological nature of ALS complicates our understanding of its pathogenesis^94–97^. We hypothesized that this variability may be owing to the contribution of genetic modifiers in regulating the toxicity of known ALS-causing genes. Furthermore, differential regulation of these genes may explain differences in the clinical outcomes between patients. To discover novel modifiers of FUS toxicity, we performed an unbiased genetic screen using a Drosophila model that revealed a set of candidate genomic regions that either enhanced or suppressed toxicity caused by mutations in *FUS* when a part of the genomic region was deleted. The *mbl* gene was identified within one of the deficiency regions that suppressed degeneration and was pursued further as a possible modifier of FUS toxicity. Similar to *FUS*, MBNL1 is involved in RNA processing, including alternative splicing^98^, mRNA stability^99^, and RNA trafficking^100^. MBNL1 has previously been linked to several neurological diseases, including myotonic dystrophy^56^, spinocerebellar ataxia^58,101^, fragile X syndrome^57^, and Huntington’s disease^59,60^. However, it has never been directly linked to ALS pathogenesis. Therefore, we performed an array of assays in *Drosophila* to validate whether targeted depletion of Mbl would be sufficient to suppress FUS-associated neurodegeneration. We found that knockdown of endogenous Mbl in *Drosophila* reduced external eye degeneration caused by wild-type and mutant FUS overexpression. Previously, studies have shown increased satellite bouton size and synaptic dysfunctions in fly models of TDP-43^102^. Knocking down endogenous mbl suppressed larval NMJ morphological defects as well as motor impairments, further supporting our finding that muscleblind is a suppressor of FUS toxicity. Using a rat primary cortical neuron model of FUS, we found that shRNA-mediated knockdown of endogenous MBNL1 suppressed cell toxicity and restored dendritic branching. Our in vivo and rat cortical neuronal data suggest that muscleblind is a modifier of FUS toxicity.

There are three *MBNL* (*MBNL1*, *MBNL2,* and *MBNL3*) paralogs in humans^103–105^, all of which code for DNA/RNA-binding proteins involved in RNA trafficking, stability, and alternative splicing^98–100^. In humans and *Drosophila*, muscleblind is essential for proper development and terminal differentiation of neurons^106–108^, skeletal muscle^104,105^, and photoreceptors^109^. MBNL1 has been linked to numerous neurodegenerative diseases and is best known for its involvement in myotonic dystrophy^56–60,101,110^. In myotonic dystrophy, MBNL1 is sequestered into nuclear inclusions owing to uncontrolled binding to abnormal CUG repeat expansions in the 3′-untranslated region of dystrophia myotonica protein kinase mRNA. Reduced availability of functional MBNL1 in the nuclei of muscle cells prevents appropriate splicing of target RNAs, which is thought to be the cause of subsequent muscle-wasting in patients with the disease^104,110,111^. Consistent with the idea that sequestration into nuclear inclusions leads to loss of MBNL function, upregulation of MBNL1 suppressed the effects of the CUG repeat expansion in myotonic dystrophy^112^. On the other hand, our findings support a toxic gain of function model of MBNL1 in FUS-linked ALS, with knockdown of mbl-suppressing toxicity and upregulation of expression having the opposite effect in vivo. Notably, the effect of MBNL in ALS was specific for FUS, as muscleblind did not modify the phenotypes of fly and iPSC models of TDP-43-, VCP-, and C9ORF72 peptide-linked ALS. Rather, our findings in FUS-linked ALS are consistent with previous work performed in a *Drosophila* model for spinocerebellar ataxia type 3, in which overexpression of MBNL1-enhanced ataxin-3 induced neurodegeneration^101^. It has been previously proposed that MBNL1 interacts differently with the CAG repeat expansions of *SCA3* than it does with the CUG repeat expansions associated with other diseases. MBNL1 is capable of binding to both CAG and CUG repeats, though changes in alternative splicing are only observed when MBNL1 is bound to CUG expansions^113,114^. Thus, sequestration of MBNL1 alone is not sufficient for causing aberrant changes in splicing patterns. The modification of FUS toxicity observed in our models following modulation of endogenous muscleblind levels likely occurs through a different mechanism than that of CUG repeat expansion diseases. This may explain why MBNL1 involvement in various disease pathogeneses is tissue-specific.

The deficiency lines used in our initial screen are heterozygous for a number of genes, each of which could be responsible for modifying FUS toxicity. In this study, we focused on muscleblind owing to its association with other human neuronal diseases. Furthermore, RNAi-mediated depletion of other individual genes within the deficiency region did not yield the same suppression of FUS toxicity that was caused by specific knockdown of Mbl (Supplementary Fig. 4). However, suppression of FUS toxicity in the Df(2 R)Exel6066 deficiency line may have been owing to the combined loss of one or more genes in addition to *mbl*. This could be an important future study for identifying additional pathways involved in modifying FUS toxicity in ALS.

Uncontrolled accumulation of mutant FUS into cytoplasmic inclusions is a well-established pathological hallmark of ALS. The inclusions are hypothesized to originate from stress granules that fail to disassemble and most intriguingly, only mutant and not wild-type FUS assembles into SGs upon stress^115–117^. We therefore hypothesized that depletion of muscleblind suppresses FUS toxicity by interacting with SGs. We found that knockdown of endogenous MBNL1 in both human and rat cell culture models reduced FUS-positive cytoplasmic SG formation following exposure to chemical stress. Importantly, we were able to translate our findings from HEK293T and primary cortical neuronal overexpression models to iPSC neurons expressing FUS P525L mutation under native promotor. We observed that knocking down endogenous MBNL1 strongly reduced cytoplasmic FUS-positive puncta. These results support our hypothesis that MBNL1 regulates critical processes required for FUS toxicity in neurons.

Interestingly, depletion of endogenous Mbl in both *Drosophila* and rat primary cortical neurons only affected FUS-associated ALS, and it did not suppress the toxicity caused by other ALS-linked proteins. This observation suggests that the pathway linking muscleblind to FUS is not shared by other ALS-causing proteins. All three members of the FET family of proteins (FUS, EWSR1, and TAF15) specifically bind to RNA from all three human muscleblind-like paralogs in human cells^61^. FUS itself preferentially binds to RNA transcripts with long intronic regions, a characteristic observed for *Drosophila mbl* that is immediately apparent from its size^61^. It is likely that the direct interaction between FUS and *mbl* RNA is the event modulating FUS toxicity, and that depletion of *mbl* RNA affects this interaction to suppress cell death. Intriguingly, we found that endogenous FUS also interacts with MBNL1, suggesting a direct effect of MBNL1 on FUS normal function in a physiological context and toxic gain of function in ALS. Further investigations on how MBNL1 modifies FUS toxicity will improve our understanding of molecular mechanisms driving FUS-ALS pathogenesis.

Reduced levels of SMN protein have been linked with SMA, which, like ALS, is a devastating motor neuron disease^80,81^. There are several reports, suggesting overlapping mechanisms involving defective RNA metabolism in motor neuron diseases, such as ALS and SMA^80–83,118^. However, there are few reports challenging this notion about SMN1 and SMN2 copy number variations in ALS pathogenesis^119,120^. Although FUS is a predominantly nuclear protein that shuttles between the nucleus and cytoplasm, SMN localizes to nuclear Gem bodies and to the cytoplasm where it functions in snRNP biogenesis and RNA splicing^121–125^. Similarly, FUS has been shown to be involved in regulating different aspects of RNA metabolism, including RNA splicing and processing^76,126,127^. Furthermore, SMN localizes to axons where it regulates axonal mRNA transport and local translation^80,128–132^. SMN has been shown to physically interact with FUS WT, and pathogenic mutations in FUS enhance their interaction^80^. Interestingly, pathogenic mutations in FUS have been shown to sequester SMN in the cytoplasmic aggregates of mutant FUS-expressing neurons mimicking a potential mislocalization of SMN. Ectopic expression of SMN was sufficient to suppress mutant FUS toxicity in primary neurons^80^. However, the in vivo relevance of these observations was not known. Our data suggest that reducing the levels of endogenous muscleblind released SMN trapped in mutant FUS-positive cytoplasmic puncta, and upregulation of SMN was sufficient to suppress FUS toxicity in vivo. A recent report found that overexpression of SMN does not modify FUS toxicity in flies and mice^82^. There are few fundamental differences between the two studies. First, they used a fly model that contains four dominant and fully penetrant FUS mutations (R521G, R522G, R524S, and P525L) and each single-point mutation is reported to cause pathogenesis associated with ALS, whereas we used a single ALS-causing mutation in our fly model similar to human patients. There are no reports of presence of all four mutations or any of their combinations in ALS patients, suggesting that compound mutant fly model’s pathophysiological conditions may not be compared with our fly models. Because of this fundamental difference, we believe that a direct comparison between our and the Mirra’s data may not be possible. Notably, we have examined the effect of overexpression of SMN in additional to genetic knockdown of *Smn* in vivo. Our in vivo data show that SMN overexpression suppresses FUS pathogenicity in our fly model of ALS as well as cultured primary neurons. Furthermore, our data demonstrate that muscleblind may be a key player in modulating pathways involved in FUS-SMN.

With this work, we showed a mechanistic link between muscleblind activity and FUS toxicity in both mammalian cellular primary neuronal (cortical and motor neurons) and iPSC models of ALS. Our results are the first to demonstrate that MBNL1 modifies FUS-mediated phenotypes in vivo. Furthermore, these results highlight the role of MBNL1 as a common mediator of neurodegeneration and add ALS to the growing list of diseases. Therefore, this study has a strong impact on understanding the pathogenetic mechanisms underlying ALS as well as other human age-related neurodegenerative diseases.

## Methods

### Drosophila lines

The FUS-WT, FUS-R518K, FUS-R521C, FUS-R521H, and FUS-P525L lines were developed by site-specific insertion of the transgene (site-specific integration lines) at BestGene Inc. using the (attP2) insertion vector. UAS-FUS-WT and FUS-R521H lines generated by random genomic insertion of the transgene (random-insertion lines) were previously described^62^. We are providing details about the lines used in the current manuscript in supplementary table 1–3.

### Genetic screen of *Drosophila*

Genetic screening of *Drosophila spp*. was performed using the DrosDel Isogenic Deficiency Kit (University of Cambridge, Dept. of Genetics, UK), which is a collection of > 450 *Drosophila* lines from the Bloomington Drosophila Stock Center that contain heterozygous deletions of known genomic regions^133,134^. By crossing these lines with our model flies that express a mutant, ALS-linked, human FUS (FUS-R521H) in their eyes^62^, we identified deficiency lines that would either enhance or suppress FUS-associated degeneration of the external eye. We are including a table showing all of the deficiency lines that modified the FUS toxicity in flies.

### Eye degeneration experiments in *Drosophila*

Utilizing the GAL4-UAS system, flies expressing the glass multiple reporter promoter element were crossed with flies expressing wild-type and mutant, exogenous, human FUS at 25°C. Images of the left eyes of F1 generation, adult female *Drosophila* were taken at day 1 (or as indicated for ageing experiments) using a Leica M205C dissection microscope equipped with a Leica DFC450 camera. External eye degeneration was quantified using a previously published scoring system^135^. Statistical analyses were performed using Prism 6 (GraphPad Software). Statistical comparisons between groups were performed using either the Mann–Whitney *U*-test or Kruskal–Wallis test with Dunn’s multiple comparisons test, as indicated.

### Western blotting

*Drosophila*: At day 1, F1 generation, adult, female heads were collected from each cross and snap-frozen on dry ice. Three heads were used for each lane of the western blots. Heads were crushed on dry ice and allowed to incubate in RIPA buffer containing 150 mm NaCl, 1% NP40, 0.1% SDS, 1% sodium deoxycholate, 50 mm NaF, 2 mm EDTA, 1 mm DTT, 0.2 mm Na orthovanadate, 1 × protease inhibitor cocktail (Roche; 11836170001). Lysates were sonicated and centrifuged to remove exoskeletal debris. Supernatants were boiled in Laemmli Buffer (Boston Bioproducts; BP-111R) for 5 min and proteins were separated using 4–12%, NuPAGE bis-tris gels (Novex/Life Technologies). Proteins were transferred onto nitrocellulose membranes (iBlot 2 transfer stacks; Invitrogen, IB23001) using the iBlot2 system (Life Technologies; 13120134). Membranes were blocked in milk (BLOT–QuickBlocker reagent; EMD Millipore; WB57-175GM) and incubated in primary antibody overnight. Blots were washed and incubated for in secondary antibody for 1 h. Imaging was performed using an Odyssey CLx (LI-COR Biosciences). Protein levels were quantified using Image Studio (LI-COR Biosciences), and statistical analyses were performed with Prism 6 (GraphPad Software). All Western blots were performed in triplicate using biological replicates.

*Mammalian cells*: HEK293T and Neuro2a (N2A) cells from ATCC were cultured in advanced Dulbecco’s Modified Eagle Medium (DMEM) (Gibco; 12491023) containing 10% FBS (Biowest; S01520) and 1 × GlutaMAX (Gibco; 35050079). Cells were lysed by boiling for 5 min in 1 × Laemmli (Boston BioProducts; BP-111R) and RIPA buffer. All NuPAGE and western blotting steps were performed as described above. Experiments were performed in triplicate using three independent lysate preparations from cultured cells.

### Western blot analysis for SMN

NSC-34 cells were grown in DMEM (Gibco #11995-065) with 10% heat-inactivated FBS (Gibco # 16140071) and 1% penicillin and streptomycin. Cells were transfected with 250 ng of GFP tagged scramble shRNA or MBNL1 shRNA either alone or combined with 750 ng of cherry tagged FUS. Forty-eight hours post transfection, cell lysates were prepared using RIPA extraction and lysis buffer (Thermo Fisher #89900). Cell lysates were sonicated at 4 °C and denatured at 95 °C in sample buffer (Bio-Rad). Proteins were resolved using 4–20% gels (Bio-Rad, Mini-PROTEAN TGX Precast Protein Gels) and transferred onto a 0.2 µm nitrocellulose membrane (Bio-Rad) using turbo transblot (Bio-Rad) according to manufacturer instructions. The membrane was then blocked for an hour in 5% non-fat dry milk solution followed by incubation with mouse anti-SMN primary antibody (BD Biosciences #610646) at 1:20000 dilution overnight at 4 °C. Following 3 × washes with TBS-tween, membranes were incubated with Amersham ECL Mouse IgG, HRP-linked F(ab’)_2_ fragment from sheep (GE lifesciences # NA9310-1ML) at 1:5000 dilution for an hour at room temperature followed by 3 × washes and developed using ECL solution (Pierce ECL Western Blotting Substrate, Thermofisher #32106) for 3 min before imaging on ChemiDoc Imaging Systems (Bio-rad). The membrane was then incubated in stripping buffer (62.5 mm Tris-HCl, 2% SDS,1 × beta-mercaptoethanol, pH 6.8) for 20 min at 55 °C followed by 3 × washes, incubated with mouse anti-GAPDH (10R-G109A, Fitzgerald) at a dilution of 1:20,000 overnight at 4 °C. Membranes were washed thrice the following day and incubated with secondary antibody and developed using ECL as described above. Experiments were run using six independent lysate preparations from cultured cells.

### Arginine methylation assay

Human embryonic kidney 293 T (HEK293T) cells were cultured as previously described and transfected using polyethylenimine. For western blotting analysis, cells were washed with ice-cold PBS and scraped in 100 ml lysis buffer (150 mm NaCl, 2% sodium dodecyl sulfate, 10 mm Hepes pH 7.4, 2 mm EDTA) plus protease inhibitor cocktail (Roche Diagnostics). Total lysates were sonicated and centrifuged at 13,000 rpm for 10 min at 4 C. Cells lysates were denatured at 95 C in sample buffer and processed for 4–12% sodium dodecyl sulfate–polyacrylamide gel electrophoresis (SDS–PAGE), and electro-transferred onto nitrocellulose membranes (Millipore). Immunoblotting was done in 5% non-fat dry milk dissolved in Tris-buffered saline using the following antibodies: anti-FUS (1:2000, sc-25-540, Santa Cruz); anti-Tubulin (1:10000, Sigma); anti-Calnexin (1:5000, Enzo); anti-Laminin B1 (1:1000, Abcam). Immunoreactivity was detected using IRDye-conjugated Goat Anti-Rabbit or Anti- Mouse IgG (Li-Cor), and visualized using Odissey Imaging System (Li-Cor). Experiments were ran in triplicate using three independent lysate preparations from cultured cells.

### Nuclei/cytoplasm fractionation

For cytosol/nuclear fractionation, cells were washed with ice-cold PBS and collected in PBS 1 ×. Upon centrifugation, pellet was resuspended in solA (20 mm Tris-HCl pH8.0, 50 mm NaCl, 1% NP40, 1 mm DTT with protease inhibitors), incubated on ice for 10 min, and centrifuged at 4000 rpm for 5 min. The cytosolic fraction was collected (supernatant), whereas pellet was resuspended in solB (20 mm Tris-HCl pH 8.0, 0.4 m NaCL, 1% NP40, 1 mm DTT and protease inhibitor) and incubated on ice for 20 min. Supernatant was collected upon centrifugation at 12,000 rpm for 10 min (nuclear extract).

### Quantitative reverse-transcriptase polymerase chain reaction

*Drosophila* tissues or cultured cells were lysed using TRIzol (Ambion; 15596026), and RNA was isolated using a phenol–chloroform extraction method. Following lysis, chloroform was added, and the samples were centrifuged. The upper, aqueous layer was isolated, treated with isopropanol, and centrifuged. The resulting RNA pellets were washed with 75% ethanol, centrifuged, and dried by ambient air. RNA was suspended in RNase-free water. Quantity and purity (260/280 and 260/230 ratios) were determined using a NanoDrop ND-1000 spectrophotometer. RNA quality was assessed by 1% agarose gel electrophoresis using ethidium bromide. The iScript Select cDNA Synthesis Kit (Bio-Rad; 170-8897) was then used to produce cDNA from the RNA samples in an Omn-E PCR machine (Thermo Hybaid). Three RNA extractions were performed from each experimental group to produce cDNA; a sample lacking reverse-transcriptase was used as a control to confirm the absence of genomic DNA. All cDNA samples were ran on 96-well plates (Applied Biosystems; 4306737) on a 7300 Real-Time PCR System (Applied Biosystems) using the Bio-Rad iQ Supermix (170-8862). α-tubulin and GAPDH were used as *Drosophila* and human housekeeping genes, respectively. SMN levels in NSC-34 cells were assessed with a commercially available FAM-MGB TaqMan assay (Thermo Fisher Scientific, Cat#Mm00488315_m1). A VIC-MGB TaqMan probe for actin beta served as normalizer (Thermo Fisher Scientific, Mm02619580_g1). Cycle threshold (CT) values were recorded and analyzed following the comparative (CT) method as previously described^136^ using Prism 6 (GraphPad Software) for statistical analyses.

All primers for qPCR were designed PrimerQuest primer design tool (Integrated DNA Technologies). The Primers were designed with the PrimerTime qPCR Assay tool (Integrated DNA Technologies). IDT PrimeTime qPCR Assays were used as the primer/probe solutions. The primers and probes used for qPCR assays are listed in Supplementary Table 4.

### NMJ analyses and immunofluorescence of *Drosophila*

Wandering 3rd instar, larvae from the F1 generation were rinsed in ice-cold phosphate-buffered saline (Lonza; 17-512 F) and dissected along the dorsal midline. Muscles and NMJs were exposed by removing all tissues except the brain and nerves. The dissected larval pellet was fixed in 4% paraformaldehyde in PBS for 20 min at room temperature, washed with PBS, and then blocked with 5% normal goat serum (NGS) (Abcam; AB7681) in 0.1% PBST (0.1% Triton X-100 in PBS). Larval pellets were then probed with primary antibodies overnight at 4°C, washed several times with 0.1% PBST incubated with secondary antibodies for 2 h at room temperature, and then washed with 0.1% PBST. Both primary and secondary antibody solutions were prepared in 5% NGS in 0.1% PBST. Samples were mounted onto slides using ProLong Gold Antifade mounting reagent (Invitrogen; P36930). Confocal images were acquired using a Fluoview FV1000 confocal laser scanning system equipped with a IX81 microscope (Olympus) and a × 60 oil objective. For the analyses, the innervating muscle 4 of the NMJs on segments A2–A5 were imaged, and the synaptic bouton were quantified. Boutons included in a chain of two or more are considered mature. Satellite boutons are not in a chain and sprout off of a mature bouton or branch. Statistical analyses were performed with Prism 6 (GraphPad Software).

### *Drosophila* motor function

D42-gal4 (motor neuron specific driver), was used to express the selected ALS-related genotypes, FUS-R521C, FUS- R518K, and FUS-WT. 20-25 female *Drosophila* were anesthetized using CO2 and transferred to vials. The recovery time from the anesthetization was 1 h prior to the beginning of the experiment. The *Drosophila* were knocked to the bottom of the vial by tapping each vial against the laboratory bench three times. A photo was taken 3 s after the third tap; three experimental replicates performed for each group. The photos were analyzed using Kinovea video player and the ImageJ Cell Counter function was used to quantify how far the *Drosophila* climbed. The marked *Drosophila* were organized by genotype, vial number, and video number.

### Immunocytochemistry and confocal microscopy

HEK293T cells- HEK293T cells from ATCC were cultured on coverslips coated with poly-l-lysine (Sigma; P4832). Cells were transfected with either MBNL1 shRNA or scramble shRNA (Origene; TL303325) using TurboFect (Thermo Fisher Scientific; R0531) following the manufacturer’s protocol. Cells were washed, incubated with primary antibody, washed again, and incubated in secondary antibody as previously described^42^. Coverslips were mounted onto slides with ProLong Gold Antifade mounting reagent. Cells were imaged with a Zeiss LSM 710 confocal microscope as previously described^42^. Comparisons between groups in all bar graphs were made using GraphPad Prism 6 software and were performed with either *t* tests or by one-way analysis of variances (ANOVAs) with Tukey’s or Dunnet’s multiple comparisons tests, as indicated. *P* < 0.05 was used to determine statistical significance.

### Primary neuronal cultures

Primary neuronal cells were fixed with 4% (volume/volume) paraformaldehyde/sucrose in 1 × PBS for 10 min at room temperature. Neurons were permeabilized with 0.25% triton X-100 in 1 × PBS for 10 min at room temperature and blocked in 10% horse serum, 2% bovine serum albumin (BSA), and 1 × PBS for 1 h at room temperature. Neurons were incubated with primary antibodies at 4°C overnight, incubated with secondary antibodies at room temperature for 1 h, and mounted using ProLong Diamond Antifade Mountant with DAPI (Invitrogen; P36962). Neurons were washed three times with 1 × PBS between each step. Cells were imaged by confocal microscopy (Olympus FV1000) using either a × 40 or × 60 objective. Z-stack images were acquired to form composite 3D images.

### Primary cortical neuronal cultures and transfections

Primary cortical neurons were isolated from E17.5 rat embryos as previously described^137^. Cortical neurons were plated at a density of 125,000/well 24-well plates coated with poly-d-lysine or 12 mm coverslips. At day 12 in vitro (DIV 12), neurons were transfected with 1 μg of total DNA/well using Lipofectamine (Invitrogen) 2000 at a ratio of 1:2.

Primary motor neurons were prepared from E14.5 rat embryos as previously described with few modifications^138^. In brief, 10–20 spinal cords were dissected and mechanically broken into small fragments before being incubated in 0.025% trypsin, followed by DNase. After a 4% w/v BSA cushion, cells were centrifuged for 55 min without brake through a 10.4% (v/v) Optiprep (Nycomed Pharma) cushion. Collected bands were then spun through a 4% w/v BSA cushion. Purified motor neurons were resuspended in complete neurobasal medium (Nb‐C) (Neurobasal [Gibco, 21103], B27 supplement (2%), glutamine (0.25%), 2‐mercaptoethanol (0.1%), horse serum (2%). Cells were plated on poly-lysine and laminin coated coverslips at a density of 15,000 cells/12 mm coverslip. Motor neurons were transfected on DIV 7 with 1 μg of total DNA/coverslip using Lipofectamine 2000 (Invitrogen) at a ratio of 1:2.

### Analysis of neuronal toxicity

Time-lapse live-cell fluorescence microscopy was used to measure neuronal survival as previously described^78^. In brief, neurons were transfected on DIV 12 with 750 ng of mCherry tagged FUS, mCherry tagged TDP-43, FLAG tagged Dipeptide repeats (PR_50_, GR_50_, PA_50_) and 250 ng of GFP tagged mbnl1 or scramble shRNA constructs (Origene; TL510761). For SMN overexpression assays, primary cortical neurons were transfected with GFP tagged SMN (Plasmid #37057, Addgene) using lipofectamine. For co-expression experiments 500 ng each of mCherry tagged FUS-R521C or FUS-R518K and 500 ng tagged SMN were used per well. For all the experiments we kept the amount of FUS DNA consistent (i.e., 500 ng) along with an empty plasmid or SMN-GFP. Therefore, the total DNA concentration was always 1 µg and the ratio of both the plasmids was kept 1:1 to rule out any impact on FUS expression. After 24 h, neurons were labeled with 3 μm DRAQ7 dye (Biostatus), which incorporates into the nuclei of dead cells^139^. Transfected neurons were monitored using an inverted microscope (Nikon Eclipse Ti) equipped with a × 20 objective (numerical aperture = 0.45), a Zyla sCMOS camera (Andor), and an LED light source light (X-cite). The same neurons 24 h were subsequently monitored at 48 h and 72 h. During image acquisition, neurons were maintained at 37°C, 5% CO_2_. NIS Elements software (Nikon) was used to control the excitation and emission filters, stage movements, focusing, and imaging acquisition. Images were analyzed with ImageJ (National Institutes of Health). Dead cells were identified and scored by direct visualization of DRAQ7-positive neurons. A minimum of 100 transfected cells were scored per condition. For survival analysis, death was defined by the time at which a neuron was last imaged devoid of nuclear DRAQ7 signal. Kaplan–Meier survival and Cox proportional hazards analyses were carried out using SPSS 19.0 software, while other statistical analysis was carried out using GraphPad Prism 6. Differences in Kaplan–Meier curves were assessed with the log-rank test. Comparisons between experimental groups in bar graphs were done by one-way ANOVA or *t* test as indicated. Data are expressed as average ± sem. *P* < 0.05 was considered statistically significant.

### Characterization of neuronal morphology using Sholl analysis

To analyze dendrite morphology, neurons were stained for the dendritic protein, MAP2. Fluorescence images were acquired, and neurites were traced using the Simple Neurite Tracer plugin (ImageJ). Dendritic arborization was determined by counting the number of dendritic intersections with concentric circles centered on the soma whose radii regularly increased by 10 μm. Sholl analysis was performed with ImageJ^79^. The number of intersections was plotted as a function of the radial distance from the soma. A minimum of five neurons were analyzed per condition.

### Stress granule induction and analysis

Cellular stress granule (SG) assembly was induced in cultured neurons by sodium arsenite as previously described^42^. In brief, cells were treated with 500 μm sodium arsenite for 90 min at 37°C. Cells were fixed and incubated with the appropriate antibodies (anti-G3BP1 and anti-TIA1). The number of SGs per cell was assessed using the Analyze Particles plugin (ImageJ). G3BP1-positive puncta above the size threshold of 1 μm^2^ were included for analysis.

### Motor neuron cultures and transfections

Primary motor neurons were prepared from E14.5 rat embryos as previously described with few modifications^138^. In brief, 10–20 spinal cords were dissected and mechanically broken into small fragments before being incubated in 0.025% trypsin, followed by DNase. After a 4% w/v BSA cushion, cells were centrifuged for 55 min without brake through a 10.4% (v/v) Optiprep (Nycomed Pharma) cushion. Collected bands were then spun through a 4% w/v BSA cushion. Purified motor neurons were resuspended in complete neurobasal medium (Nb‐C) (Neurobasal [Gibco, 21103], B27 supplement (2%), glutamine (0.25%), 2‐mercaptoethanol (0.1%), horse serum (2%). Cells were plated on poly-lysine and laminin coated coverslips at a density of 15,000 cells/12 mm coverslip. Motor neurons were transfected on DIV 7 with 1 μg of total DNA/coverslip using Lipofectamine 2000 (Invitrogen) at a ratio of 1:2.

### Co-IP and nuclear/cytosolic fractionation

Co-IP procedures were carried out at 4 °C. HEK293T cells were seeded in two 10 cm dishes, at 2.5 × 106 cells per dish. 24 h later, cells were washed with ice-cold PBS, scraped in 2 mL IP buffer (50 mm HEPES, 250 mm NaCl, 5 mm EDTA, 0.1% Nonidet P-40, 2.5 mm sodium orthovanadate, 2.5 mm β-glycerophosphate) plus protease inhibitor cocktail (Roche Diagnostics) and sonicated. Cleared lysates were immunoprecipitated using anti-FUS (5 µg/mg lysate, A300-302A, Bethyl Laboratories, Inc.) overnight at 4°C. We used Dynabeads Protein G superparamagnetic beads from Life Technologies Inc. for IP with anti-FUS (5 µg/mg lysate, A300-302A, Bethyl Laboratories, Inc.). Co-IP proteins were then washed three times in IP buffer, resuspended in sample buffer, boiled, and subjected to 10% SDS–PAGE. Protein interaction was detected with immunoblotting using anti-MBLN1 (3A4, sc-47740, Santa Cruz).

Nuclear-cytosolic fractionation procedures were carried out at 4°C according to the manufacturer’s instructions (NE-PER 78833, Thermo Scientific). Samples were analyzed by SDS–PAGE as described above.

### SMN immunostaining and quantification in neurons

Cultured rat cortical neurons were transfected on DIV10 with appropriate plasmid constructs (mCherry tagged FUS-WT, FUS-R521C, FUS-R518K and GFP tagged scramble shRNA or MBNL1 shRNA). After 48 h of transfection, cells were processed for immunocytochemistry to probe for SMN using mouse anti-SMN (BD Biosciences, Cat# 610647) at 1:150 dilution. Axons were labeled using chicken anti-Neurofilament-M (Novus Biologicals, Cat#NB300-222) at 1:300 dilution. Primary antibodies were incubated at 4 degree overnight. Alexa fluor tagged fluorescent secondary antibodies were used at 1:500 dilutions. Z-stack confocal images were acquired using a × 60 oil immersion objective of a confocal microscope (Olympus). Laser and detector settings were kept constant for all conditions. Maximum intensity projected images were constructed using FIJI. Regions of interest were marked using neurofilament positive immunoreactivity for analysis. Data were analyzed from three independent experiments and 6–9 neurons were analyzed per conditions. SMN immunofluorescence was measured on a scale of 0-4096 pixels.

### Axonal growth analysis

Motor neuron cultures were co-transfected on DIV 7 with mCherry tagged FUS and either GFP or SMN-GFP using lipofectamine 2000. After 48–60 h, cells were fixed and immunostained with a mouse monoclonal anti-tau antibody (Sigma # T9450) at a dilution of 1:500 incubated overnight at 4 °C to specifically identify the axon. After 3 × PBS washes, cells were labeled with Alexa fluor 405 tagged Goat antimouse igG (1:500, Invitrogen # A-31553) for 1 h at room temperature washed 3 × with PBS and mounted with mounting media (Aqua-mount, Lerner Laboratories). Fluorescent Images were captured using a × 20 objective (NA 0.75) of a confocal microscope (Nikon A1R) at a resolution of 2048 × 2048 pixels and imported into Fiji (version 1.52) software. The axonal projection from the cell soma was then measured from the center of the soma using the Simple Neurite Tracer plugin (Longair et al., 2011). A minimum of 10 transfected neurons were analyzed per condition from three independent experiments. Data were analyzed using Graph Pad Prism and Student’s *t* test was used to measure statistical significance between FUS neurons co-expressing either GFP or SMN-GFP.

### Cell Lines

HEK293T (#CRL-3216), Neuro2A (N2A, #CCL-131), PC-12 (# CRL-1721) cell lines were freshly obtained from the ATCC for this study. NSC-34 cell line was provided by Dr Neil Cashman, University of Toronto, Toronto, ON, Canada. All cell lines were maintained and utilized at passages 2–5.

### Antibodies (*Drosophila*, primary neuronal cells, HEK293T, and N2A cell lines)

For doing western blots in Drosophila, the following primary antibodies were used: anti-FUS (Bethyl Laboratories; A300-302A, 1:2000), anti-MBNL1 (Millipore; MabE70, 1:2000), and anti-mbnl1 (Invitrogen; MA1-16967, 1:1000). DyLight fluorescent secondary antibodies (Thermo Fisher Scientific) were used at a concentration of 1:10,000

For the *Drosophila* NMJ analysis, the following primary antibodies were used: Alexa Fluor 488-conjugated goat anti-HRP (Jackson Immuno Research; 123-545-021, 1:200) and mouse anti-DLG 4F3 (DSHB, 1:100). The following secondary antibodies were used: Alexa Fluor 647-conjugated anti-Phalloidin (Invitrogen; A22287, 1:250) and goat antimouse Alexa Fluor 546 (Invitrogen, A11030, 1:500).

The following primary antibodies were used: anti-G3BP1 (Protein Tech; 13057-2-AP, 1:2000) and anti-HA7 (Sigma, H3663, 1:500). Alexa-Fluor fluorescent secondary antibodies (Invitrogen) were used at a concentration of 1:500 for doing Western blots in HEK293 and N2a cell lines.

The following primary antibodies were used for doing immunoblotting in primary neuronal cells: anti-MAP2 (EMD Millipore; AB5622, 1:500), anti-FUS (Proteintech; 11570-1-AP, 1:200), anti-TIA1 (Santa Cruz; SC-1751, 1:250), anti-G3BP1 (Proteintech; D5444, 1:100), anti-MBNL1 (Millipore; MABE70, 1:100), and anti-NEFL (Novus Biologicals; NB300-222, 1:300).

### Lentiviral production

Three lentiviral shRNAs targeting MBNL1 and one scramble shRNA vectors were purchased from Dharmacon, GE healthcare (Cat: V3SH11240) as E. coli glycerol stocks. Following amplification and plasmid isolation, packaging of the transfer vectors was carried out in 293 T cells using a lentiviral packaging system (transfer vector: pCD/NL-BH: pczVSV-G ratio of 4:2:1) in the presence of polyethyleneimine (1 µg/ml) as a transfection reagent. The culture supernatant containing the lentiviral particles was collected for two consecutive days and concentrated via ultracentrifugation for 3.5 h at 4^o^C at 25,500 rpm (SW32 rotor, Beckman Coulter). The virus pellet was then dissolved in PBS and aliquots were frozen at − 80^o^C. pCD/NL-BH and pczVSV-G were generously provided by Dr. Federico Calegari.

### Transduction of neurons and knockdown assay

Neurons were derived from iPSCs as previously described^92,93^. Eight to ten days old motor neurons were infected with lentiviral particles carrying shRNAs. An optimized virus titer of 1:400, supplemented with protamine sulfate (10 µg/ml), was used for infection. After 24 h of infection, culture medium was replaced with fresh maturation medium. RFP, which was expressed from the shRNA vectors, was used to monitor viral infection. On day 3–4 after infection, puromycin selection was carried out and kept for another 6–8 days. Following selection, cells were processed for either confocal imaging or protein isolation.

For fluorescence imaging, cells were fixed using 4% PFA for 20 mins. Hoechst was used for nuclear staining. Confocal images of the infected neurons for FUS-eGFP and shRNA-RFP were acquired using × 40 magnification.

For testing knockdown efficiency, protein levels of MBNL1 were assessed using the ProteinSimple WES device. Alpha-tubulin was used as loading control. One-way ANOVA was used to determine statistical significance and was calculated using GraphPad Prism6 software.

### Reporting summary

Further information on research design is available in the Nature Research Reporting Summary linked to this article.

## Supplementary information

Supplementary Information

Peer Review File

Reporting Summary

## Data Availability

All data generated or analyzed during this study are available from the authors. A reporting summary for this article is available as a Supplementary Information file.

## References

[1] Hardiman O, van den Berg LH, Kiernan MC (2011). Clinical diagnosis and management of amyotrophic lateral sclerosis. Nat. Rev. Neurol..

[2] Gordon PH (2013). Amyotrophic lateral sclerosis: an update for 2013 clinical features, pathophysiology, management and therapeutic trials. Aging Dis..

[3] Logroscino G (2010). Incidence of amyotrophic lateral sclerosis in Europe. J. Neurol. Neurosurg. Psychiatry.

[4] Chio A (2009). Epidemiology of ALS in Italy: a 10-year prospective population-based study. Neurology.

[5] O’Toole O (2008). Epidemiology and clinical features of amyotrophic lateral sclerosis in Ireland between 1995 and 2004. J. Neurol. Neurosurg. Psychiatry.

[6] Alonso A, Logroscino G, Jick SS, Hernan MA (2009). Incidence and lifetime risk of motor neuron disease in the United Kingdom: a population-based study. Eur. J. Neurol..

[7] Fang F (2009). Amyotrophic lateral sclerosis in Sweden, 1991-2005. Arch. Neurol..

[8] Byrne S (2012). Cognitive and clinical characteristics of patients with amyotrophic lateral sclerosis carrying a C9orf72 repeat expansion: a population-based cohort study. Lancet Neurol..

[9] Huisman MH (2011). Population based epidemiology of amyotrophic lateral sclerosis using capture-recapture methodology. J. Neurol. Neurosurg. Psychiatry.

[10] Lee CT (2013). Riluzole and prognostic factors in amyotrophic lateral sclerosis long-term and short-term survival: a population-based study of 1149 cases in Taiwan. J. Epidemiol..

[11] Pugliatti M (2013). Amyotrophic lateral sclerosis in Sardinia, insular Italy, 1995-2009. J. Neurol..

[12] Sreedharan J (2008). TDP-43 mutations in familial and sporadic amyotrophic lateral sclerosis. Science.

[13] Byrne S (2011). Rate of familial amyotrophic lateral sclerosis: a systematic review and meta-analysis. J. Neurol. Neurosurg. Psychiatry.

[14] Talbot Kevin, Ansorge Olaf (2006). Recent advances in the genetics of amyotrophic lateral sclerosis and frontotemporal dementia: common pathways in neurodegenerative disease. Human Molecular Genetics.

[15] Renton AE, Chio A, Traynor BJ (2014). State of play in amyotrophic lateral sclerosis genetics. Nat. Neurosci..

[16] Tan AY, Manley JL (2010). TLS inhibits RNA polymerase III transcription. Mol. Cell Biol..

[17] Yang L, Embree LJ, Tsai S, Hickstein DD (1998). Oncoprotein TLS interacts with serine-arginine proteins involved in RNA splicing. J. Biol. Chem..

[18] Lagier-Tourenne C (2012). Divergent roles of ALS-linked proteins FUS/TLS and TDP-43 intersect in processing long pre-mRNAs. Nat. Neurosci..

[19] Zinszner H (1997). binds RNA in vivo and engages in nucleo-cytoplasmic shuttling. J. Cell Sci..

[20] Neumann M (2006). Ubiquitinated TDP-43 in frontotemporal lobar degeneration and amyotrophic lateral sclerosis. Science.

[21] Couthouis J (2012). Evaluating the role of the FUS/TLS-related gene EWSR1 in amyotrophic lateral sclerosis. Hum. Mol. Genet.

[22] Couthouis J (2011). A yeast functional screen predicts new candidate ALS disease genes. Proc. Natl. Acad. Sci. USA.

[23] Salton M (2011). Matrin 3 binds and stabilizes mRNA. PLoS ONE.

[24] Daigle JG (2013). RNA-binding ability of FUS regulates neurodegeneration, cytoplasmic mislocalization and incorporation into stress granules associated with FUS carrying ALS-linked mutations. Hum. Mol. Genet.

[25] Vance C (2009). Mutations in FUS, an RNA processing protein, cause familial amyotrophic lateral sclerosis type 6. Science.

[26] Kwiatkowski TJ (2009). Mutations in the FUS/TLS gene on chromosome 16 cause familial amyotrophic lateral sclerosis. Science.

[27] Arai T (2006). TDP-43 is a component of ubiquitin-positive tau-negative inclusions in frontotemporal lobar degeneration and amyotrophic lateral sclerosis. Biochem. Biophys. Res. Commun..

[28] Mitchell JC (2013). Overexpression of human wild-type FUS causes progressive motor neuron degeneration in an age- and dose-dependent fashion. Acta Neuropathol..

[29] Xia R (2012). Motor neuron apoptosis and neuromuscular junction perturbation are prominent features in a Drosophila model of Fus-mediated ALS. Mol. Neurodegener..

[30] Huang C (2011). FUS transgenic rats develop the phenotypes of amyotrophic lateral sclerosis and frontotemporal lobar degeneration. PLoS Genet..

[31] Xu YF (2010). Wild-type human TDP-43 expression causes TDP-43 phosphorylation, mitochondrial aggregation, motor deficits, and early mortality in transgenic mice. J. Neurosci..

[32] Chung CY (2013). Identification and rescue of alpha-synuclein toxicity in Parkinson patient-derived neurons. Science.

[33] Corti O, Lesage S, Brice A (2011). What genetics tells us about the causes and mechanisms of Parkinson’s disease. Physiol. Rev..

[34] Lee VM, Trojanowski JQ (2006). Mechanisms of Parkinson’s disease linked to pathological alpha-synuclein: new targets for drug discovery. Neuron.

[35] Springer W, Kahle PJ (2006). Mechanisms and models of alpha-synuclein-related neurodegeneration. Curr. Neurol. Neurosci. Rep..

[36] Singleton AB (2003). alpha-Synuclein locus triplication causes Parkinson’s disease. Science.

[37] Sabatelli M (2013). Mutations in the 3’ untranslated region of FUS causing FUS overexpression are associated with amyotrophic lateral sclerosis. Hum. Mol. Genet..

[38] Forman MS, Trojanowski JQ, Lee VM (2004). Neurodegenerative diseases: a decade of discoveries paves the way for therapeutic breakthroughs. Nat. Med..

[39] Bosco DA (2010). Mutant FUS proteins that cause amyotrophic lateral sclerosis incorporate into stress granules. Hum. Mol. Genet..

[40] Gal J (2011). Nuclear localization sequence of FUS and induction of stress granules by ALS mutants. Neurobiol. Aging.

[41] Dormann D (2010). ALS-associated fused in sarcoma (FUS) mutations disrupt Transportin-mediated nuclear import. Embo J..

[42] Daigle JG (2016). Pur-alpha regulates cytoplasmic stress granule dynamics and ameliorates FUS toxicity. Acta Neuropathol..

[43] Buchan JR, Kolaitis RM, Taylor JP, Parker R (2013). Eukaryotic stress granules are cleared by autophagy and Cdc48/VCP function. Cell.

[44] Dewey CM (2012). TDP-43 aggregation in neurodegeneration: are stress granules the key?. Brain Res..

[45] Ramaswami M, Taylor JP, Parker R (2013). Altered ribostasis: RNA-protein granules in degenerative disorders. Cell.

[46] Freibaum BD, Chitta RK, High AA, Taylor JP (2010). Global analysis of TDP-43 interacting proteins reveals strong association with RNA splicing and translation machinery. J. Proteome Resa..

[47] Figley MD, Bieri G, Kolaitis RM, Taylor JP, Gitler AD (2014). Profilin 1 associates with stress granules and ALS-linked mutations alter stress granule dynamics. J. Neurosci..

[48] Shorter J, Taylor JP (2013). Disease mutations in the prion-like domains of hnRNPA1 and hnRNPA2/B1 introduce potent steric zippers that drive excess RNP granule assembly. Rare Dis..

[49] Anderson P, Kedersha N (2008). Stress granules: the Tao of RNA triage. Trends Biochem Sci..

[50] Buchan JR, Parker R (2009). Eukaryotic stress granules: the ins and outs of translation. Mol. Cell.

[51] Lagier-Tourenne C, Polymenidou M, Cleveland DW (2010). TDP-43 and FUS/TLS: emerging roles in RNA processing and neurodegeneration. Hum. Mol. Genet..

[52] Vance C (2013). ALS mutant FUS disrupts nuclear localization and sequesters wild-type FUS within cytoplasmic stress granules. Hum. Mol. Genet..

[53] Gilks N (2004). Stress granule assembly is mediated by prion-like aggregation of TIA-1. Mol. Biol. Cell.

[54] Shang Y, Huang EJ (2016). Mechanisms of FUS mutations in familial amyotrophic lateral sclerosis. Brain Res..

[55] Mase G (2001). ALS with variable phenotypes in a six-generation family caused by leu144phe mutation in the SOD1 gene. J. Neurol. Sci..

[56] Kanadia RN (2003). A muscleblind knockout model for myotonic dystrophy. Science.

[57] Sellier C (2010). Sam68 sequestration and partial loss of function are associated with splicing alterations in FXTAS patients. EMBO J..

[58] Daughters RS (2009). RNA gain-of-function in spinocerebellar ataxia type 8. PLoS Genet..

[59] Rudnicki DD (2007). Huntington’s disease–like 2 is associated with CUG repeat-containing RNA foci. Ann. Neurol..

[60] Wilburn B (2011). An antisense CAG repeat transcript at JPH3 locus mediates expanded polyglutamine protein toxicity in Huntington’s disease-like 2 mice. Neuron.

[61] Hoell JI (2011). RNA targets of wild-type and mutant FET family proteins. Nat. Struct. Mol. Biol..

[62] Lanson NA (2011). A Drosophila model of FUS-related neurodegeneration reveals genetic interaction between FUS and TDP-43. Hum. Mol. Genet..

[63] Hofweber M (2018). Phase separation of FUS is suppressed by its nuclear import receptor and arginine methylation. Cell.

[64] Yoshizawa T (2018). Nuclear import receptor inhibits phase separation of FUS through binding to multiple sites. Cell.

[65] Qamar S (2018). FUS phase separation is modulated by a molecular chaperone and methylation of arginine cation-pi Interactions. Cell.

[66] Murakami T (2015). ALS/FTD mutation-induced phase transition of FUS liquid droplets and reversible hydrogels into irreversible hydrogels impairs RNP granule function. Neuron.

[67] Guo L (2018). Nuclear-import receptors reverse aberrant phase transitions of RNA-binding proteins with prion-like domains. Cell.

[68] Scaramuzzino C (2013). Protein arginine methyltransferase 1 and 8 interact with FUS to modify its sub-cellular distribution and toxicity in vitro and in vivo. PLoS ONE.

[69] Li YR, King OD, Shorter J, Gitler AD (2013). Stress granules as crucibles of ALS pathogenesis. J. Cell Biol..

[70] Tradewell ML (2012). Arginine methylation by PRMT1 regulates nuclear-cytoplasmic localization and toxicity of FUS/TLS harbouring ALS-linked mutations. Hum. Mol. Genet..

[71] Konieczny P, Stepniak-Konieczna E, Sobczak K (2014). MBNL proteins and their target RNAs, interaction and splicing regulation. Nucleic Acids Res..

[72] Pascual M, Vicente M, Monferrer L, Artero R (2006). The Muscleblind family of proteins: an emerging class of regulators of developmentally programmed alternative splicing. Differentiation.

[73] Wang WY (2013). Interaction of FUS and HDAC1 regulates DNA damage response and repair in neurons. Nat. Neurosci..

[74] Barmada SJ (2015). Amelioration of toxicity in neuronal models of amyotrophic lateral sclerosis by hUPF1. Proc. Natl. Acad. Sci. USA.

[75] Huang C (2012). Entorhinal cortical neurons are the primary targets of FUS mislocalization and ubiquitin aggregation in FUS transgenic rats. Hum. Mol. Genet..

[76] Qiu H (2014). ALS-associated mutation FUS-R521C causes DNA damage and RNA splicing defects. J. Clin. Invest..

[77] Ryu HH (2014). Autophagy regulates amyotrophic lateral sclerosis-linked fused in sarcoma-positive stress granules in neurons. Neurobiol. Aging.

[78] Wen X (2014). Antisense proline-arginine RAN dipeptides linked to C9ORF72-ALS/FTD form toxic nuclear aggregates that initiate in vitro and in vivo neuronal death. Neuron.

[79] Sholl DA (1953). Dendritic organization in the neurons of the visual and motor cortices of the cat. J. Anat..

[80] Groen EJ (2013). ALS-associated mutations in FUS disrupt the axonal distribution and function of SMN. Hum. Mol. Genet..

[81] Sun S (2015). ALS-causative mutations in FUS/TLS confer gain and loss of function by altered association with SMN and U1-snRNP. Nat. Commun..

[82] Mirra A (2017). Functional interaction between FUS and SMN underlies SMA-like splicing changes in wild-type hFUS mice. Sci. Rep..

[83] Gama-Carvalho M (2017). Linking amyotrophic lateral sclerosis and spinal muscular atrophy through RNA-transcriptome homeostasis: a genomics perspective. J. Neurochem..

[84] Yin S (2017). Evidence that C9ORF72 dipeptide repeat proteins sassociate with U2 snRNP to cause Mis-splicing in ALS/FTD patients. Cell Rep..

[85] Silva MC (2016). Human iPSC-derived neuronal model of Tau-A152T frontotemporal dementia reveals Tau-mediated mechanisms of neuronal vulnerability. Stem Cell Rep..

[86] Kramer NJ (2016). Spt4 selectively regulates the expression of C9orf72 sense and antisense mutant transcripts. Science.

[87] Schweizer Burguete A., et al. GGGGCC microsatellite RNA is neuritically localized, induces branching defects, and perturbs transport granule function. *Elife* **4**, e08881 (2015). 10.7554/eLife.08881 PMC4758954 26650351

[88] Freibaum BD (2015). GGGGCC repeat expansion in C9orf72 compromises nucleocytoplasmic transport. Nature.

[89] Lopez-Gonzalez R (2016). Poly(GR) in C9ORF72-Related ALS/FTD Compromises Mitochondrial Function and Increases Oxidative Stress and DNA Damage in iPSC-Derived Motor Neurons. Neuron.

[90] Yang D (2015). FTD/ALS-associated poly(GR) protein impairs the Notch pathway and is recruited by poly(GA) into cytoplasmic inclusions. Acta Neuropathol..

[91] Burguete AS (2015). GGGGCC microsatellite RNA is neuritically localized, induces branching defects, and perturbs transport granule function. Elife.

[92] Marrone L (2018). Isogenic FUS-eGFP iPSC reporter lines enable quantification of FUS stress granule pathology that is rescued by drugs inducing autophagy. Stem Cell Rep..

[93] Reinhardt L (2019). Dual inhibition of GSK3beta and CDK5 protects the cytoskeleton of neurons from neuroinflammatory-mediated degeneration in vitro and in vivo. Stem Cell Rep..

[94] Hardiman O (2017). Amyotrophic lateral sclerosis. Nat. Rev. Dis. Prim..

[95] Al-Chalabi A (2012). The genetics and neuropathology of amyotrophic lateral sclerosis. Acta Neuropathol..

[96] Tripathi VB, Al-Chalabi A (2008). Molecular insights and therapeutic targets in amyotrophic lateral sclerosis. CNS Neurol. Disord. Drug Targets.

[97] Beghi E (2007). The heterogeneity of amyotrophic lateral sclerosis: a possible explanation of treatment failure. Curr. Med Chem..

[98] Ho TH (2004). Muscleblind proteins regulate alternative splicing. EMBO J..

[99] Masuda A (2012). CUGBP1 and MBNL1 preferentially bind to 3’ UTRs and facilitate mRNA decay. Sci. Rep..

[100] Wang ET (2012). Transcriptome-wide regulation of pre-mRNA splicing and mRNA localization by muscleblind proteins. Cell.

[101] Li LB, Yu Z, Teng X, Bonini NM (2008). RNA toxicity is a component of ataxin-3 degeneration in Drosophila. Nature.

[102] Estes PS (2011). Wild-type and A315T mutant TDP-43 exert differential neurotoxicity in a Drosophila model of ALS. Hum. Mol. Genet..

[103] Fardaei M (2002). Three proteins, MBNL, MBLL and MBXL, co-localize in vivo with nuclear foci of expanded-repeat transcripts in DM1 and DM2 cells. Hum. Mol. Genet..

[104] Miller JW (2000). Recruitment of human muscleblind proteins to (CUG)(n) expansions associated with myotonic dystrophy. EMBO J..

[105] Squillace RM, Chenault DM, Wang EH (2002). Inhibition of muscle differentiation by the novel muscleblind-related protein CHCR. Dev. Biol..

[106] Kania A (1995). P-element mutations affecting embryonic peripheral nervous system development in Drosophila melanogaster. Genetics.

[107] Prokopenko SN, He Y, Lu Y, Bellen HJ (2000). Mutations affecting the development of the peripheral nervous system in Drosophila: a molecular screen for novel proteins. Genetics.

[108] Artero R (1998). The muscleblind gene participates in the organization of Z-bands and epidermal attachments of Drosophila muscles and is regulated by Dmef2. Dev. Biol..

[109] Begemann G (1997). muscleblind, a gene required for photoreceptor differentiation in Drosophila, encodes novel nuclear Cys3His-type zinc-finger-containing proteins. Development.

[110] Llamusi B, Artero R (2008). Molecular effects of the CTG repeats in mutant dystrophia myotonica protein kinase gene. Curr. Genomics.

[111] Jiang H, Mankodi A, Swanson MS, Moxley RT, Thornton CA (2004). Myotonic dystrophy type 1 is associated with nuclear foci of mutant RNA, sequestration of muscleblind proteins and deregulated alternative splicing in neurons. Hum. Mol. Genet..

[112] Kanadia RN (2006). Reversal of RNA missplicing and myotonia after muscleblind overexpression in a mouse poly(CUG) model for myotonic dystrophy. Proc. Natl. Acad. Sci. USA.

[113] Ho TH (2005). Colocalization of muscleblind with RNA foci is separable from mis-regulation of alternative splicing in myotonic dystrophy. J. Cell Sci..

[114] Lee JE, Cooper TA (2009). Pathogenic mechanisms of myotonic dystrophy. Biochem Soc. Trans..

[115] Matus S, Bosco DA, Hetz C (2014). Autophagy meets fused in sarcoma-positive stress granules. Neurobiol. Aging.

[116] Sama RR (2013). FUS/TLS assembles into stress granules and is a prosurvival factor during hyperosmolar stress. J. Cell Physiol..

[117] Lenzi J (2015). ALS mutant FUS proteins are recruited into stress granules in induced pluripotent stem cell-derived motoneurons. Dis. Model. Mech..

[118] Chi B (2018). The neurodegenerative diseases ALS and SMA are linked at the molecular level via the ASC-1 complex. Nucleic Acids Res..

[119] Crawford TO, Skolasky RL (2002). The relationship of SMN to amyotrophic lateral sclerosis. Ann. Neurol..

[120] Gamez J (2002). Survival and respiratory decline are not related to homozygous SMN2 deletions in ALS patients. Neurology.

[121] Pellizzoni L, Yong J, Dreyfuss G (2002). Essential role for the SMN complex in the specificity of snRNP assembly. Science.

[122] Pellizzoni L, Baccon J, Charroux B, Dreyfuss G (2001). The survival of motor neurons (SMN) protein interacts with the snoRNP proteins fibrillarin and GAR1. Curr. Biol..

[123] Pellizzoni L, Charroux B, Rappsilber J, Mann M, Dreyfuss G (2001). A functional interaction between the survival motor neuron complex and RNA polymerase II. J. Cell Biol..

[124] Pellizzoni L, Charroux B, Dreyfuss G (1999). SMN mutants of spinal muscular atrophy patients are defective in binding to snRNP proteins. Proc. Natl. Acad. Sci. USA.

[125] Pellizzoni L, Kataoka N, Charroux B, Dreyfuss G (1998). A novel function for SMN, the spinal muscular atrophy disease gene product, in pre-mRNA splicing. Cell.

[126] Reber S (2016). Minor intron splicing is regulated by FUS and affected by ALS-associated FUS mutants. EMBO J..

[127] Rogelj B (2012). Widespread binding of FUS along nascent RNA regulates alternative splicing in the brain. Sci. Rep..

[128] Jablonka S, Sendtner M (2017). Developmental regulation of SMN expression: pathophysiological implications and perspectives for therapy development in spinal muscular atrophy. Gene Ther..

[129] Sanchez G (2013). A novel function for the survival motoneuron protein as a translational regulator. Hum. Mol. Genet..

[130] Jablonka S, Rossoll W, Schrank B, Sendtner M (2000). The role of SMN in spinal muscular atrophy. J. Neurol..

[131] Fallini C, Bassell GJ, Rossoll W (2012). Spinal muscular atrophy: the role of SMN in axonal mRNA regulation. Brain Res..

[132] Fallini C (2011). The survival of motor neuron (SMN) protein interacts with the mRNA-binding protein HuD and regulates localization of poly(A) mRNA in primary motor neuron axons. J. Neurosci..

[133] Ryder E (2004). The DrosDel collection: a set of P-element insertions for generating custom chromosomal aberrations in Drosophila melanogaster. Genetics.

[134] Ryder E (2007). The DrosDel deletion collection: a Drosophila genomewide chromosomal deficiency resource. Genetics.

[135] Pandey UB (2007). HDAC6 rescues neurodegeneration and provides an essential link between autophagy and the UPS. Nature.

[136] Schmittgen TD, Livak KJ (2008). Analyzing real-time PCR data by the comparative C(T) method. Nat. Protoc..

[137] Kayser MS, McClelland AC, Hughes EG, Dalva MB (2006). Intracellular and trans-synaptic regulation of glutamatergic synaptogenesis by EphB receptors. J. Neurosci..

[138] Kia A, McAvoy K, Krishnamurthy K, Trotti D, Pasinelli P (2018). Astrocytes expressing ALS-linked mutant FUS induce motor neuron death through release of tumor necrosis factor-alpha. Glia.

[139] Edward R (2012). Red/far-red fluorescing DNA-specific anthraquinones for nucl:cyto segmentation and viability reporting in cell-based assays. Methods Enzymol..

