# Supplementary material for: Muscleblind acts as a modifier of FUS toxicity by modulating stress granule dynamics and SMN localization
Source: Nat Commun. 2019 Dec 6;10:5583. doi: 10.1038/s41467-019-13383-z (PMC6898697; doi:10.1038/s41467-019-13383-z)
Supplement: Supplementary file 1 — Supplementary Information [file 41467_2019_13383_MOESM1_ESM.pdf]

Muscleblind acts as a modifier of FUS toxicity by modulating stress granule dynamics and SMN localization

Casci et al.,

Supplementary Figure 1

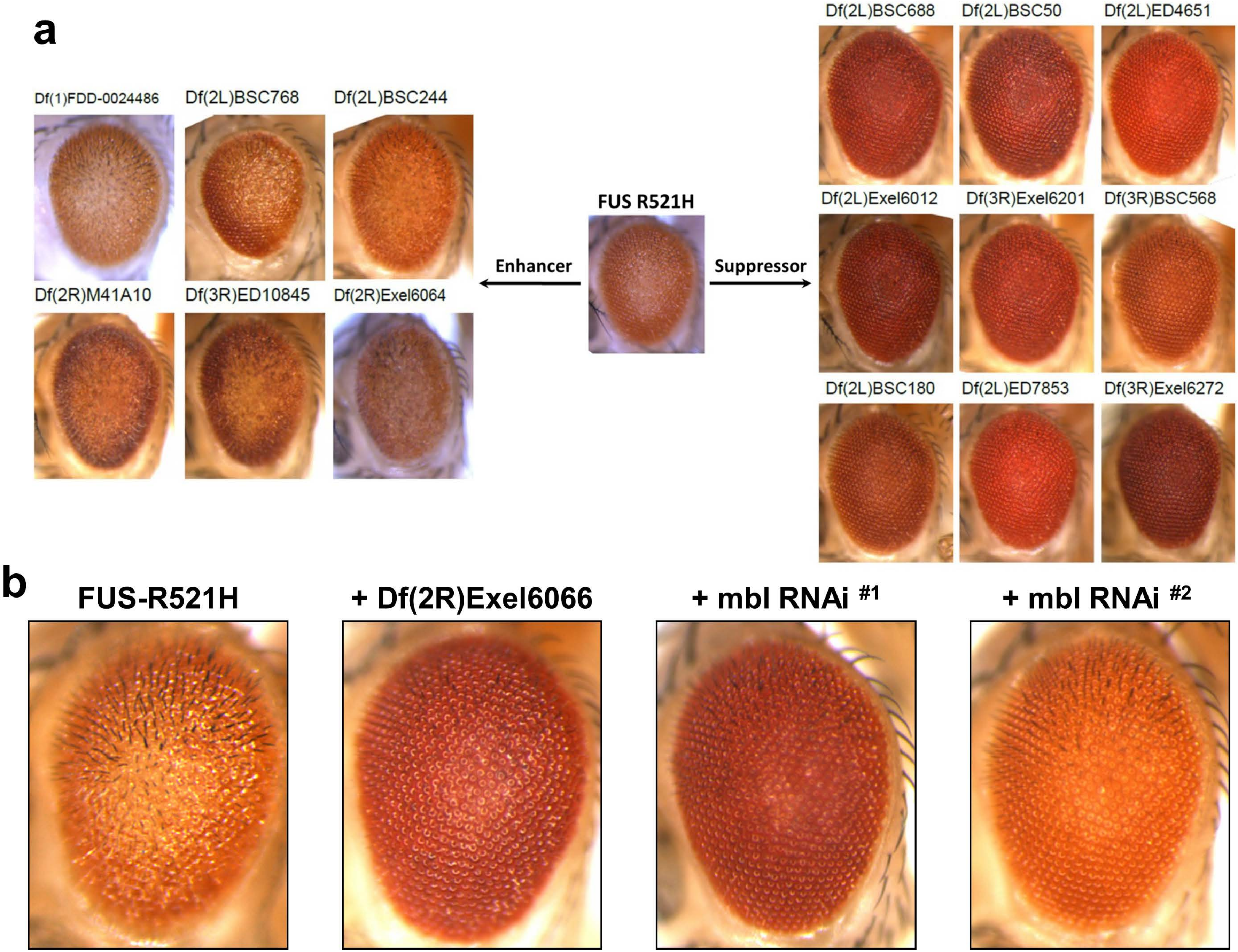

Supplementary Figure 2

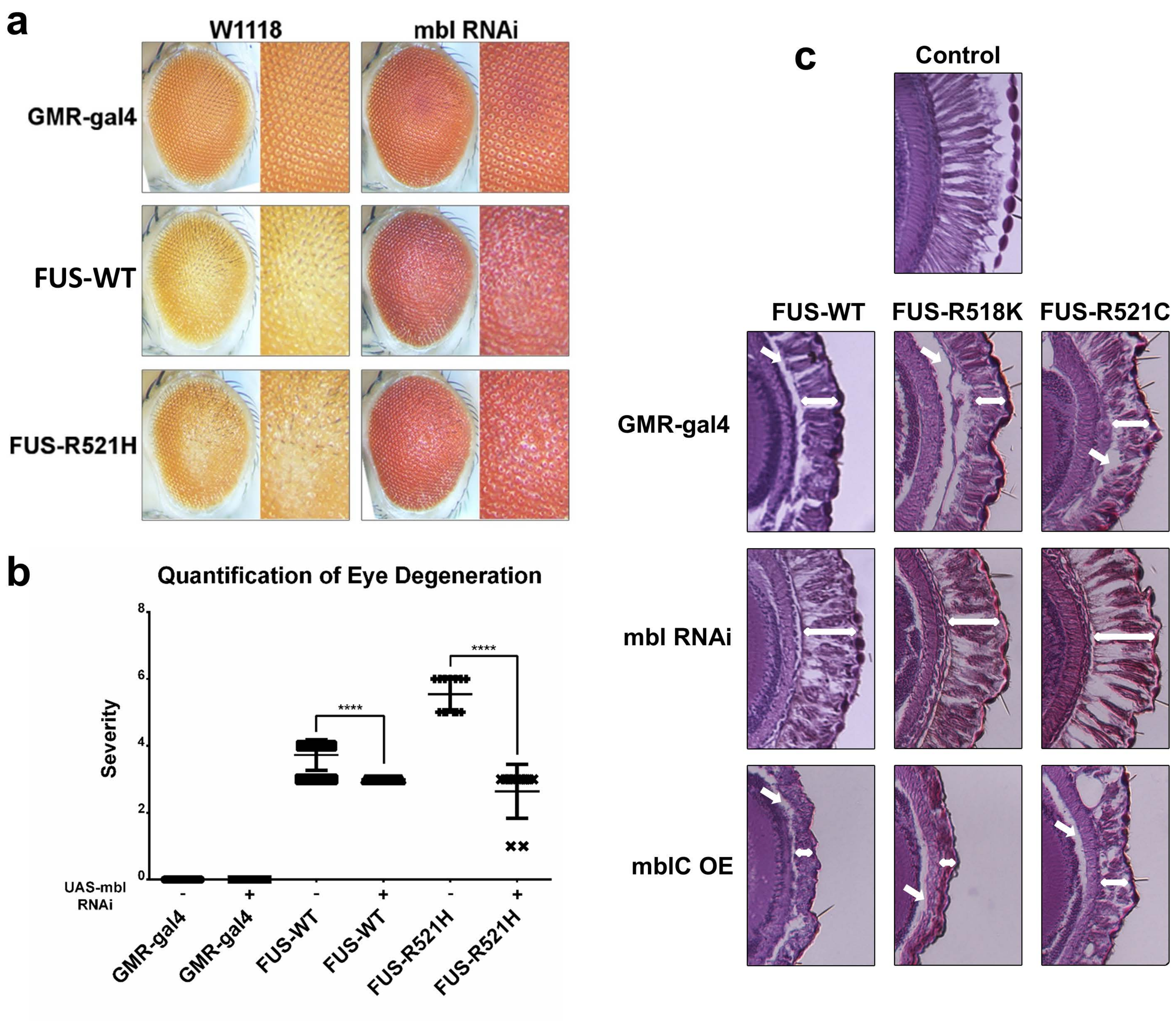

Supplementary Figure 3

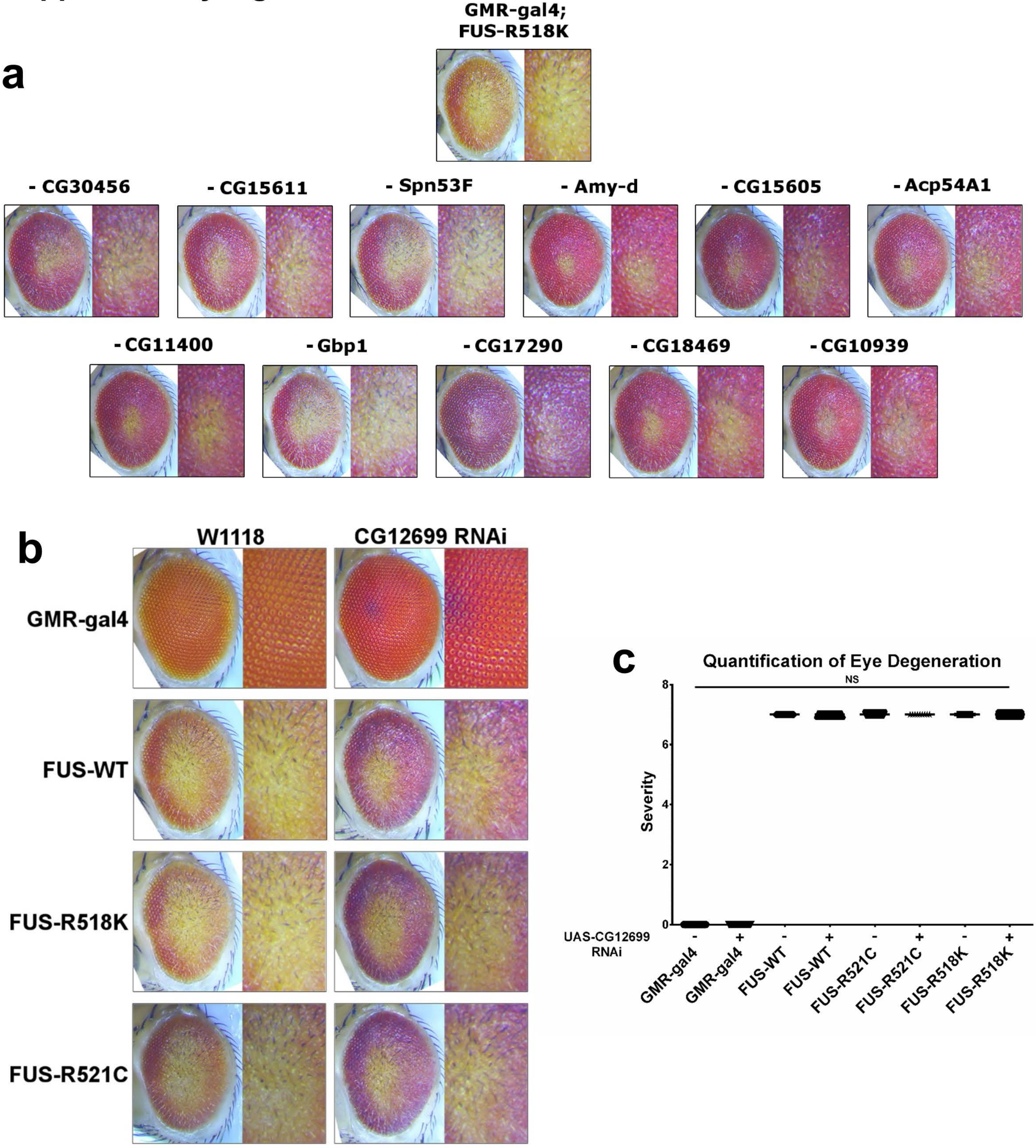

Supplementary Figure 4

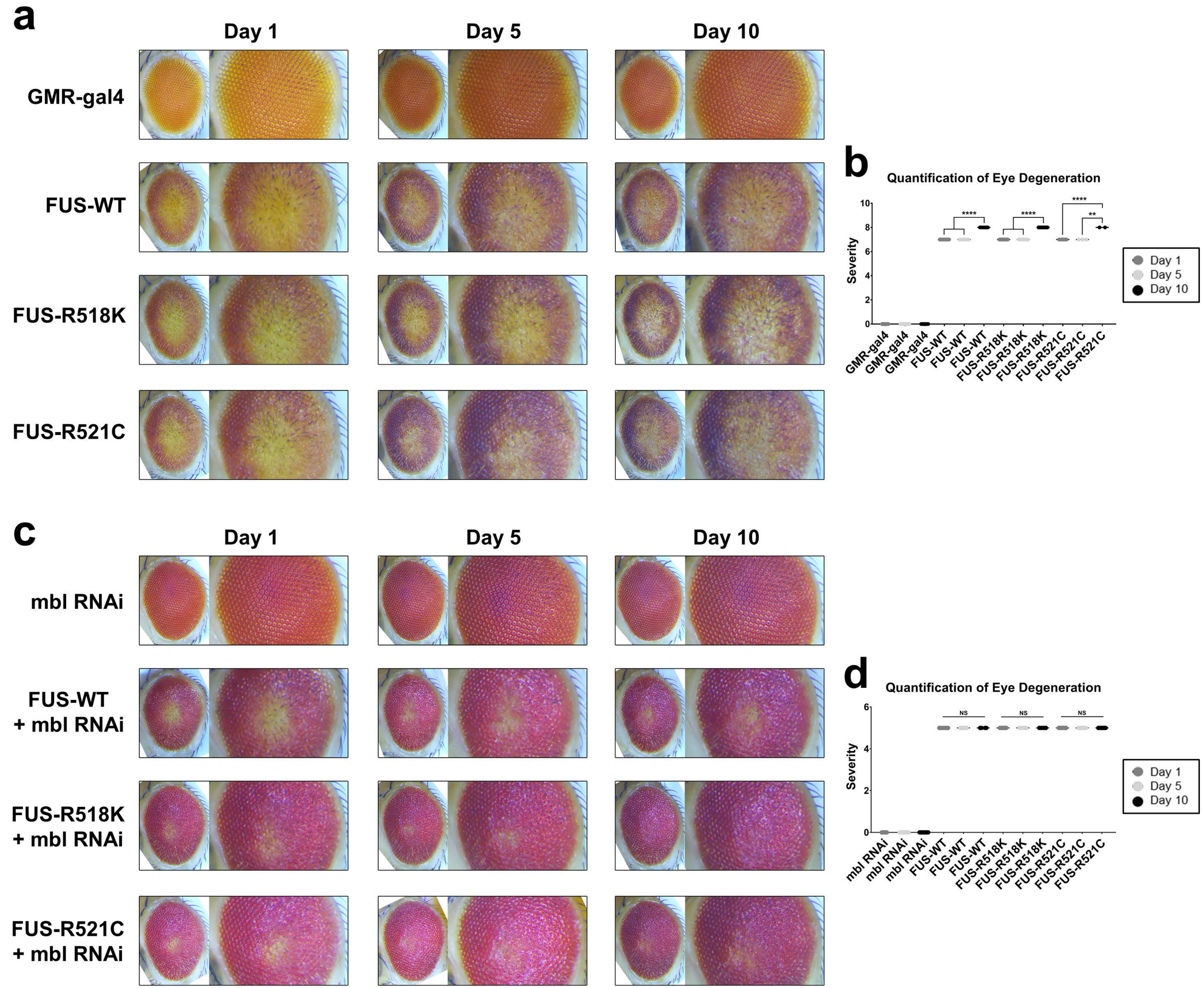

Supplementary Figure 5

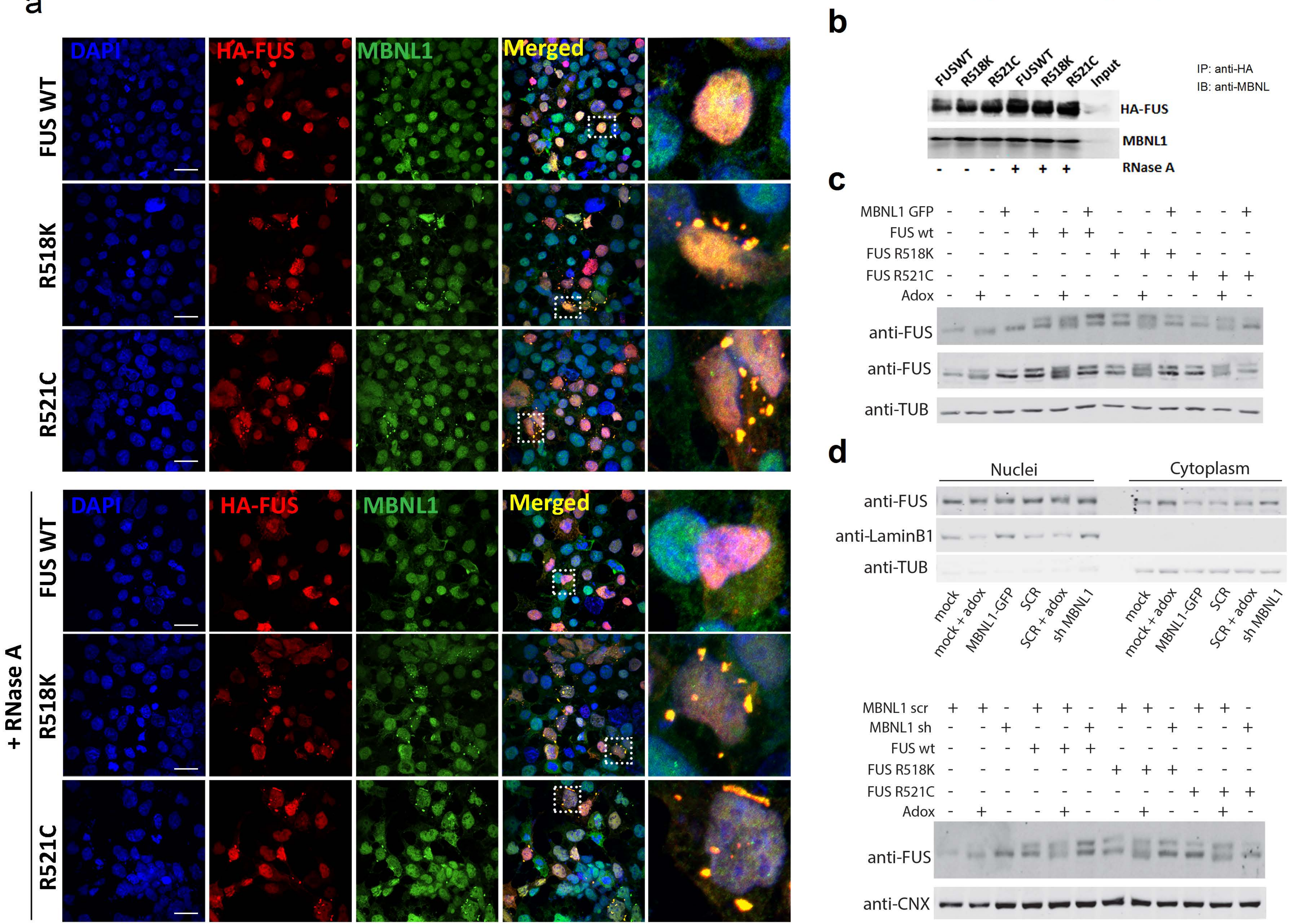

Supplementary Figure 6

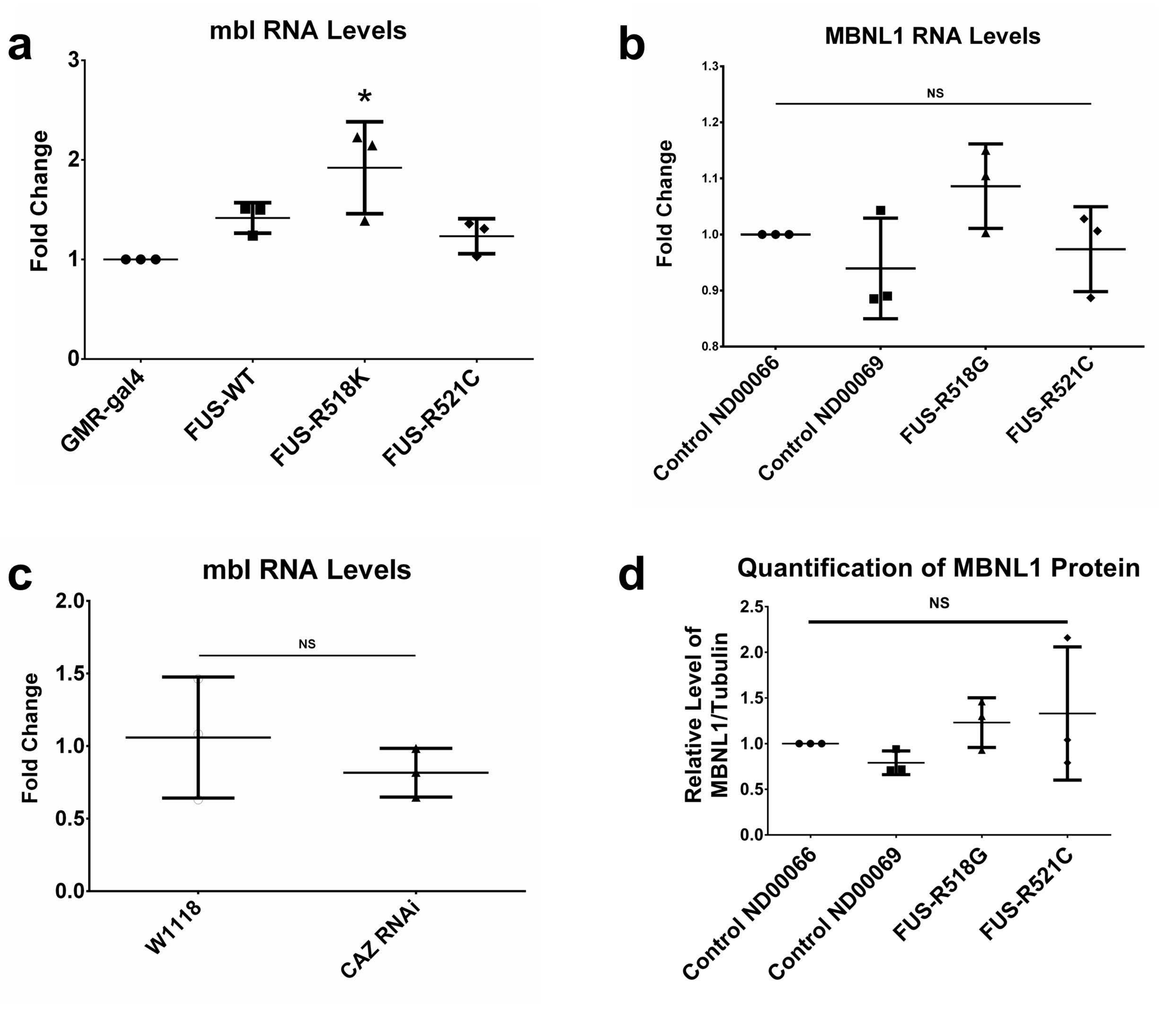

Supplementary Figure 7

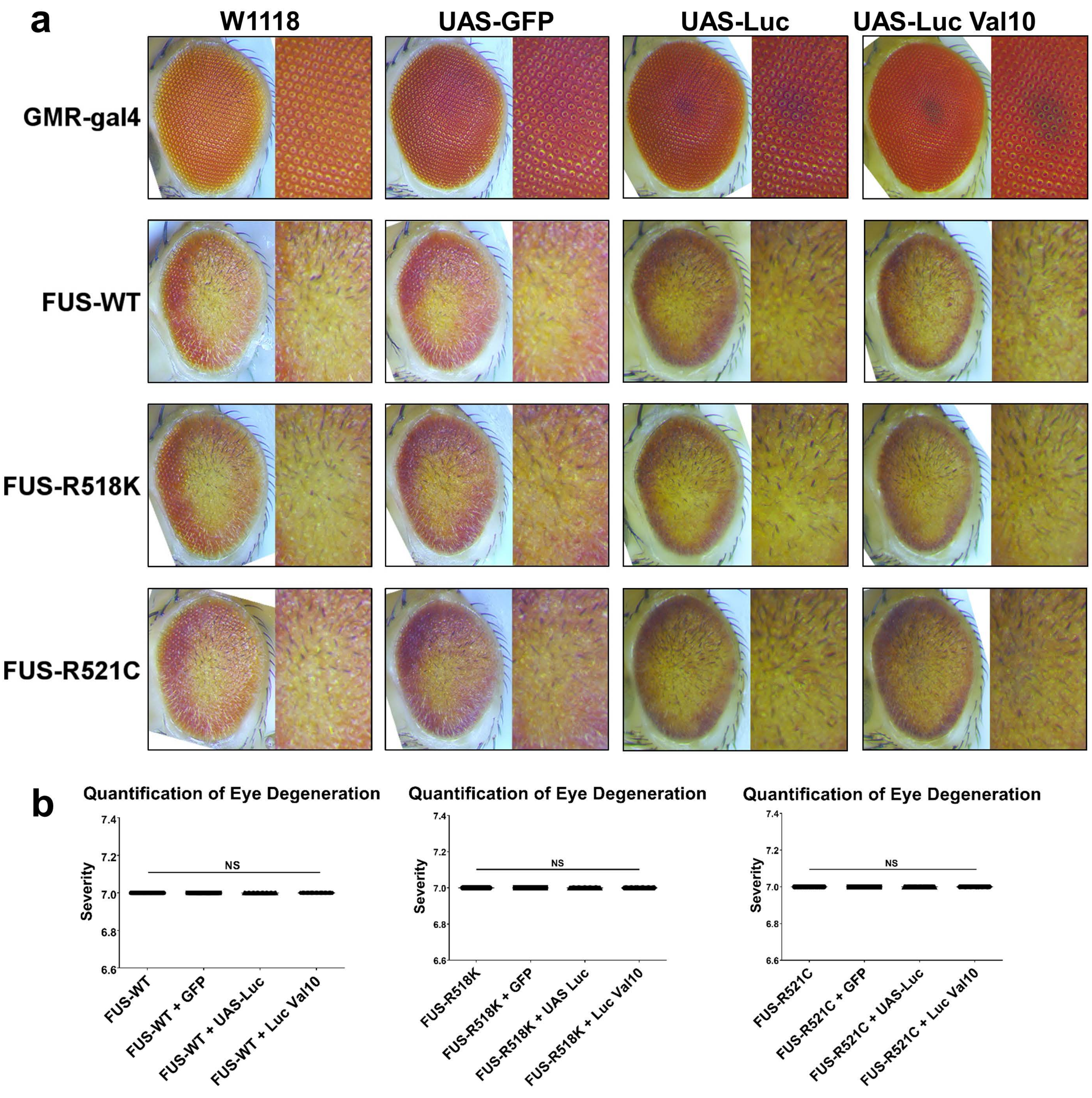

Supplementary Figure 8

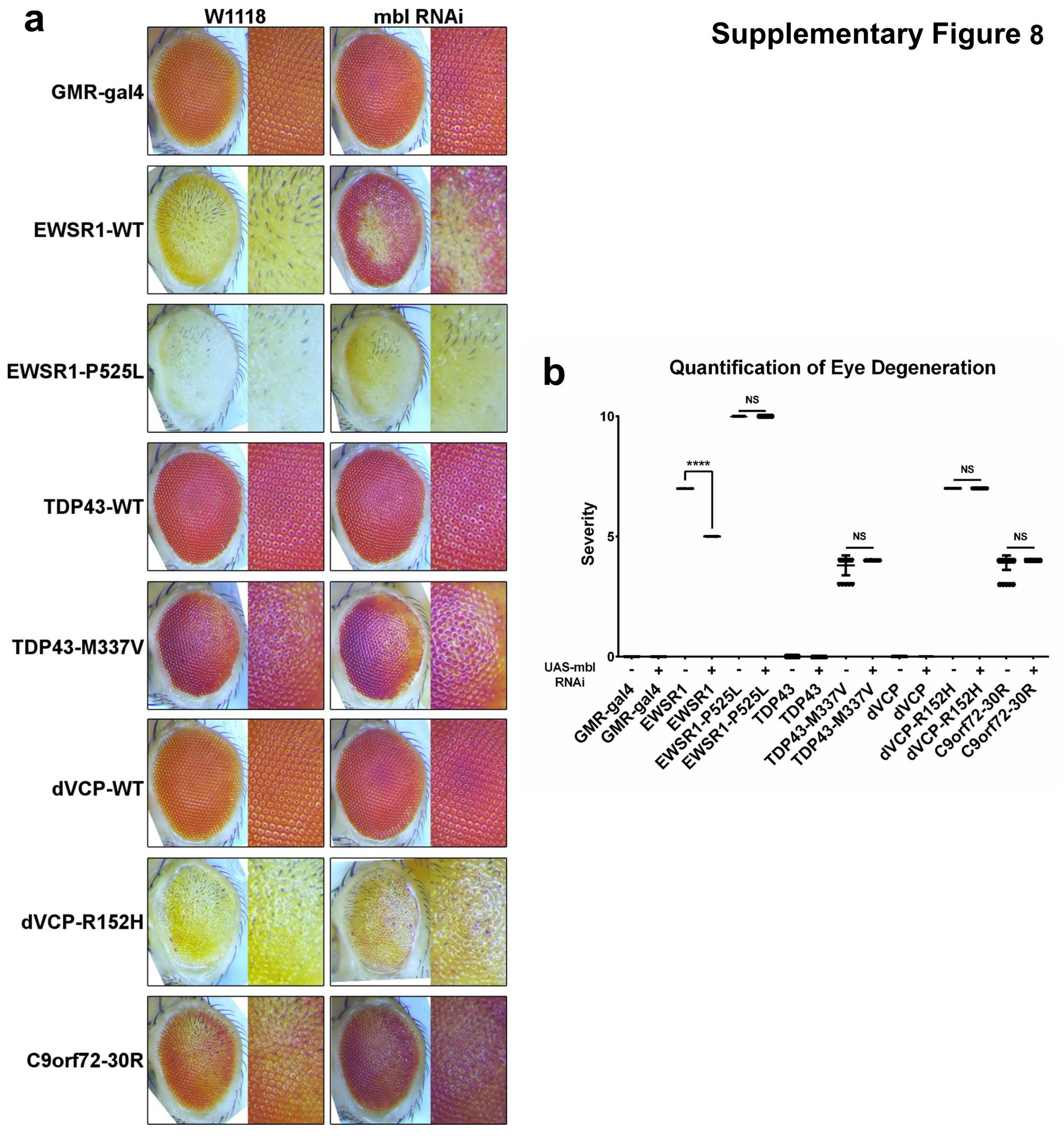

Supplementary Figure 9

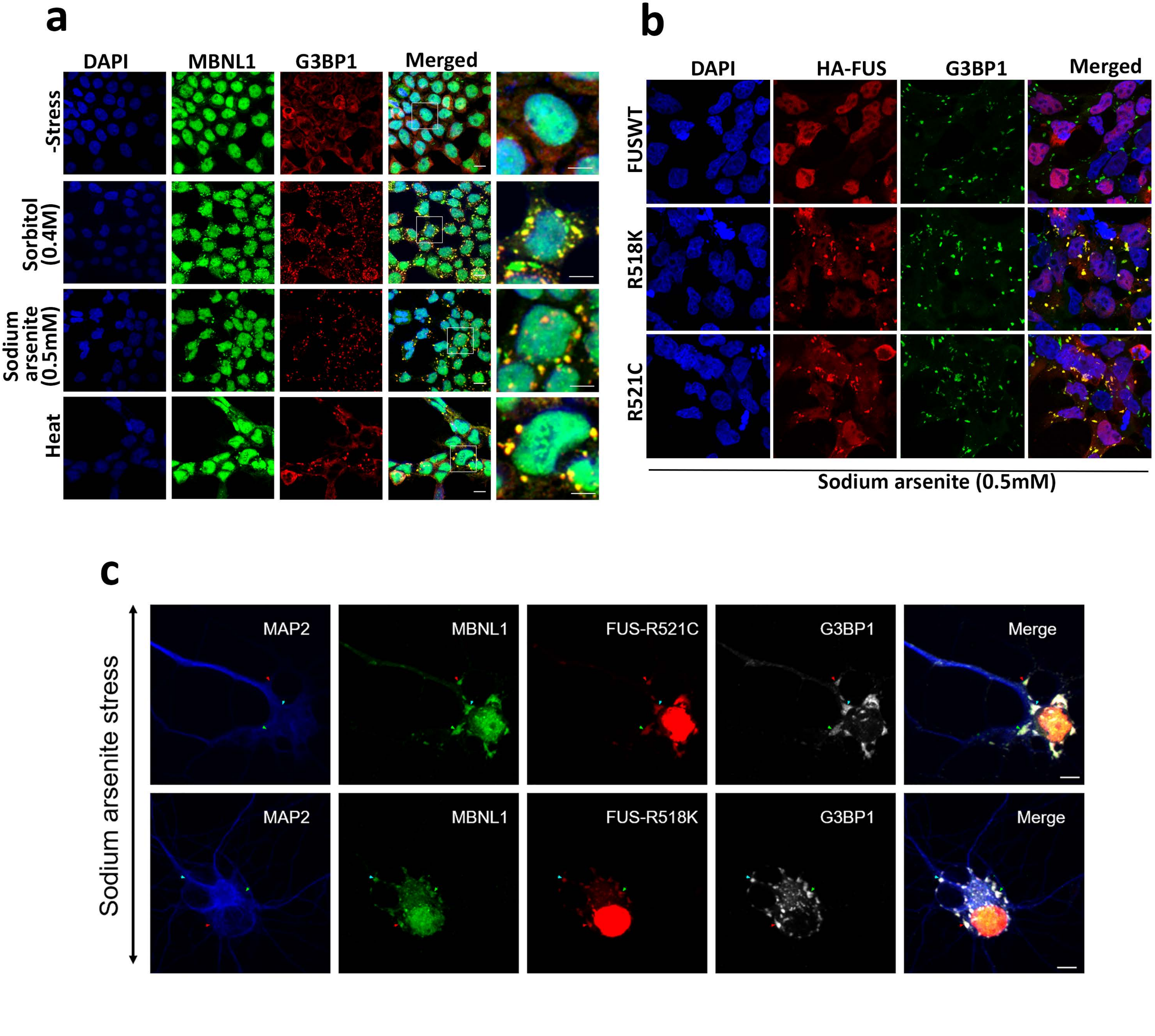

Supplementary Figure 10

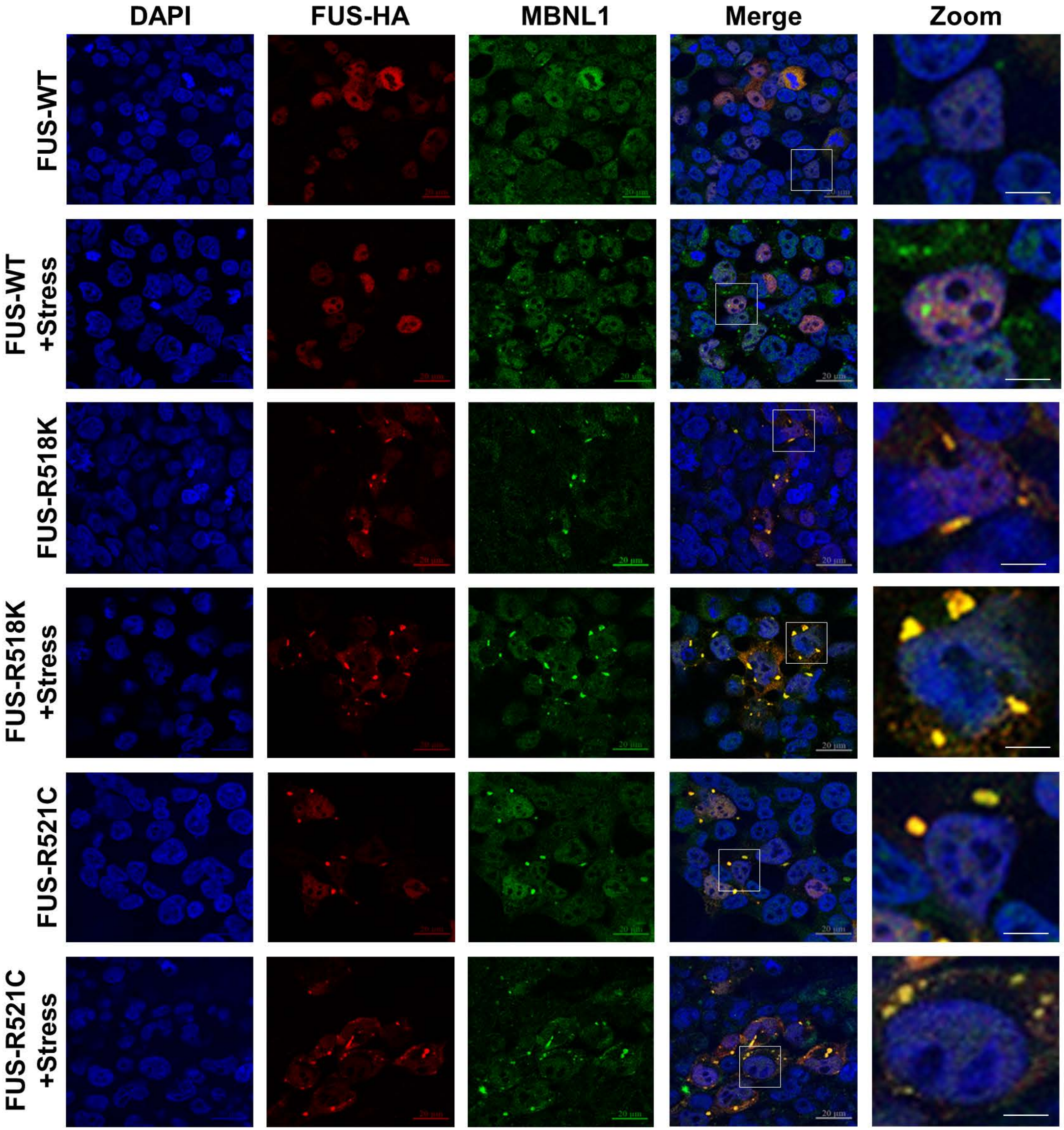

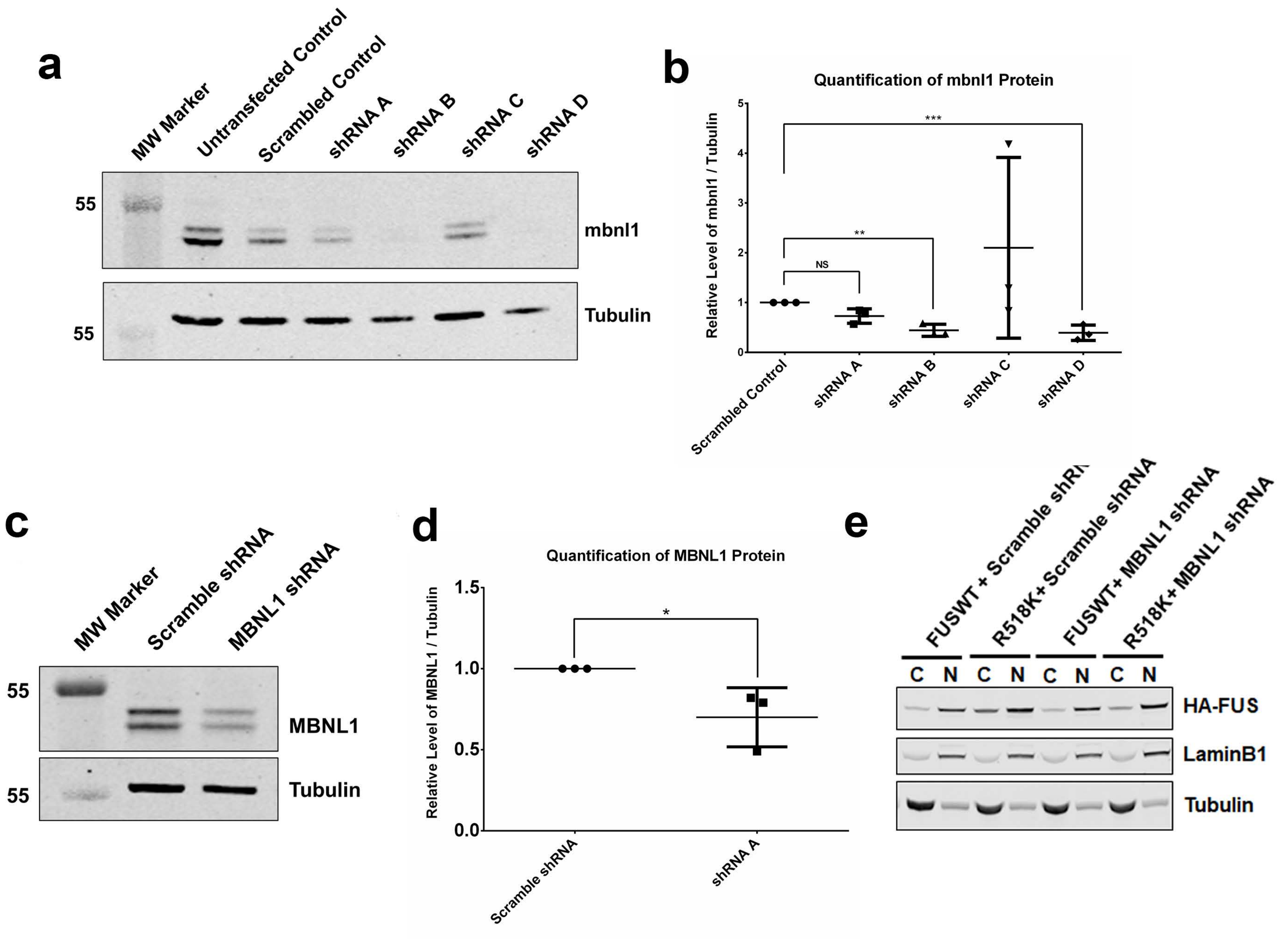
a
MW Marker
Untransfected Control
Scrambled Control
shRNA A
shRNA B
shRNA C
shRNA D
55
mbnl1
55
Tubulin
b
Quantification of mbnl1 Protein
Relative Level of mbnl1 / Tubulin
***
**
NS
Scrambled Control
shRNA A
shRNA B
shRNA C
shRNA D
c
MW Marker
Scramble shRNA
MBNL1 shRNA
55
MBNL1
55
Tubulin
d
Quantification of MBNL1 Protein
Relative Level of MBNL1 / Tubulin
*
Scramble shRNA
shRNA A
e
FUSWT + Scramble shRNA
R518K+ Scramble shRNA
FUSWT+ MBNL1 shRNA
R518K+ MBNL1 shRNA
C N C N C N C N
HA-FUS
LaminB1
Tubulin

Supplementary Figure 12

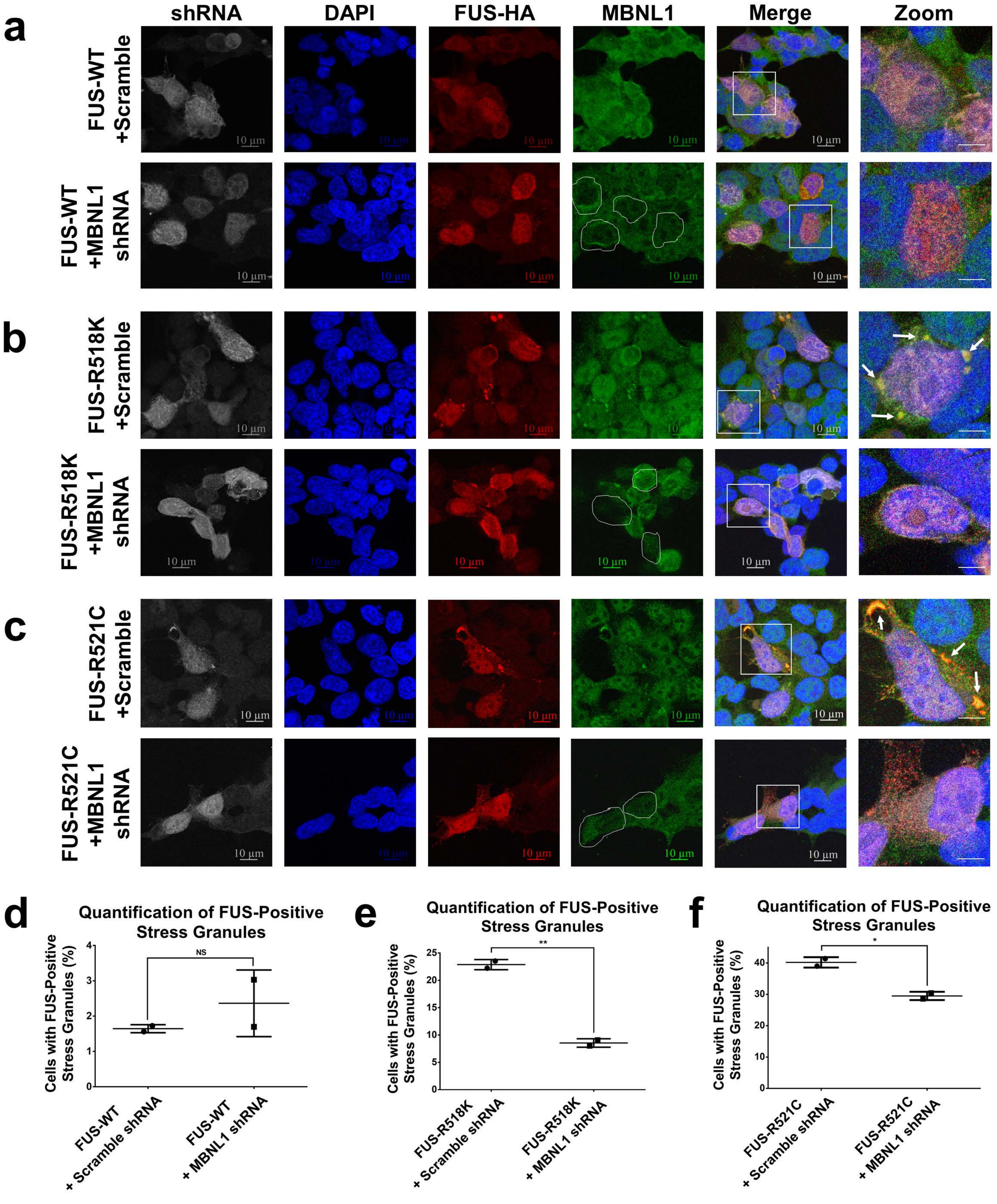

Supplementary Figure 13

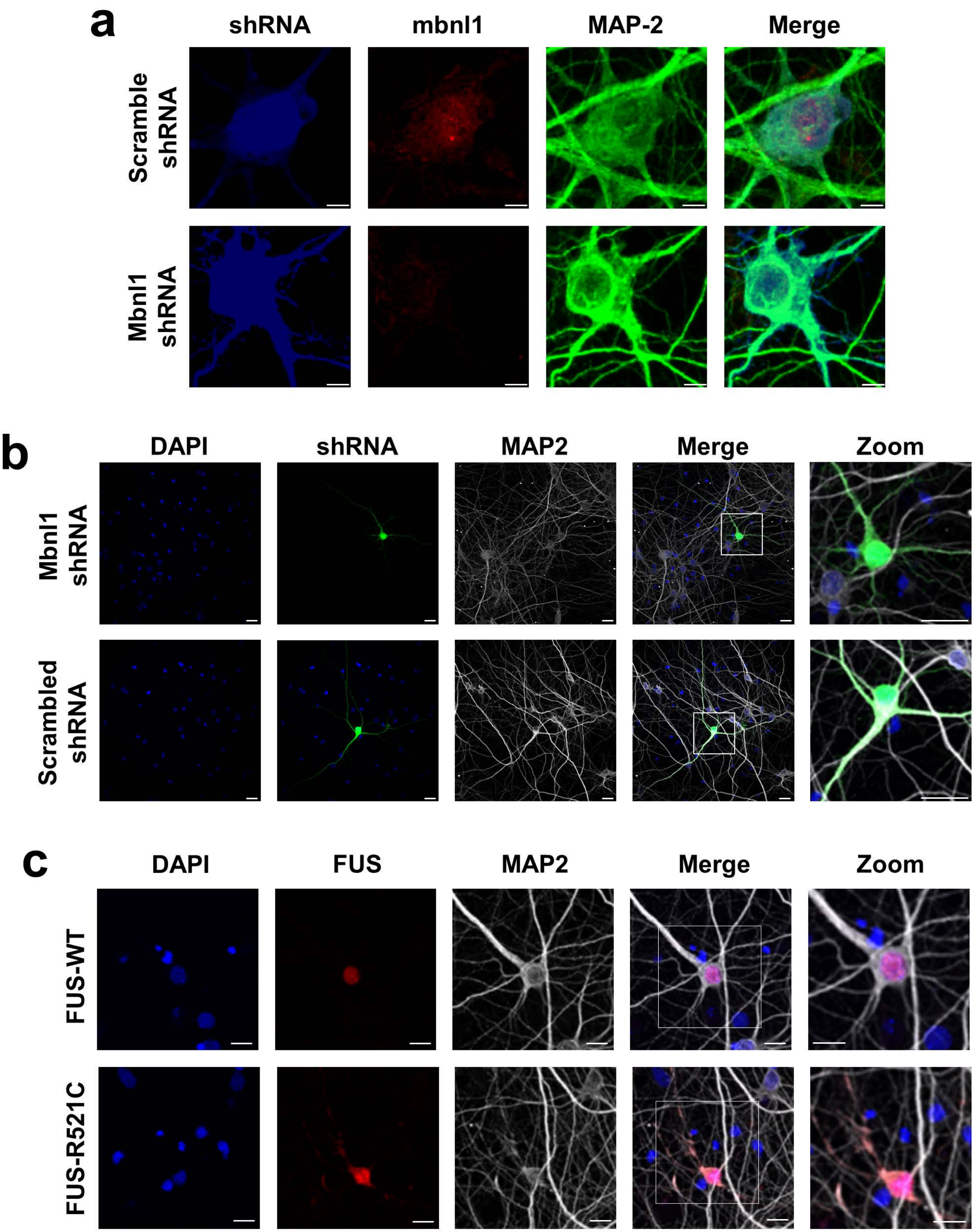

Supplementary Figure 14

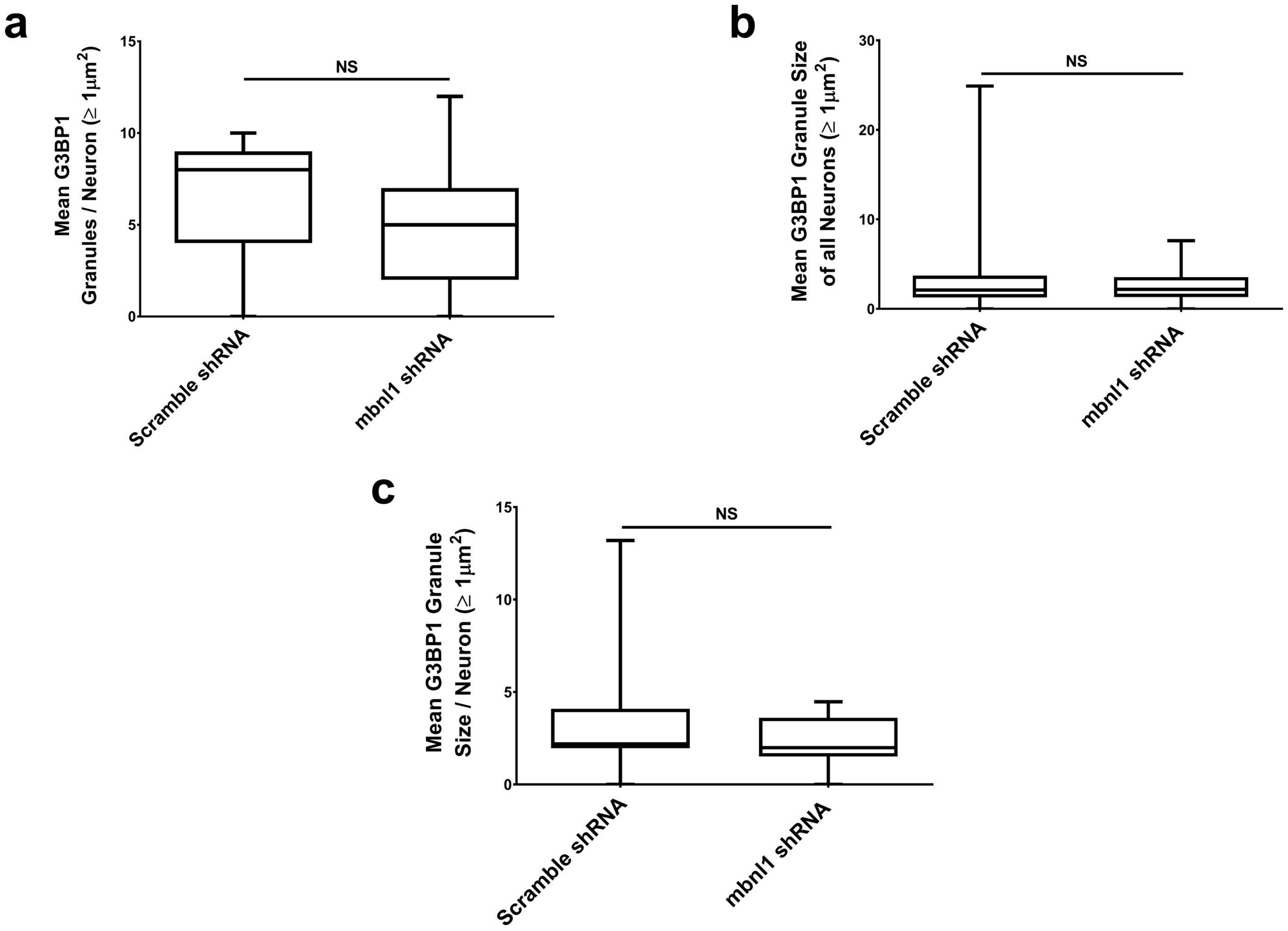

Supplementary Figure 15

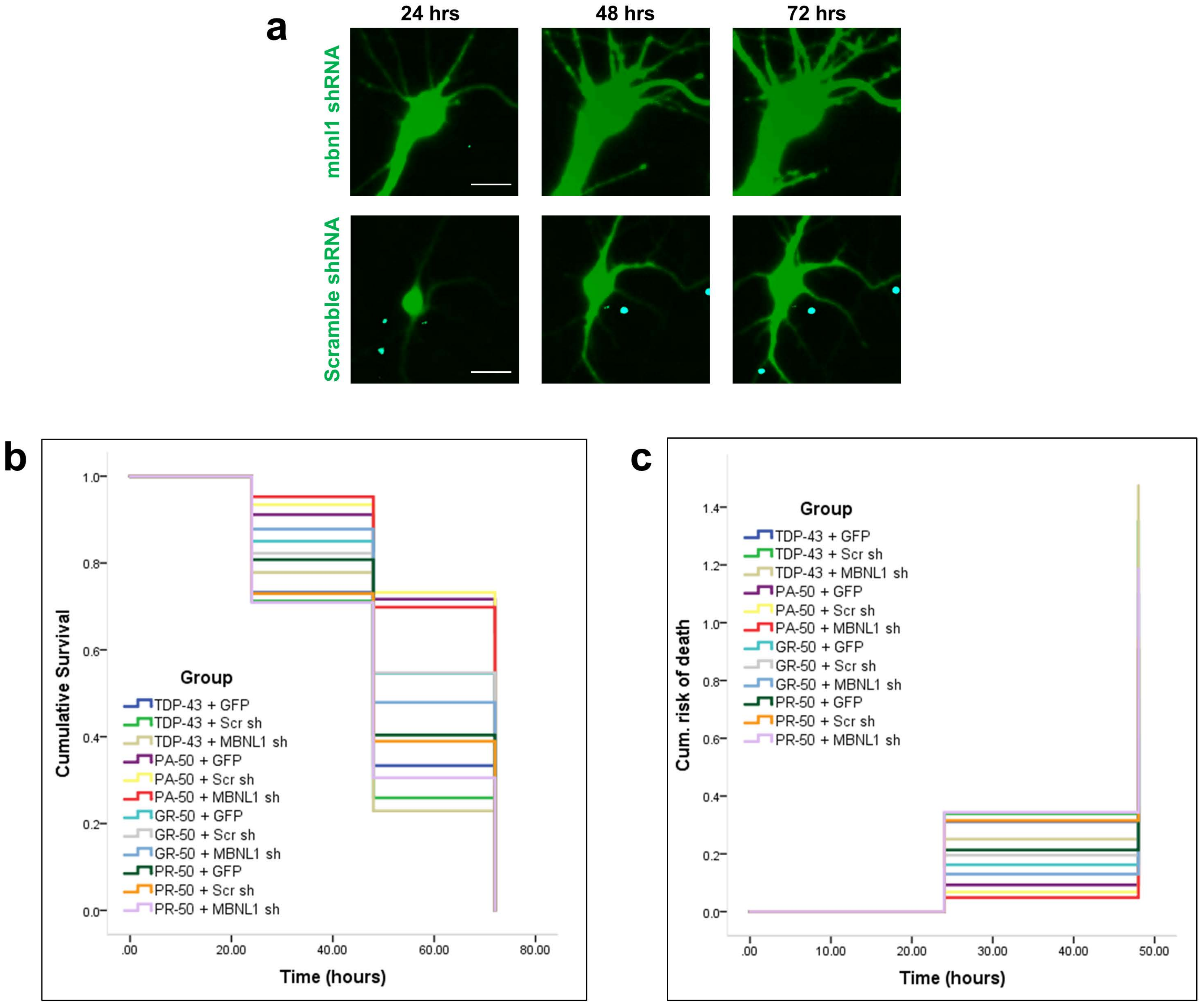

Supplementary Fig. 16

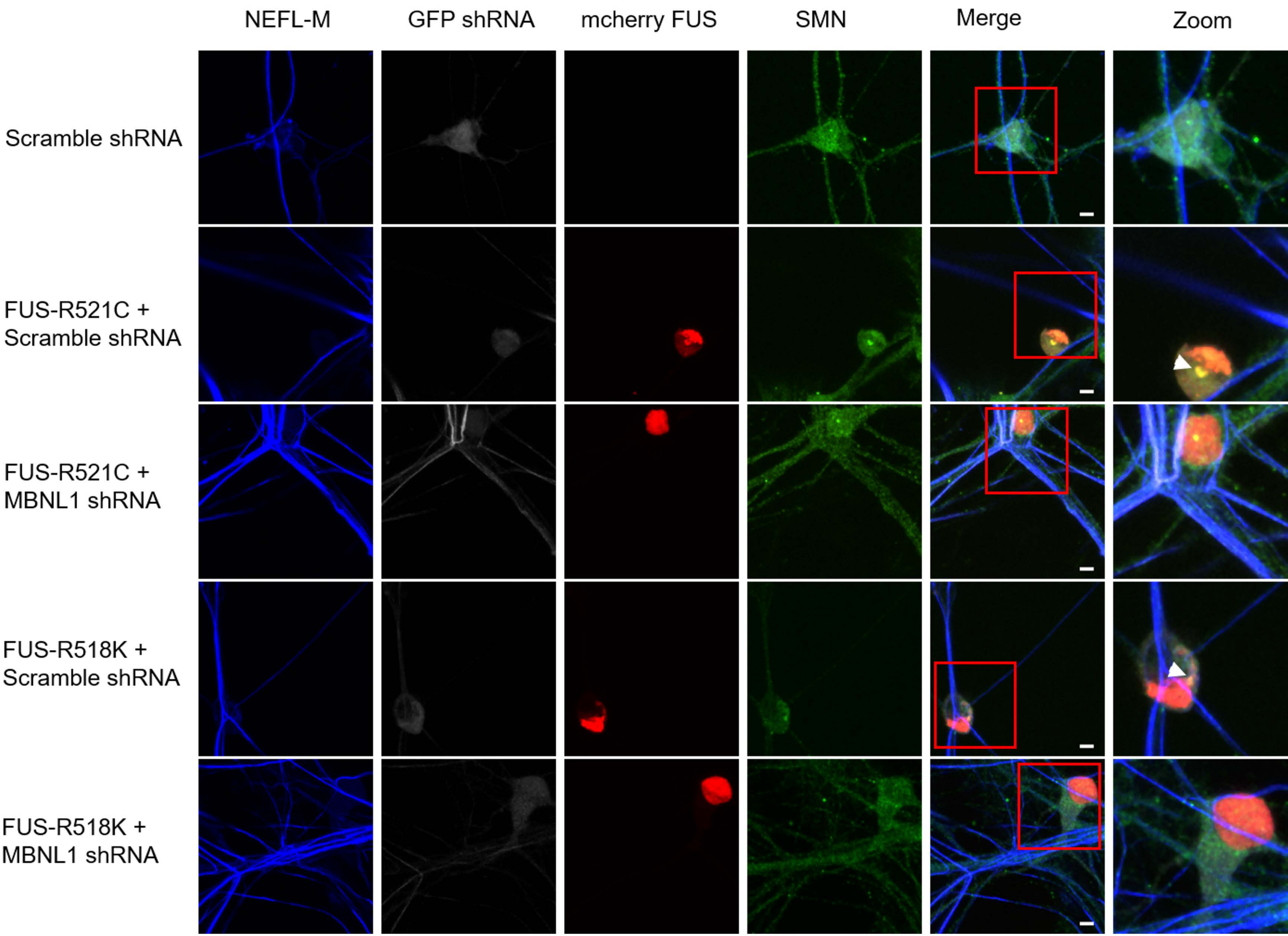

Supplementary figure 17

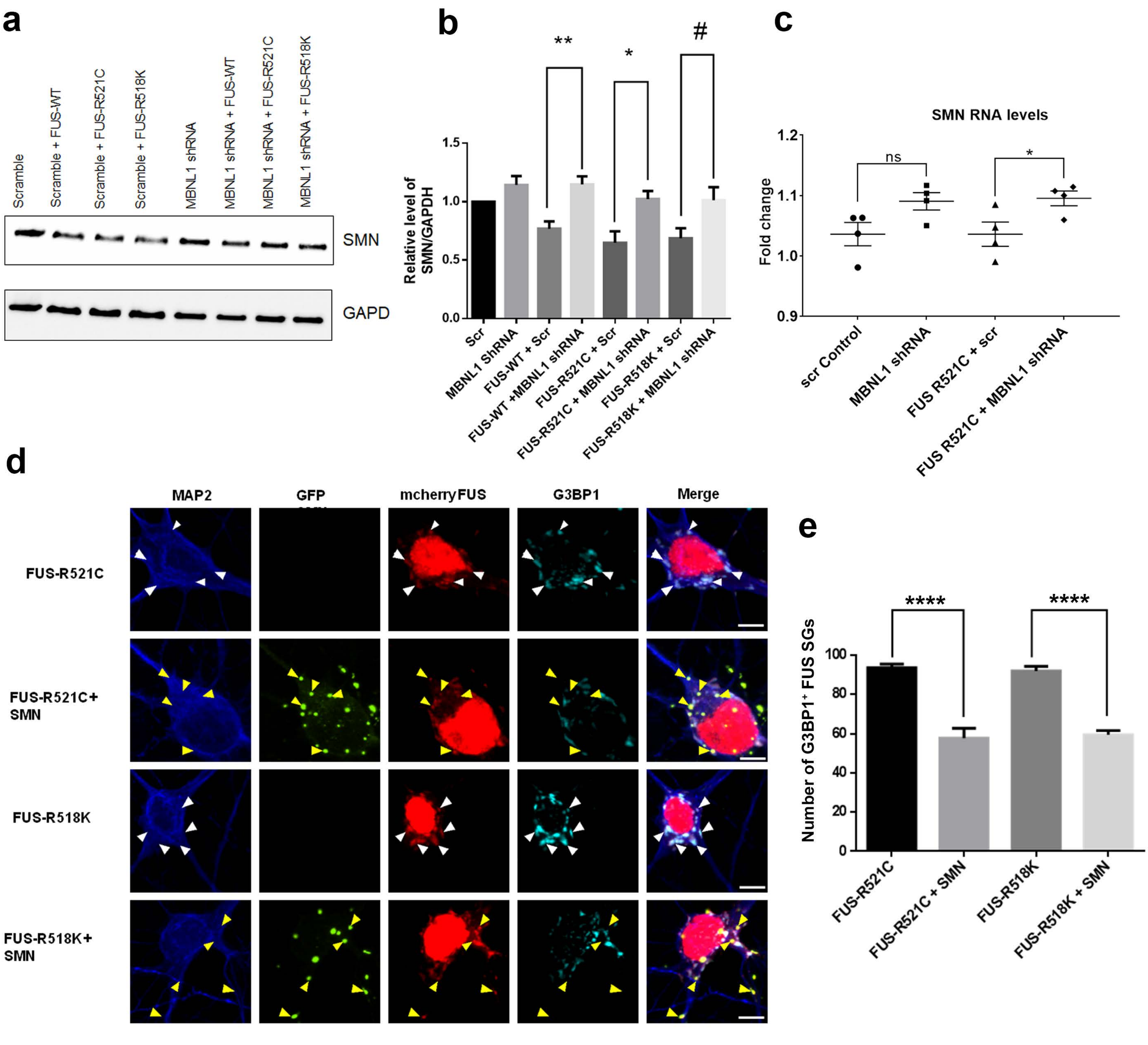

Supplementary Fig. 18

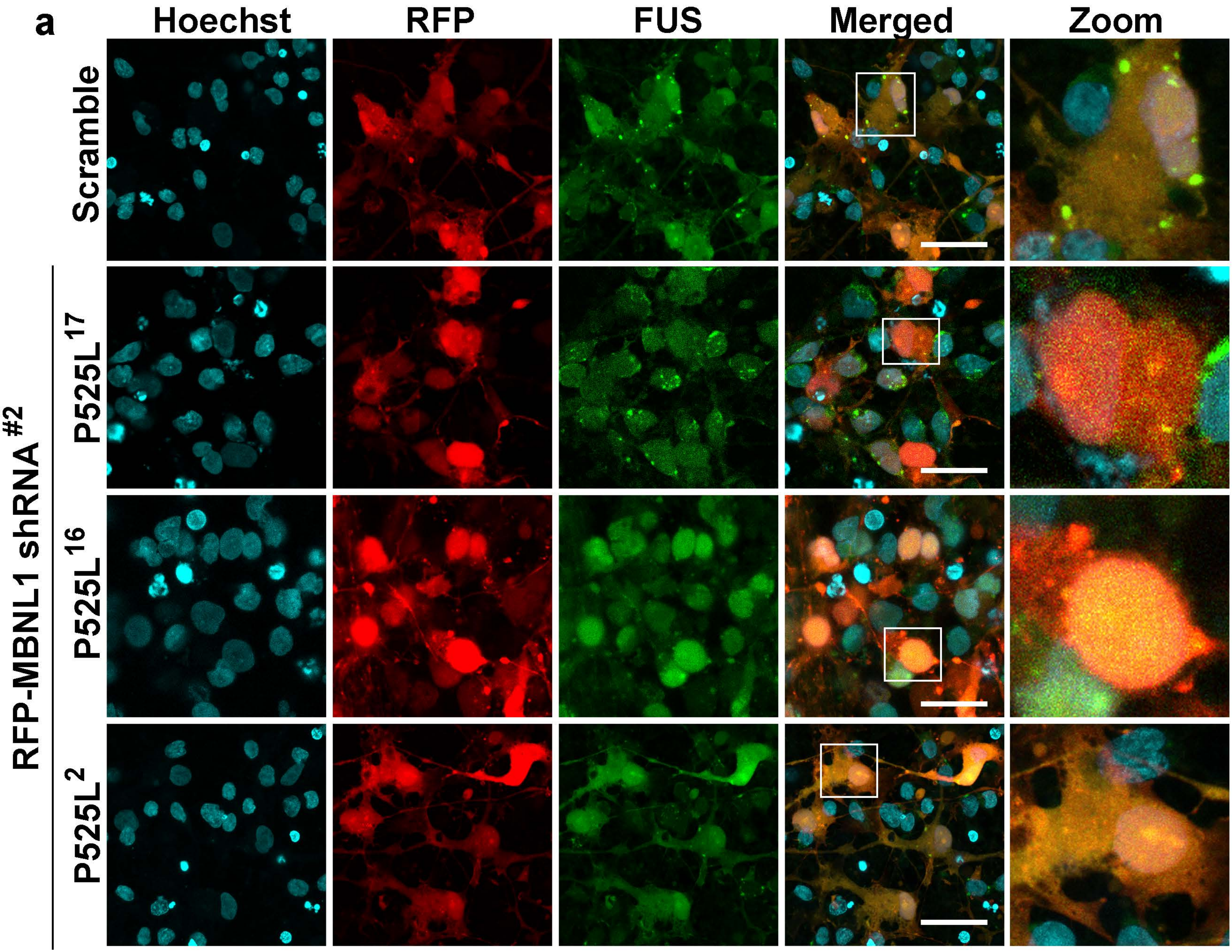

Supplementary Fig. 19

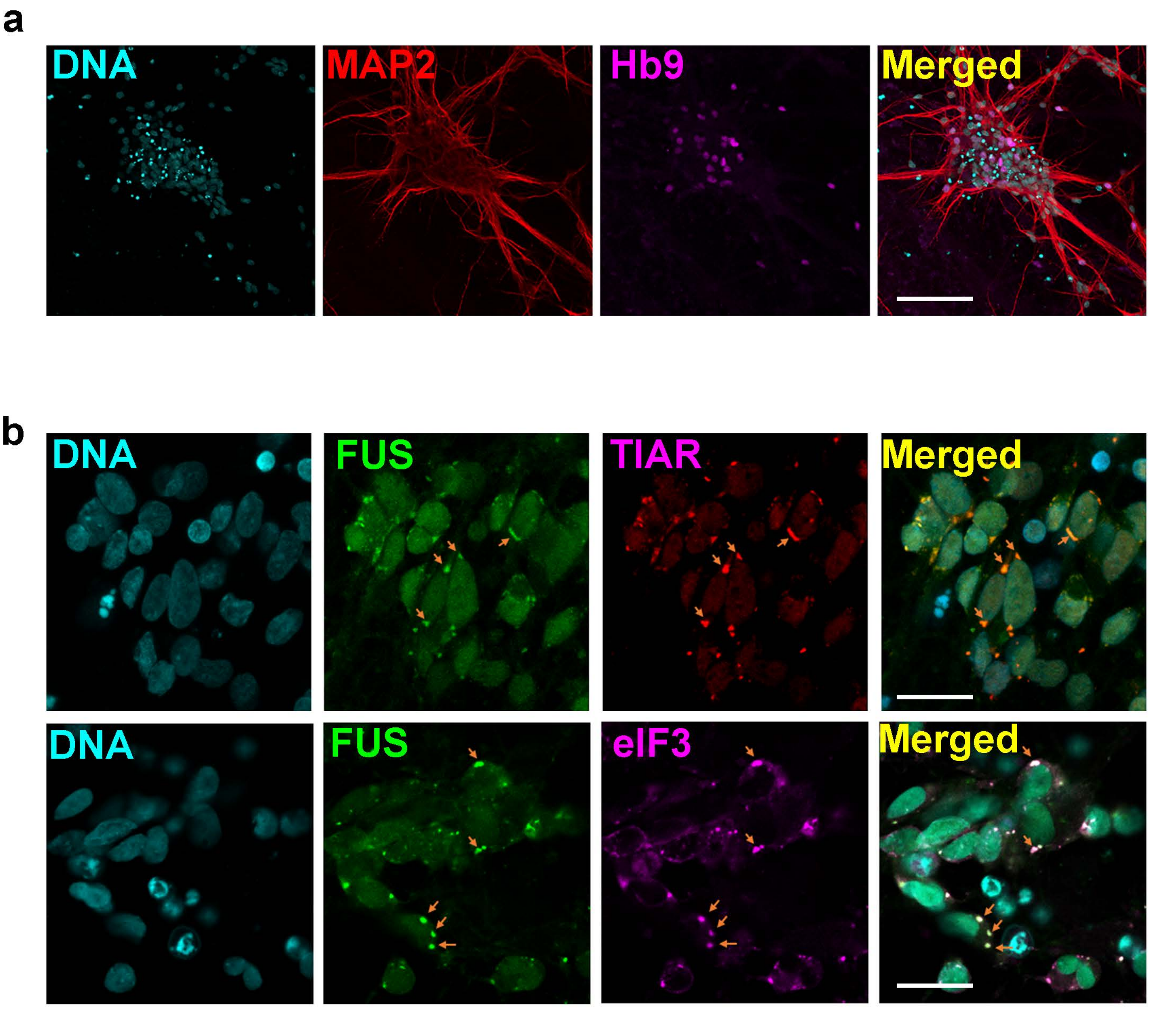

Supplementary Figure 1. Genetic screen performed on a *Drosophila* model of FUS-associated ALS reveals modifiers of FUS toxicity. (a) Flies from the DrosoDel deficiency kit (Bloomington *Drosophila* Stock Center) were crossed with flies expressing human, mutant FUS R521H. Progeny were assessed for changes in eye degeneration associated with mutant FUS expression were assessed in the progeny; deficiency lines showing enhancement or suppression of this phenotype were flagged for further study. The labels indicate the *Drosophila* lines provided in the kit. **(b)** Targeted knockdown of endogenous Mbl recapitulates the suppression of FUS toxicity observed in the Df(2R)Exel6066 deficiency line. Two independent *Drosophila* RNAi lines obtained from the Vienna *Drosophila* Resource Center (105486KK and 28732GD) were crossed with *Drosophila* expressing human, mutant FUS R521H in their eyes. Both RNAi lines confirmed that mbl reduction recapitulates the suppression of external eye degeneration observed in *Drosophila* that are heterozygous for the Df(2R)Exel6066 region. N=50-100 *Drosophila* per group.

Supplementary Figure 2. Altering mbl expression levels significantly affects eye degeneration in *Drosophila* expressing either wild-type or mutant FUS. *Drosophila* expressing either wild-type or mutant FUS with an eye-specific, GMR-gal4 driver were crossed with either *mbl* RNAi or *Drosophila* that overexpress *mbl*. Both external and internal eye degeneration were monitored in the progeny. **(a)** Representative *Drosophila* eyes showing suppression of FUS-associated toxicity following targeted knockdown of endogenous Mbl (N>50 flies per group). **(b)** Quantification of eye degeneration severity for each group. Kruskal-Wallis ANOVA with Dunn's multiple comparisons test was used to determine significance (****P=0.0001). The values are represented as the mean ± SD. **(c)** Representative, cross-sectional images of adult *Drosophila* eyes showing the level of sub-surface deterioration in Day 1 progeny. Changes in subsurface morphology are observed in wild-type or mutant FUS-expressing *Drosophila* following depletion (*mbl* RNAi) or augmentation (mblC OE) of Mbl levels.

Mbl expression levels affected the length and degradation of retinula cells (double arrows), as well as their separation from the basal lamina (single arrows).

Supplementary Figure 3. RNAi-mediated knockdown of genes within the Df(2R)Exel6066 deficiency region, other than *mbl*, does not suppress external eye degeneration caused by FUS R518K overexpression. *Drosophila* eyes expressing human FUS under the GMR-gal4 driver were crossed with RNAi lines to target genes, other than *mbl* in the Df(2R)Exel6066 deficiency region. **(a)** Representative panel of adult *Drosophila* eyes showing similar degeneration caused by expression of FUS R518K alone (top image) or in combination with RNAi-mediated depletion of the indicated proteins (bottom two rows). N>20 *Drosophila* per group. **(b and c)** Targeted knockdown of *CG12699*, a gene that overlaps *mbl* in the Df(2R)Exel6066 deficiency region, does not suppress external eye degeneration caused by overexpression of wild-type or mutant FUS. **(b)** Representative images of adult *Drosophila* eyes showing degeneration caused by tissue-specific expression of wild-type and mutant FUS with and without concurrent RNAi-mediated depletion of endogenous CG12699 (N>50 *Drosophila* per group). **(c)** Quantification of the eye degeneration shown in panel (a) indicates no significant differences in FUS-associated external eye degeneration with knockdown of endogenous CG12699. Kruskal-Wallis ANOVA with Dunn's multiple comparison's test was performed (NS=not significant). Values are represented as the mean ± SD.

Supplementary Figure 4. Knockdown of endogenous mbl suppresses FUS-associated degeneration over time. We measured eye degeneration caused by overexpression of wild-type and mutant FUS over time with and without concurrent depletion of endogenous mbl. **(a)** Representative images of *Drosophila* eyes showing degeneration caused by wild-type or mutant FUS expression at three different time points. The top row is the driver-alone control group, with no FUS expression. **(b)** Quantification of eye degeneration indicates a significant increase for all

FUS-expressing groups by day 10. **(c)** Representative images of *Drosophila* eyes showing degeneration with both FUS expression and RNAi-mediated depletion of endogenous mbl. The top row is the control group that is expressing only mbl RNAi. **(d)** Quantification of eye degeneration shows suppression of the age-related degeneration measured in panel (b) following mbl knockdown. Kruskal-Wallis ANOVA with Dunn's multiple comparisons test was used to determine statistical significance for each group in (b) and (d) (****P=0.0001, **P=0.0053, NS=not significant). Values are represented as the mean ± SD.

Supplementary Figure 5. FUS interaction with MBNL1 is not RNA-dependent and arginine methylation-dependent. **(a)** Representative IF images showing the effect of RNase treatment on the cytoplasmic co-localization of mutant FUS and MBNL1 in HEK cells. The WT and mutant FUS-transfected HEK cells were stained with anti MBNL 1 and anti-HA and the images were captured at 60X. The RNA depletion does not influence the sequestration of MBNL1 in the cytoplasmic mutant FUS puncta. **(b)** IP showing that FUS interaction with MBNL1 is direct and does not require RNA. The protein lysates from HEK293T cells transfected with HA-FUS, FUS-R518K and FUS-R521C were pull down by anti-HA with or without RNase and probed against MBNL1. **(c-d)** Overexpression of MBNL1 did not perturb FUS expression. HEK293T cells expressing endogenous or HA-tagged FUS-WT and indicated mutants were co-transfected with **(c)** vector expressing MBNL1-GFP and **(d)** sh-RNA against MBNL1 (or scramble) and treated with vehicle and Adox for 24hr. Cells were then processed for Western blot analysis (WB) with anti-FUS, anti-Tubulin, and anti-Calnexin antibodies. (c) HEK293T cells were transfected with MBNL1-EGFP or sh-MBNL1. Mock and scramble were used as controls, respectively. Cells were treated with vehicle or Adox (10 mM) for 24 hours and then subjected to nuclear/cytoplasmic fractionation. Endogenous FUS was analyzed in the nuclear and cytosolic fractions by Western blotting. Lamin B1 and alpha-tubulin were used as loading controls of nuclear and cytosolic fractions, respectively

Supplementary Figure 6. Endogenous muscleblind levels are unaffected by ALS-causing FUS mutations or by knockdown of endogenous Caz in *Drosophila*. qRT-PCR was performed on *Drosophila* and human samples to determine whether ALS-causing mutations of FUS or knockdown of Caz, the endogenous, *Drosophila* ortholog of FUS, would affect *mbl* RNA levels. **(a-c)** Graphs showing *mbl* RNA levels were not significantly different between groups. Reactions were performed in triplicate with biological replicates from each group listed in the graphs. Values are represented as the mean ± SD. **(a)** Endogenous *mbl* RNA levels in *Drosophila* eyes expressing wild-type and mutant FUS compared to controls. Only FUS R518K showed a minor increase in *mbl* RNA (*P=0.0133). **(b)** Endogenous muscleblind-like (MBNL1) RNA levels in two ALS patient lymphoblastoid cell lines expressing the indicated FUS mutants (ND14136 expressing FUS-R518G and ND14790 FUS R521C; Coriell Institute) show no significant changes compared to the two age and sex-matched controls (NS=not significant). Cell lines were analyzed at the third passage. **(c)** RNAi-mediated knockdown of endogenous Caz has no effect on mbl levels in *Drosophila*. **(d)** Endogenous MBNL1 protein levels in lymphoblastoid cell lines described in panel (b). Statistical analyses in panels (a), (b) and (d) were performed using one-way ANOVA with Tukey's multiple comparisons test. A two-tailed t-test was used in panel **(c).**

Supplementary Figure 7. FUS-associated toxicity is not suppressed by gal4 dilution. *Drosophila* expressing either wild-type or mutant FUS (FUS R518K and FUS R521C) in their eyes were crossed with *Drosophila* expressing GFP or luciferase. **(a)** Representative images of adult *Drosophila* eyes. The degeneration caused by tissue-specific expression of wild-type and mutant FUS (first column) is not suppressed by overexpression of GFP (second column) or luciferase (third and fourth columns). *Drosophila* with UAS-Luc express the protein using the VALIUM1 vector (Bloomington #35789). Luc Val10 flies express the protein using a

VALIUM10/VALIUM20 control vector (Bloomington #35788). **(b)** Quantification of FUS-associated external eye degeneration indicates no significant differences in FUS toxicity between groups (N>30 *Drosophila* per group). Values are represented as the mean ± SD. Kruskal-Wallis ANOVA with Dunn's multiple comparison's tests were performed for each comparison (N=Not significant).

Supplementary Figure 8. Targeted knockdown of endogenous mbl does not suppress the external eye degeneration caused by overexpression of other ALS-linked proteins in *Drosophila*. *Drosophila* overexpressing wild-type and ALS-linked mutant proteins in their eyes were crossed with *mbl* RNAi-expressing *Drosophila* under the GMR-gal4 driver. **(a)** Representative images of adult *Drosophila* eyes showing degeneration caused by tissue-specific expression of wild-type and mutant EWSR1, TDP-43, dVCP and C9orf72. Wild-type and mutant EWSR1, TDP-43 and C9orf72-30R images shown are from *Drosophila* overexpressing human orthologues of these proteins. Wild-type and mutant dVCP images shown are from *Drosophila* overexpressing the *Drosophila* ortholog of VCP. The dVCP R152H mutant is equivalent to the human ALS-associated VCP mutant. **(b)** Quantification of external eye degeneration of the groups shown in panel (a) confirms that there are no significant differences in ALS-linked protein toxicity when endogenous mbl levels are reduced, with the exception of wild-type EWSR1 which is a member of FET family protein similar to FUS. Values are represented as the mean ± SD. N>20 *Drosophila* per group. Mann-Whitney tests were performed for each comparison (N= Not significant, ****P=0.0001).

Supplementary Figure 9. Muscleblind-like protein 1 (MBNL1) accumulates into G3BP1-positive stress granules (SGs) in HEK293T cells and primary cortical neurons following exposure to stress. (a) Representative panel of HEK293T cells visualized by confocal microscopy after cultures were treated with the indicated stresses. G3BP1 was used as the marker for cytoplasmic SG formation. Cells exposed to all three stresses show colocalization of

MBNL1 with G3BP1-positive foci, confirming that MBNL1 accumulates in cytoplasmic SGs. Magnified images of the areas indicated with white boxes highlight cellular distributions of MBNL1 and colocalization with G3BP1. Scale bars=10 μm (scale bars=5 μm in the magnified images). **(b)** Representative IF panel showing FUS and G3BP1 localization in HEK293T cells under stress**. (c)** Cytoplasmic granules of MBNL1 and mutant FUS co-localize with the stress granule marker G3BP1. Cortical neurons were either transfected with FUS-R521C or FUS-R518K and 48 hours later stressed using sodium arsenite (0.5 mM, 90 minutes at 37C). Cells were then labeled with the neuronal marker MAP2 and stress granule marker G3BP1. Representative confocal microscope images of MAP2 (blue) positive cortical neurons show strong co-localization (white puncta in merged image) of cytoplasmic granules of endogenous MBNL (green), FUS-521C or FUS-R518K (red) and G3BP1 (gray). Scale bars = 5 μm

Supplementary Figure 10. ALS-associated mutant FUS, but not wild-type FUS, mislocalizes to the cytoplasm and incorporates with endogenous MBNL1 protein into cytoplasmic stress granules. Representative panel of HEK293T cells overexpressing FUS (wild-type, R518K, or R521C) and visualized by confocal microscopy. Cells were imaged in the presence and absence of sodium arsenite-induced stress. Cells were probed for HA-tagged, exogenous FUS and endogenous MBNL1. Wild-type FUS primarily localized to nuclei in both stressed and unstressed cells. Both R518K and R521C mutant FUS mislocalized to the cytoplasm. Cells with cytoplasmic FUS develop FUS and MBNL1-positive, cytoplasmic, SGs in both the presence and absence of sodium arsenite-induced stress. Nuclei were stained with DAPI. Magnified images highlight the cellular distributions of MBNL1 and FUS, and the formation of SGs. Scale bars=20μm (scale bars in magnified images=5μm).

Supplementary Figure 11. HEK293T and N2A cells transiently transfected with muscleblind (*mbnl1*)-specific shRNA show significant reductions of muscleblind protein.

(a and b) HEK293T cells were transfected with MBNL1-specific shRNA. **(a)** Representative Western blot of endogenous MBNL1 protein following transient transfection with either a scramble shRNA or MBNL1 shRNA. Tubulin was used as a loading control. **(b)** Quantification of MBNL1 signal normalized to tubulin from transfected HEK293T cells. MBNL1 protein is significantly reduced in shRNA-treated cells compared to control cells. **(c and d)** Mouse neuro2a (N2A) cells were transfected with *mbnl1*-specific shRNAs. **(c)** Representative Western blot detecting endogenous mbnl1 protein following transfection of scrambles or one of four *mbnl1*-specific shRNAs. Untransfected cells were used as a control. Tubulin was used as a control. **(d)** Quantification of mbnl1 signal normalized to tubulin from transfected N2A cells. Two of the four shRNAs showed significant knockdown of mbnl1 shRNA. Untransfected cells and those transfected with the scrambled shRNA were used as control groups to determine the effectiveness of the mbnl1-specific shRNAs. shRNA D was selected for use in subsequent experiments in rat cortical neurons. For all muscleblind Western blots, both MBNL1 bands of the characteristic doublet were used in the quantification (N=3 biological replicates). Values in (b) and (d) are represented as the mean ± SD. Quantifications were performed using Image Studio (Li-Cor), and statistical analyses were performed with Prism 6 (GraphPad). Statistical significance in (b) was determined by a two-tailed t-test (*P=0.0468). Statistical significance in (d) was determined using one-way ANOVA with Dunnet's multiple comparison test (**P<0.01, ***P<0.001, NS=not significant).

Supplementary Figure 12. shRNA-mediated knockdown of endogenous MBNL1 reduces mutant FUS-positive cytoplasmic stress granules (SGs) in the absence of external cellular stress. HEK293T cells were co-transfected with FUS (wild-type, R518K, or R521C) and either MBNL1 shRNA or scramble shRNA. Cellular distribution of FUS and MBNL1 as well as the presence of SGs were assessed by confocal microscopy. **(a-c)** Representative confocal

images showing FUS and MBNL1 distributions in HEK293T cells. Cell nuclei were stained with DAPI. White traces in the MBNL1 column highlight representative MBNL1 shRNA-transfected cells with reduced MBNL1 signal. White boxes indicate the areas presented in the magnified images. Scale bars = 10μm (scale bars = 5μm in the magnified images). In the absence of external stress, FUS-positive SGs (white arrows) form in the cytoplasms of mutant FUS-expressing cells, but not in wild-type-expressing cells. Depletion of endogenous MBNL1 reduces FUS-positive SG formation. **(d-f)** Quantification of the percentage of cells with FUS-positive SGs for each group. Graphs correspond to the representative images in above panels (a, b or c). N> 50 cells per condition. Statistical significances were determined using two-tailed t-tests (*P=0.0191, **P=0.0035, NS=not significant).

Supplementary Figure 13. Cellular distributions of MBNL1, shRNA and FUS in transiently transfected rat cortical neurons. (a) Representative fluorescence images of primary cortical neurons transfected with either scrambled or *Mbnl1* shRNA. Cells are stained for MBNL1 protein and MAP-2, a neuronal cell marker. mbnl1 levels are reduced in *Mbnl1* shRNA-transfected cells compared to scrambled shRNA controls. **(b and c)** Representative fluorescence images showing the cellular distributions of transiently transfected FUS and shRNAs. Nuclei are stained with DAPI to identify nuclear and cytoplasmic cellular compartments. MAP-2 is used as a marker for neuronal cells. **(b)** Scrambled and *Mbnl1* shRNA are distributed throughout both the nucleus and the cytoplasm. **(c)** Similar to HEK293T cells, wild-type FUS primarily localizes to the nucleus, but ALS-linked mutant FUS (R521C) mislocalizes to the cytoplasm. White boxes indicate the magnified areas.

Supplementary Figure 14. Quantifications of SG size and number in rat cortical neurons expressing scrambled or *Mbnl1*-specific shRNA. Neurons positive for shRNA were analyzed for SG number and size. Only SGs greater than or equal to 1μm^2 were used in the

quantifications. No significant differences are observed in the **(a)** average number of G3BP1-positive SGs/neuron, **(b)** average size of total SGs across all neurons, **(c)** or average size of SGs/neuron between scrambled and *mbnl1* shRNA-expressing neurons. Values are represented as the means ± SEM. N=10-15 cells per group. Two-tailed t-tests were performed for each comparison (NS = not significant).

Supplementary Figure 15. shRNA-mediated knockdown of endogenous mbnl1 does not suppress TDP-43-associated toxicity or C9-DPRs (dipeptide repeats). (a) Representative fluorescence images indicating expression of both mbnl1-specific and scramble shRNA over the course of 72 h in cortical neurons. DRAQ7 staining within cells (cyan color) is a marker for cell death; the lack of staining throughout the time course indicates that *mbnl1* and scrambled shRNAs are nontoxic to these cells. **(b)** Kaplan-Meier cumulative survival curves. Depletion of endogenous mbnl1 does not suppress TDP-43 or DPR-associated toxicity (N>100 cells per group pooled from three independent experiments). Graph was generated with SPSS software. Log-rank with Mantel-Cox tests were used for comparisons. Statistical comparisons were not significant. **(c)** Cumulative risk of death curves that correspond to the survival analysis.

Supplementary figure 16: MBNL1 knockdown prevents cytoplasmic SMN puncta and mislocatization in motor neurons. Primary rat motor neurons were co-transfected with mCherry tagged mutant FUS constructs (FUS-R518K or FUS-R521C) and either GFP tagged MBNL1 shRNA or scramble shRNA. 48 hours later, transfected cells were labeled for SMN and the axonal marker NEFL-M by immunocytochemistry and observed using confocal microscopy. Representative confocal images showing SMN and FUS distribution in neuronal cells. NEFL-M staining was used to trace axons. Labels on the left side of the panel detail which groups were transfected with MBNL1 shRNA or scramble shRNA. White arrows in the zoom panel indicate co-localization of mutant FUS and SMN puncta. Scale bars = 5μm.

Supplementary figure 17: Knockdown of endogenous muscleblind upregulates SMN protein and RNA levels and SMN overexpression reduces incorporation of mutant FUS into cytoplasmic stress granules: (a) NSC-34 cells were co-transfected with FUS (either wildtype, R521C or R518K) and either Mbnl1-specific or scrambled shRNA. Representative Western blot of NSC-34 cells probed for SMN. GAPDH was used as a loading control.
(b) Quantification of SMN levels in NSC-34 cells (N=6). **(c)** HEK293T cells were transfected with scrambled shRNA, MBNL1 shRNA, FUS R521C and FUS R521C + MBNL1 shRNA and qPCR was performed to determine the SMN RNA levels. We observed that SMN RNA levels was increased upon MBN1 KD in FUS R521C group as compared to scrambled controls. **(d)** SMN expression reduces incorporation of mutant FUS into cytoplasmic stress granules in cortical neurons. Cortical neurons were transfected with FUS-R521C and FUS-R518K alone or in combination with SMN. Cells were then stressed using sodium arsenite (0.5 mM, 90 minutes at 37C) and labeled for the stress granule marker G3BP1. Representative confocal microscope images of cortical neurons labeled with MAP2 (blue) and G3BP1 (cyan) in mCherry tagged mutant FUS-R521C and FUS-R518K (red) alone or with GFP tagged SMN (green). Mutant FUS when expressed alone co-localizes strongly with G3BP1 (co-localized white puncta shown by white arrows). Co-expression of mutant FUS with SMN reduces co-localization of mutant FUS granules with G3BP1. Exogenously expressed SMN strongly co-localizes with cytoplasmic mutant FUS granules (co-localized yellow puncta shown by yellow arrows) and are not positive for G3BP1. **(e)** Quantification of the percentage of cytoplasmic FUS granules associated with G3BP1. Only stress granules equal to, or greater than, 1μm^2 were used in the quantifications. A minimum of 10-15 cells were analyzed from each group from 3 independent experiments. Values presented are means ± SEM. The average number of G3BP1-positive FUS granules is significantly lower in SMN co-expressing cells neurons compared to mutant FUS alone expressing cells. All statistical analyses were performed on GraphPad Prism software. One way ANOVA were used for the comparisons in b (****P ≤ 0.0001).

Supplementary figure 18: MBNL1 knockdown reduces FUS positive stress granules in iPSC-derived neurons. (**a)** Representative IF image representing FUS positive stress granules in control and FUS P525L iPSC-derived motor neurons following sodium arsenite treatment. (**b)** Quantification of the percentage of iPSC-derived motor neurons showing FUS positive stress granules following lentiviral delivery of MBNL1 shRNA (construct 2) and scramble control (n=50-60 neurons/line). Statistically significant differences were determined by two-tail, unpaired t-test in B and two-way ANOVA followed by a Tukey post hoc analysis in C: ** $p \leq 0.01$ are indicated.

Supplementary figure 19: FUS P525L iPSC-derived motor neurons stain positive for anti-Hb9 and anti-TIAR (stress granule maker): (a) Image representing Hb9 staining (motor neuron marker) in FUS P525L indicating successful differentiation of iPSCs into mixed population of cells with 50% motor neurons. **(b)** Mutant FUS incorporates into the cytoplasmic stress granules as evident from anti-TIAR and anti-eIF3 (stress granule markers) staining. We used three different isogenic clones of FUS P525L iPSC line and these isogenic clones are labeled as 2, 16 and 17.

Supplementary Table 1. ***Drosophila*** **lines, site of insertion and their source used in the manuscript.**

Strains	Source	Reference
UAS-FUS WT (site-specific)	Pandey lab	Current paper
UAS-FUS R518K (site-specific)	Pandey lab	Current paper
UAS-FUS R521C (site-specific)	Pandey lab	Current paper
UAS-FUS R521H (site-specific)	Pandey lab	Current paper
UAS-FUS P525L (site-specific)	Pandey lab	Current paper
UAS-mbl RNAi KK (site-specific)	Vienna Drosophila stock center	Stock #105486
UAS-mbl RNAi GD (random)	Vienna Drosophila stock center	Stock #28732
UAS-FLAG-GFP (site-specific)	Pandey lab	Wen et al., 2014
UAS-Luciferase (site-specific)	Bloomington stock center	Stock #35788
UAS-Luciferase val10 (site-specific)	Bloomington stock center	Stock #35789
UAS-mbl Overexpression (random)	Provided by Dr. Ruben Artero	Garcia-Casado et al., 2002
UAS-EWSR1 (random)	Provided by Dr. Nancy Bonini	Couthouis et al., 2012
UAS-EWSR1 P552L (random)	Provided by Dr. Nancy Bonini	Couthouis et al., 2012
UAS-TDP-43 WT (random)	Provided by Dr. J Paul Taylor	Ritson et al., 2010
UAS-TDP-43 M337V (random)	Provided by Dr. J Paul Taylor	Ritson et al., 2010
UAS-VCP WT (random)	Provided by Dr. J Paul Taylor	Ritson et al., 2010
UAS-VCP R152H (random)	Provided by Dr. J Paul Taylor	Ritson et al., 2010
UAS-C9orf72 30R (random)	Provided by Dr. Peng Jin	
UAS-FUS WT (random)	Pandey lab	Lanson et al., 2011
UAS- FUS R521H (random)	Pandey lab	Lanson et al., 2011
Elav-GS	Provided by Dr. Haig Keshishian	Lanson et al., 2011
OK371-gal4	Bloomington stock center	Stock#26160
W1118	Bloomington stock center	Stock# 5905
GMR-ga4	Bloomington stock center	Stock#1104
UAS-Smn RNAi	Bloomington stock center	Stock #36621
UAS-Smn Overexpression	A kind gift from Dr. Spyros Artavanis-Tsakonas	Chang et al., 2008

Supplementary Table 2. Deficiency lines identified in our screen as modifiers of mutant FUS toxicity.

Deficiency Line	Bloomington Stock #	Enhance (E) / Suppress (S)	Chromosome
Df(1)Exel7468	7768	**S**	1
Df(1)ED6584	9348	**S**	1
Df(1)ED6712	9169	**S**	1
Df(2R)Exel6066	7548	**S**	2
Df(2L)BSC688	26540	**S**	2
Df(2L)BSC50	8469	**S**	2
Df(2L)ED4651	8904	**S**	2
Df(2L)Exel6012	7498	**S**	2
Df(2L)BSC180	9610	**S**	2
Df(2L)ED7853	24124	**S**	2
Df(2L)BSC209	7739	**S**	2
Df(3R)Exel6201	7680	**S**	3
Df(3R)BSC568	25126	**S**	3
Df(3R)Exel6272	7739	**S**	3
Df(3R)BSC137	9497	**S**	3
Df(3L)Exel8104	7929	**S**	3
Df(1)BSC834	27886	**E**	1
Df(1)BSC772	26869	**E**	1
Df(1)FDD-0024486	23295	**E**	1
Df(2L)BSC768	26865	**E**	2
Df(2L)BSC244	9718	**E**	2
Df(2R)M41A10	741	**E**	2
Df(2R)Exel6064	7546	**E**	2
Df(3R)ED10845	9487	**E**	3

Supplementary Table 3. ***Drosophila*** **genes located within the genomic region spanned by the Df(2R)Exel6066 deficiency line.**

Gene	VDRC RNAi Lines Available?	Site-Specific Integration RNAi Lines?
CG30456	Yes	Yes
CG15611	Yes	Yes
Amy-p	Yes	No
CR45272	No	No
Spn53F	Yes	Yes
Amy-d	Yes	Yes
CG15605	Yes	Yes
Cda9	Yes	No
CR45273	No	No
Acp54A1	Yes	Yes
CG11400	Yes	Yes
CR44391	No	No
Gbp1	Yes	Yes
Gbp2	Yes	No
CG43103	No	No
CG43107	No	No
CR44386	No	No
snoRNA:U3:54Aa	No	No
tRNA:Leu-AAG-1-2	No	No
tRNA:Leu-AAG-1-3	No	No
snoRNA:U3:54Ab	Yes	No
CG17290	Yes	Yes
CG17287	Yes	No
CG30458	Yes	No
CG30457	Yes	No
CG10953	Yes	No
CR44387	No	No
CG44388	No	No
CR44389	No	No
CR45323	Yes	No
CG10950	Yes	No
CG43237	No	No
CR46235	No	No
CG18469	Yes	Yes
CG12699	Yes	Yes
mbl	Yes	Yes
CR43660	No	No
CG43272	Yes	No
CG43108	No	No
CR45997	No	No
CR43661	No	No
CR44344	No	No
tRNA:Ala-TGC-1-1	No	No
tRNA:Ala-TGC-2-1	Yes	No
CG10939	Yes	Yes

Supplementary Table 4: Primers used in the manuscript.

Gene Name	Forward Primer (5'-3')	Reverse Primer (5'-3')	Probe (5'-3')
mbl (*Drosophila)*	ACTTGCATTCGGTGTCCTG	GTTGTCAATATGAACAGCCTGC	CAACGGCAAGGATTCGCGCT
FUS (Human)	CACAGACTCAATTGTAACATTCTCA	GGAGGCAGAGGTGGCAT	AGGCCTTGCACAAAGATGGTGTTG
alphatub84b (*Drosophilia*)	CCTCGAAATCGTAGCTCTACAC	ACCAGCCTGACCAACATG	TCACACGCGACAAGGAAAATTCACAGA
MBNL1 (Human)	TGCGTCCATTTATCTCCAACT	GAGTAATCGCCTGCTTTGATTC	TGCAAATATCTTCATCCACCCCCACA
GAPDH (Human)	TGTAGTTGAGGTCAATGAAGGG	ACATCGCTCAGACACCATG	AAGGTCGGAGTCAACGGATTTGGTC
