# Supplementary material for: Muscleblind acts as a modifier of FUS toxicity by modulating stress granule dynamics and SMN localization
Source: Nat Commun. 2019 Dec 6;10:5583. doi: 10.1038/s41467-019-13383-z (PMC6898697; doi:10.1038/s41467-019-13383-z)
Supplement: Supplementary file 3 — Reporting Summary [file 41467_2019_13383_MOESM3_ESM.pdf]

nature research

Corresponding author(s): Udai Pandey

Reporting Summary

Nature Research wishes to improve the reproducibility of the work that we publish. This form provides structure for consistency and transparency in reporting. For further information on Nature Research policies, see Authors & Referees and the Editorial Policy Checklist.

Statistical parameters

When statistical analyses are reported, confirm that the following items are present in the relevant location (e.g. figure legend, table legend, main text, or Methods section).

n/a	Confirmed	
☐	☒	The exact sample size (n) for each experimental group/condition, given as a discrete number and unit of measurement
☐	☒	An indication of whether measurements were taken from distinct samples or whether the same sample was measured repeatedly
☐	☒	The statistical test(s) used AND whether they are one- or two-sided<br>*Only common tests should be described solely by name; describe more complex techniques in the Methods section.*
☒	☐	A description of all covariates tested
☒	☐	A description of any assumptions or corrections, such as tests of normality and adjustment for multiple comparisons
☐	☒	A full description of the statistics including central tendency (e.g. means) or other basic estimates (e.g. regression coefficient) AND variation (e.g. standard deviation) or associated estimates of uncertainty (e.g. confidence intervals)
☒	☐	For null hypothesis testing, the test statistic (e.g. F, t, r) with confidence intervals, effect sizes, degrees of freedom and P value noted<br>*Give P values as exact values whenever suitable.*
☒	☐	For Bayesian analysis, information on the choice of priors and Markov chain Monte Carlo settings
☒	☐	For hierarchical and complex designs, identification of the appropriate level for tests and full reporting of outcomes
☐	☒	Estimates of effect sizes (e.g. Cohen's d, Pearson's r), indicating how they were calculated
☐	☒	Clearly defined error bars<br>*State explicitly what error bars represent (e.g. SD, SE, CI)*

Our web collection on statistics for biologists may be useful.

Software and code

Policy information about availability of computer code

Data collection	Not applicable
Data analysis	Not applicable

For manuscripts utilizing custom algorithms or software that are central to the research but not yet described in published literature, software must be made available to editors/reviewers upon request. We strongly encourage code deposition in a community repository (e.g. GitHub). See the Nature Research guidelines for submitting code & software for further information.

Data

Policy information about availability of data

All manuscripts must include a data availability statement. This statement should provide the following information, where applicable:

- Accession codes, unique identifiers, or web links for publicly available datasets
- A list of figures that have associated raw data
- A description of any restrictions on data availability

The datasets generated during and/or analysed during the current study are available from the corresponding author Udai Pandey on email or mail request

April 2018

Field-specific reporting

Please select the best fit for your research. If you are not sure, read the appropriate sections before making your selection.

- [x] Life sciences
- [ ] Behavioural & social sciences
- [ ] Ecological, evolutionary & environmental sciences

For a reference copy of the document with all sections, see nature.com/authors/policies/ReportingSummary-flat.pdf

Life sciences study design

All studies must disclose on these points even when the disclosure is negative.

Sample size	Sample sizes are justified in the figure legends.
Data exclusions	No data was excluded from the study presented in the manuscript.
Replication	Each experiment was performed 3-5 times and reported.
Randomization	No randomization was used for any of the experiments in this manuscript.
Blinding	No blinding was performed for any of the experiments in this manuscript.

Reporting for specific materials, systems and methods

Materials & experimental systems

n/a	Involved in the study	
x		Unique biological materials
	x	Antibodies
	x	Eukaryotic cell lines
x		Palaeontology
x		Animals and other organisms
x		Human research participants

Methods

n/a	Involved in the study	
x		ChIP-seq
x		Flow cytometry
x		MRI-based neuroimaging

Antibodies

Antibodies used	All antibody information is provided in the materials and methods section. All antibodies used have been previously reported. These previous studies are available in the References section.
Validation	All antibodies were obtained from companies that reported validation in the system under study.

Eukaryotic cell lines

Policy information about cell lines

Cell line source(s)	he HEK293T and Neuro-2A cells used were received fresh from ATCC and authenticated by their standard quality control procedures. Product numbers and a description of the passages used are provided in the materials and methods section
Authentication	The cell lines were directly purchased from the ATCC and only low passage
Mycoplasma contamination	The cell lines were negative for any mycoplasma contamination
Commonly misidentified lines (See ICLAC register)	not applicable.
