# Supplementary material for: Muscleblind acts as a modifier of FUS toxicity by modulating stress granule dynamics and SMN localization
Source: Nat Commun. 2019 Dec 6;10:5583. doi: 10.1038/s41467-019-13383-z (PMC6898697; doi:10.1038/s41467-019-13383-z)
Supplement: Supplementary file 2 — Peer Review File [file 41467_2019_13383_MOESM2_ESM.pdf]

Editorial Note: Parts of this peer review file have been redacted as indicated to remove third-party material where no permission to publish could be obtained.

Reviewers' comments:

Reviewer #1 (Remarks to the Author):

In this study, Casci et al. examine the basis for FUS neurotoxicity by seeking modifiers of toxicity in a fly model, uncovering the Drosophila orthologue of mammalian Muscleblind. The authors nicely document that decreased dosage of Drosophila Muscleblind can rescue relevant neuromuscular disease phenotypes in the FUS fly model. Importantly, Mbl dosage reduction does not modify other ALS disease-causing fly mutants, suggesting selectivity for the Muscleblind-FUS interaction.

The authors then extend their findings to mammalian cell lines, iPSC-derived FUS patient motor neurons, and rodent primary neurons, convincingly demonstrating that reduced muscleblind-like 1 (MBNL1) can reduce FUS incorporation into stress granules and will favor nuclear localization of FUS. The authors also address a previously reported connection between SMN suppression of FUS toxicity, showing that reduced MBNL1 can increase SMN RNA levels and SMN protein levels in axons, with beneficial effects on FUS toxicity.

Overall, this is an intriguing and carefully performed investigation, that unexpectedly yields evidence for MBNL1 as a potentiator of the FUS disease process, unlike its previously ascribed role as a suppressor of CUG expansion RNA toxicity through its modulation of splicing. Certainly the fact that MBNL1 modification is restricted to FUS is very unexpected, appears well supported, and a crucial piece of the story. The authors propose that MBNL1 enhances FUS toxicity by somehow promoting FUS association with stress granules, while at the same time, preventing SMN from engaging with FUS. A bit more clarity in the pathogenic events underlying how MBNL1 mediates these effects would further elevate the impact of this already highly important and meaningful study, which will be of great interest to the broader neurological disease field.

1) There is some uncertainty as to whether MBNL1 RNA is involved in the subcellular localization of FUS protein. Experiments to test the ability of mutant MBNL1 RNA lacking FUS binding sites might clarify the role of FUS – MBNL1 RNA interactions in regulating the stress granule association effect.

2) Another option might be to better understand the nature of the FUS – MBNL1 protein interaction. Is it direct or indirect? Can MBNL1 protein incapable of FUS interaction still promote FUS stress granule association?

3) I do not understand how MBNL1 prevents SMN RNA expression from properly occurring in the context of mutant FUS expression. Is this due to mutant FUS binding to SMN RNA and preventing its processing? Or does this reflect an enhanced directing of SMN RNA to stress granules preventing its transcription? Some additional insight into how MBNL1 affects FUS regulation of SMN RNAs would be helpful.

Reviewer #2 (Remarks to the Author):

In this manuscript, the authors report their identification of Drosophila muscleblind and its mammalian orthologue MBNL1 as a modifier of FUS toxicity. They conclude that that mechanism lies in modulating stress granule dynamics and restoring SMN levels via MBNL1 downregulation.
The identification of MBNL as a modifier of FUS toxicity is certainly a novel and interesting finding. This part of the manuscript is very thorough and uses model systems from Drosophila to mammalian neuronal and human iPSC-derived motor neurons to make a very strong and convincing case. However, the mechanistic link to SG formation and especially to SMN levels is far less clear and fleshed out as described in more detail below.
1) The term stress granules (SGs) is used to describe cytoplasmic foci, w/o demonstrating that they are positive for SG markers:
Generally, SGs are identified by the presence of key components, such as G3BP. In this study, cytoplasmic granules are described as SGs, without actually showing the co-localization with SG markers. The exception here is Suppl. Fig. 8, but most figures just show FUS or MBNL-positive puncta.

2) The question how MBNL levels modulate FUS toxicity and SG formation is not sufficiently explored: There are obvious possibilities that are not investigated in this study. We now know that FUS localization and aggregation/LLPS depends on its association with transportin 1 and by posttranslational arginine methylation of FUS (see 4 papers in Cell from April 2018). Does MBNL affect this posttranslational modification? It even seems that the lower FUS band is increased by mbl RNAi in Fig. 1h. It would be important to show whether this process that regulates FUS phase separation is affected by MBNL.

3) Does MBNL mitigate FUS toxicity by restoring SMN levels?
It is unclear what the authors mean with manipulating muscleblind levels to “restore reduced SMN protein levels in axons”. The same is true for the heading “SMN protein trapped in axons by pathogenic mutations in FUS is rescued by MBNL1 knockdown”. This appears to refer to a study published in HMG in 2013 “ALS-associated mutations in FUS disrupt the axonal distribution and function of SMN”. It is not clear what aggregates are axonal or cytoplasmic and what is rescued. Is SMN reduced in axons? Is SMN trapped in axons? It seems that SMN may be trapped in cytoplasmic inclusions as shown in Fig. 6, but the present study does not quantify SMN levels in axons. NEFL is not an axon-specific marker, unless it is specific for phospho-NF isoforms present only in axons. This is not clear from the methods, and the images do not show axon-specific staining. There is no quantitative WB analysis and the reduction of SMN protein levels appears minor and is not obvious from the ICC staining. The authors also describe that ectopic expression of SMN suppressed the external eye degenerative phenotype associated with FUS expression. This is in conflict with published data that according to Mirra et al., altering SMN levels does not impact the phenotypes of mouse or Drosophila models of FUS-mediated toxicity. These conflicting results should be discussed. Does MBNL also modulate SMN levels in Drosophila and cell lines? Do moderately higher SMN levels indeed rescue defects in neurite outgrowth and SG formation? In summary, the evidence that MBNL rescues FUS pathology via regulating SMN levels is not convincing at this point. A series of additional experiments would be required to establish a firm link between MBNL and SMN as the relevant mechanism for rescuing FUS toxicity.

4) Controls for iPSC-derived motor neurons are missing:
“Figure 7: MBNL1 downregulation attenuates FUS positive stress granules in iPSC derived motor neurons.”
No controls are shown to demonstrate successful differentiation into Isl1+ or Hb9+ motor neurons.

Additional minor points:

“These observations are further supported by the fact that upregulation of the endogenous wild-type proteins (e.g. FUS or alpha synuclein) due to pathogenic mutations lead to neurodegenerative symptoms in human patients”.

This is misleading, since unlike for a-synuclein, there is no evidence for higher FUS levels in patients causing disease.

“Pathogenic mutations in FUS have been shown to sequester SMN protein, known to cause spinal muscular atrophy, in mammalian neuronal cells and ectopic expression of SMN protects against FUS toxicity in vitro.”
No references are cited to support this statement.

Reviewer #3 (Remarks to the Author):

The authors performed an unbiased genome-wide screen to identify modifiers of FUS toxicity in the fly eye and found that muscleblind modulates FUS toxicity. Reducing muscleblind suppressed FUS-induced eye degeneration, neuromuscular junction defects and impaired motor function without affecting Fus protein levels. Then the authors turn to a mammalian cell line and rat neurons and show that reducing MBNL1 reduces mutant FUS incorporation into stress granules. And that this is due to reduced mislocalization of FUS protein form nucleus to cytoplasm. MBNL KD prevent cell death and dendrite morphology defects of mutant Fus expressing neurons. The authors propose that MBNL rescue may be related to restauration of SMN protein levels. Finally, iPSC-derived motor neurons are used to show that MBNL KD reduced stress granules in mutant FUS lines. Overall the manuscript is well written, and the experiments are easy to follow. This is a well-designed study that expands our knowledge of FUS toxic mechanisms. There are, however, a few points that need to be addressed.

Specific points
- Fig 3d, e and f: need to clarify quantification – two independent experiments counting 20 cells each time?
- Fig 3g: why there is no tubulin signal in total lysates?
- The authors claim that “No significant changes were observed in the N/C ratios of MBNL1 in FUS-expressing cells compared to untransfected controls (Fig. 3i).” but the graph shows that mutant FUS increases MBNL1 N/C ratio…what does that mean?
- Fig 4d: authors need to show MBNL protein levels instead of GFP-shRNA
- Fig 6a: quantification of the aggregates is needed between WT and mutant FUS with control and MBNL shRNA. The results section describing the data for Fig 6 needs to be better explained. The text mentions WT-FUS but the figure only shows non-transfected cells and the text seems to indicate that qPCR was used to quantify aggregates (?).
- Figure 6c: Is there a difference in SMN RNA levels between WT/control and each mutant FUS? Otherwise rescue by MBNL KD cannot be claimed.
- Supplementary Fig 16 is the same as Fig 7a and b.
- Legend of Fig 7 need to be improved, for instance which are the clones SL and LL? What is RFP showing? Were the motor neurons treated with sodium arsenite? The text does not mention that.
- Legend for Fig S15 and S16 are missing.
- Fig S17, S18 and S19 are missing
- What happens to the SMN levels in motor neurons? Does MBNL KD rescues motor neuron degeneration/cell viability? These are important questions that need to be addressed.

We thank the editor for giving us the possibility to submit a revised version of our manuscript. We are very grateful to the reviewers for the thoughtful comments that tremendously helped us in improving the quality of our current manuscript. We strongly believe that we have properly addressed the issues raised by the reviewers. We are providing a point-to-point response to the reviewers' comments below.

Reviewer #1: We thank the reviewer for stating that "this is an intriguing and carefully performed investigation, that unexpectedly yields evidence for MBNL1 as a potentiator of the FUS disease process". We were able to fully address the issues raised by the reviewer by adding additional experimental evidence, references and clarifications.

Comment 1:

There is some uncertainty as to whether MBNL1 RNA is involved in the subcellular localization of FUS protein. Experiments to test the ability of mutant MBNL1 RNA lacking FUS binding sites might clarify the role of FUS – MBNL1 RNA interactions in regulating the stress granule association effect. Another option might be to better understand the nature of the FUS – MBNL1 protein interaction. Is it direct or indirect? Can MBNL1 protein incapable of FUS interaction still promote FUS stress granule association?

Response: To address this issue, we performed immunoprecipitation (IP) and immunofluorescence (IF) assays to determine if MBNL1 and FUS interaction is RNA-dependent. We transfected HEK293T cells with HA-FUS (WT and mutant), performed IP with anti-HA (FUS) with and without RNAse treatment and probed with anti-MBNL1 (endogenous). We observed that RNAse treatment does not alter interaction between FUS and MBNL1 in HEK293T cells suggesting that these two proteins interact in an RNA-independent manner.
To further address this issue, we performed an analogous experiment in HEK293T cells and treated the cells expressing FUS and MBNL1 with RNase. We performed IF to determine if FUS and MBNL1 are recruited into the cytoplasmic aggregates in an RNA-dependent manner. We did not observe any difference in the ability of FUS and MBNL1 to incorporate into the cytoplasmic puncta suggesting that both proteins sequester in an RNA-independent manner. We are including this data as a supplementary figure 5. We are providing data showing that FUS and MBNL1 co-localize into stress granules in a similar manner in the presence and absence of RNase treatment (supplementary figure 5a and b).

We would like to point out that MBNL1 contains a low complexity domain which has not been ever examined in the context of physiological or pathological functions. It is possible that prion-like domain of MBNL1 is involved in regulating FUS-mediated toxicity. We have discussed this possibility in the discussion.

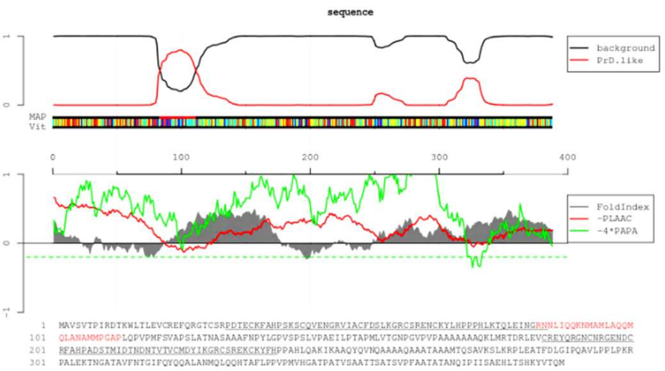

Figure: Presence of the prion-like domain in MBNL1: Using a prion-prediction algorithm, PLAAC which has been used to screen other RNA binding proteins for their prion-like properties, we analyzed the MBNL1 sequence for the presence of prion like domain(s). We used the full-length amino acid sequence of human MBNL1 to determine whether the protein contains functional domains with prion-like properties. The algorithm predicts amino acids 83 to 111 (highlighted in red) of MBNL1 to have prion like sequence

Comment 2:

I do not understand how MBNL1 prevents SMN RNA expression from properly occurring in the context of mutant FUS expression. Is this due to mutant FUS binding to SMN RNA and preventing its processing? Or does this reflect an enhanced directing of SMN RNA to stress granules preventing its transcription? Some additional insight into how MBNL1 affects FUS regulation of SMN RNAs would be helpful.

Response: We fully agree with the reviewer that it is important to understand the functional relationship between MBNL1 and SMN in the context of disease-causing mutations in FUS. As the reviewer suggested, we transiently transfected HEK293T cells with FUS R521C with and with MBNL1 shRNA and examined the endogenous SMN RNA expression by qPCR. We found a significant upregulation in the SMN RNA expression in MBNL1 shRNA group as compared mutant FUS alone (supplementary figure 16a). Importantly, we observed that the SMN RNA levels are also restored in mutant FUS expressing motor neuron like NSC-34 cells upon MBNL1 knockdown (Figure 6c). This data provides a direct link between FUS, MBNL1 and SMN.

It is possible that MBNL1 affects FUS regulation of SMN RNA through a cytoplasmic gain of function (increased stress granule association) and a nuclear loss of function for FUS. Interestingly Sun et al., (2015) https://www.nature.com/articles/ncomms7171) reported mutant FUS induces splicing changes that introduce premature stop codons in target mRNAs and presumably induce nonsense-mediated decay of the mRNA. We are following up these issues by using cell-biological and genetic approaches to determine how MBNL1 is involved in regulating SMN RNA expression and processing under physiological and pathological conditions.

Reviewer #2: We are very thankful to the reviewer for stating that "The identification of MBNL as a modifier of FUS toxicity is certainly a novel and interesting finding". The reviewer also commented that "the manuscript is very thorough and uses model systems from Drosophila to mammalian neuronal and human iPSC-derived motor neurons to make a very strong and convincing case". We have fully addressed all of the concerns raised by the reviewer by providing additional experiments and clarifications in the manuscript.

Comment 1:

The term stress granules (SGs) is used to describe cytoplasmic foci, w/o demonstrating that they are positive for SG markers:

Generally, SGs are identified by the presence of key components, such as G3BP. In this study, cytoplasmic granules are described as SGs, without actually showing the co-localization with SG markers. The exception here is Suppl. Fig. 8, but most figures just show FUS or MBNL-positive puncta.

Response: We agree with the reviewer that it is important to use anti-G3BP antibody for showing the co-localization with SG markers**.** We are including an IF analysis with anti-G3BP antibody (SG marker) to clearly demonstrate that mutant FUS colocalizes with MBNL1 and G3BP in physiological conditions and in stress conditions (now supplementary figure 9). These data provide evidence that FUS and MBL colocalize into SGs, which represents a key and novel observation in support of our conclusions. For this reason, we thank the reviewer for this suggestion.

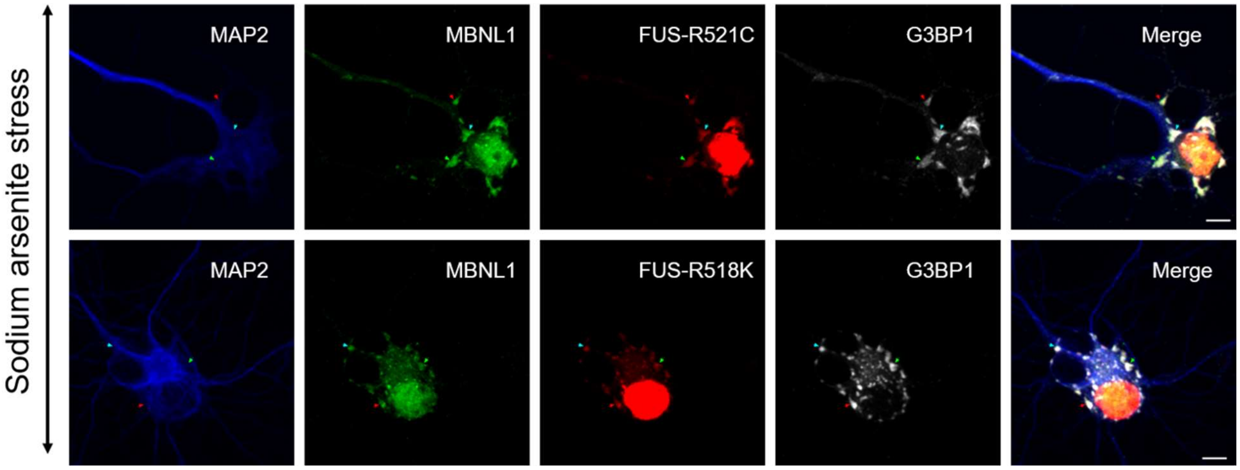

Comment 2:

The question how MBNL levels modulate FUS toxicity and SG formation is not sufficiently explored:

There are obvious possibilities that are not investigated in this study. We now know that FUS localization and aggregation/LLPS depends on its association with transportin 1 and by posttranslational arginine methylation of FUS (see 4 papers in Cell from April 2018). Does MBNL affect this posttranslational modification? It even seems that the lower FUS band is increased by mbl RNAi in Fig. 1h. It would be important to show whether this process that regulates FUS phase separation is affected by MBNL.

Response: As the reviewer suggested, we have investigated the possibilities that MBNL1 affects the posttranslational modification (arginine methylation) of FUS. To address this issue, we expressed FUS (WT and mutant) with GFP-MBNL1 in HEK293 cells and treated the cells with AdOx (a pan-inhibitor of protein arginine methylation), which we have already shown to modify FUS arginine methylation (Scaramuzzino et al., 2013). We performed WB to examine if FUS (lower band) is modulated through arginine modification. We found that neither lower nor upper band intensities are changed with or without MBNL1 RNAi KD in the presence or absence of AdOx (supplementary figure 5). These observations suggest that MBNL1-mediated suppression of FUS toxicity is likely to be independent of arginine methylation modification. We also looked for nuclear and cytoplasmic distribution of FUS in response to AdOx treatment, but we did not observe any difference in the FUS distribution with and without AdOx treatment in any groups further suggesting our observations. We are including this data as a supplementary figure 5. These observations suggest that MBNL1-mediated suppression of FUS toxicity is not dependent of arginine methylation modification.

As the reviewer 1 and 2 suggested, we have explored obvious possibilities (RNA-mediated interaction and arginine methylation) but the MBNL1 does not appear to work through any of these mechanisms suggesting involvement of a previously unexplored molecular pathway(s). Our manuscript would be the first one to report the role of MBNL1 in FUS-mediated neurodegeneration *in vivo* and would spark wide interest in further examining the mechanisms.

Comment 3:

Does MBNL mitigate FUS toxicity by restoring SMN levels? It is unclear what the authors mean with manipulating muscleblind levels to "restore reduced SMN protein levels in axons". The same is true for the heading "SMN protein trapped in axons by pathogenic mutations in FUS is rescued by MBNL1 knockdown". This appears to refer to a study published in HMG in 2013

“ALS-associated mutations in FUS disrupt the axonal distribution and function of SMN”. It is not clear what aggregates are axonal or cytoplasmic and what is rescued. Is SMN reduced in axons? Is SMN trapped in axons? It seems that SMN may be trapped in cytoplasmic inclusions as shown in Fig. 6, but the present study does not quantify SMN levels in axons. NEFL is not an axon-specific marker, unless it is specific for phospho-NF isoforms present only in axons. This is not clear from the methods, and the images do not show axon-specific staining. There is no quantitative WB analysis and the reduction of SMN protein levels appears minor and is not obvious from the ICC staining.

Response: We regret for not clarifying this. We observed that MBNL1 KD mitigates FUS toxicity by upregulating SMN levels in neurons (Figure 6). We used anti-NEFL (NEFL medium chain) antibody that recognizes phosphorylated NEFL specific for axons. This antibody has been well-described in the literature and we are including the references as well. We are providing more details about the antibody in the methodology section. Furthermore, we also validated the specificity of Neurofilament medium chain antibody as axonal marker by co-labeling it with the dendritic marker MAP2 in DIV10 rat primary cortical neurons. As shown in the image, neurofilament medium (NEFL-M) staining (red) does not overlap with the MAP2 (green) thereby confirming the specificity of the NEFL-M antibody. Scale bar =50 µm

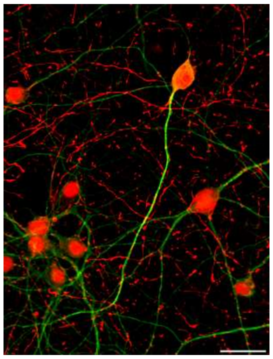

SMN protein is trapped in neuronal cytoplasm expressing mutant FUS mimicking potential loss-of-function of SMN protein. We are providing high quality images showing that SMN protein is trapped in axons supporting our observations (figure 7). Our immunofluorescence images clearly show reduction of SMN protein in axons.

<u>**Comment 4:**</u> The authors also describe that ectopic expression of SMN suppressed the external eye degenerative phenotype associated with FUS expression. This is in conflict with published data that according to Mirra et al., altering SMN levels does not impact the phenotypes of mouse or Drosophila models of FUS-mediated toxicity. These conflicting results should be discussed. Does MBNL also modulate SMN levels in Drosophila and cell lines?

Response: We have discussed findings of Mirra et al., in the context of our findings as suggested (see Discussion). Briefly, there are some key differences between two studies, **(a)** Since there are <u>no reports of the presence of all four mutations, or any of their combinations, in ALS patients</u>, the Mirra’s fly model may represent a particularly severe FUS-mediated toxicity, Mirra et al., used a fly model that contains four dominant FUS mutations (R521G, R522G, R524S and P525L), which are dominant and fully penetrant mutations and each single-point mutation is reported to cause pathogenesis associated with ALS and **(b)** there are <u>no reports of presence of all four mutations or any of their combinations in ALS patients</u> suggesting that compound mutant fly model’s pathophysiological conditions may represent severe FUS-mediated toxicity and might not correlate with pathophysiological conditions of patients with individual FUS mutations. Albeit with the limitations intrinsic to any experimental model, our fly model only expresses one disease-causative mutation, a condition similar to human disease. Because of this fundamental difference, we believe that a comparison between our and the Mirra’s data may not be possible.

Another key difference is that Mirra et al., have examined the effect of overexpression of wild-type human FUS (hFUS) in mice with endogenous (*Smn*$^{+/+}$) and reduced (*Smn*$^{-/+}$) levels of SMN.

In contrast, we have examined the effect of overexpression of SMN and knock down of endogenous smn protein. Our *in vivo* data show that SMN overexpression suppresses FUS pathogenicity and reducing the levels of smn enhances toxicity in our fly model of ALS (Figure 8 c and d). Further, ectopic expression of SMN in cultured primary neurons also protects against FUS toxicity and supports our conclusion (new Figure 8a and b).

We think that the use of different approaches in two studies resulted in distinct data sets that contribute equally important information to the advancement of knowledge in the field of ALS. We are acknowledging these differences in the discussion section of our manuscript.

Regarding the levels of SMN in Drosophila and cell lines, we tried to address this issue using both flies and cell lines. There are no commercially available antibodies for detecting endogenous Smn protein levels in flies. Anti-SMN antibodies commercially available do not cross-react with fly Smn. Due to lack of any reliable antibodies, we were unable to address this issue. We are in a process of generating anti-Smn antibody that specifically recognizes endogenous fly Smn protein and we hope to address this issue in near future. Regarding cell lines, our qPCR and IF data show that the MBNL1 knockdown clearly upregulates SMN protein levels in primary cortical neurons as well as in HEK293T cells (Supplementary Figure 16).

Comment 5:
Do moderately higher SMN levels indeed rescue defects in neurite outgrowth and SG formation? In summary, the evidence that MBNL rescues FUS pathology via regulating SMN levels is not convincing at this point. A series of additional experiments would be required to establish a firm link between MBNL and SMN as the relevant mechanism for rescuing FUS toxicity.

Response: As the reviewer suggested, we expressed SMN in primary cortical neurons expressing FUS (R518K and R521C) and measured neurite outgrowth. We found that upregulation of SMN levels strongly suppressed the neurite outgrowth defects caused by mutant FUS expression (Figure 8), suggesting that ectopic expression of SMN protects against FUS toxicity in mammalian cortical neurons expressing mutant FUS. Our data further support the fly data as well as primary cortical neuron data showing the upregulation of SMN protein modulates FUS toxicity. We are including these new data in Figure 8. Furthermore, we went on to determine the impact of higher levels of SMN on mutant FUS positive SGs. We observed that upregulation of SMN levels significantly reduces G3BP1 and FUS-positive SGs in primary cortical neurons (Supplementary figure 16b and c).

Comment 6: Controls for iPSC-derived motor neurons are missing:
“Figure 7: MBNL1 downregulation attenuates FUS positive stress granules in iPSC derived motor neurons.” No controls are shown to demonstrate successful differentiation into Isl1+ or Hb9+ motor neurons.

Response: We are including the data demonstrating successful differentiation of iPSC motor neurons into Hb9+ motor neurons. We are providing this data in our revised manuscript (Please see supplementary figure 18).

Comment 7:
Additional minor points: “These observations are further supported by the fact that upregulation of the endogenous wild-type proteins (e.g. FUS or alpha synuclein) due to pathogenic mutations lead to neurodegenerative symptoms in human patients”. This is misleading, since unlike for a-synuclein, there is no evidence for higher FUS levels in patients causing disease.

Response: We thank the reviewer for making this comment. Sabatelli *et al, (*2013) reported a novel variant in 3' untranslated region of FUS that **upregulated the endogenous levels of FUS protein** in a patient family with classical ALS symptoms. We are including this reference in the manuscript.

Comment 8:
"Pathogenic mutations in FUS have been shown to sequester SMN protein, known to cause spinal muscular atrophy, in mammalian neuronal cells and ectopic expression of SMN protects against FUS toxicity in vitro." No references are cited to support this statement.

Response: We are including a reference to support our statement (please see reference 74 Groen et al., 2013 HMG)

Reviewer #3: Overall the manuscript is well written, and the experiments are easy to follow. This is a well-designed study that expands our knowledge of FUS toxic mechanisms. There are, however, a few points that need to be addressed.

Comment 1:
Fig 3d, e and f: need to clarify quantification – two independent experiments counting 20 cells each time?

Response: We have quantified 60-120 cells for doing the quantification (Figure 3). We are including more details in the methodology section.

Comment 2:
Fig 3g: why there is no tubulin signal in total lysates?

Response: We regret for this mistake. We are including blots showing tubulin signal in total lysates (figure 3g).

Comment 3:
The authors claim that "No significant changes were observed in the N/C ratios of MBNL1 in FUS-expressing cells compared to untransfected controls (Fig. 3i)." but the graph shows that mutant FUS increases MBNL1 N/C ratio…what does that mean?

Response: We regret for making this typo. The reviewer is correct. We have revised our statement in the text. These observations suggest that mutant FUS expression partially increases the N/C ratio of MBNL1 as compared to untransfected controls. This is consistent with our FUS patient data where we observed a slight but significant increase in MBNL1 RNA levels specifically in FUS-R518K (Supplementary fig. 6a). We do not know why only FUS-R518K upregulates MBNL1 protein levels and we will follow up on this question in our ongoing studies.

Comment 4:
Fig 4d: authors need to show MBNL protein levels instead of GFP-shRNA

Response: We agree with the reviewer. We have done extensive characterization of our GFP tagged shRNA constructs. We are including a WB and qPCR data showing that MBNL1 GFP-shRNA construct significantly reduced the endogenous protein and RNA levels, respectively (Figure 4a). We have also examined MBNL1 protein levels and providing the data in

supplementary figure 13a.

Comment 5:
Fig 6a: quantification of the aggregates is needed between WT and mutant FUS with control and MBNL shRNA. The results section describing the data for Fig 6 needs to be better explained. The text mentions WT-FUS but the figure only shows non-transfected cells and the text seems to indicate that qPCR was used to quantify aggregates (?).

Response: We agree and regret for not being clear in the text. We quantified the percentage of neurons with SMN aggregates in mutant FUS with scramble shRNA and MBNL1 shRNA. We have clarified this in the manuscript as well as legends. Regarding qPCR, we only quantified the smn RNA expression as indicated in the figure and text.

Comment 6:
Figure 6c: Is there a difference in SMN RNA levels between WT/control and each mutant FUS? Otherwise rescue by MBNL KD cannot be claimed.

Response: Excellent point! Yes, we did observe a clear difference between WT and mutant FUS expressing groups (figure 6d).

Comment 7:
Supplementary Fig 16 is the same as Fig 7a and b.

Response: The supplementary figure 16 (now sup figure 17) and figure 7a and b are not the same. The figure 7a and b were generated using MBNL1 shRNA (construct 1) and the supplementary figure 17 was generated using a different MBNL1 shRNA (construct 2). Both MBNL1 shRNA constructs were validated prior to the use (figure 7c and d) and they show knockdown of endogenous MBNL1 in iPSC motor neurons. The left side labeling in the IF image indicates the shRNA constructs we used in these figures.

Comment 8:
Legend of Fig 7 need to be improved, for instance which are the clones SL and LL? What is RFP showing? Were the motor neurons treated with sodium arsenite? The text does not mention that.

Response: We have updated the figure 7 legends and included more details about the clones SL and LL. Briefly, the clones SL stands for short linker and LL stands for long linker. These iPSC lines are well-characterized and published (Marrone et al., 2018; Reinhardt et al., 2019). We are providing a figure from our recent collaborative manuscript showing the LL and SL linkers (Marrone et al., 2018) and citing this paper as well. These iPSC-derived motor neurons were treated with 500 μM sodium arsenite for one hour for inducing stress granules.

[redacted]

Comment 9:
Legend for Fig S15 and S16 are missing.

Response: We are including the legends for supplementary figure 15 and 16.

Comment 10:
Fig S17, S18 and S19 are missing

Response: We have updated the supplementary figures and we are including new supplementary figures.

Comment 11:
What happens to the SMN levels in motor neurons? Does MBNL KD rescues motor neuron degeneration/cell viability? These are important questions that need to be addressed.

Response: The reviewer raised a very valid point. We examined the levels of SMN protein in primary motor neurons expressing mutant FUS and observed that SMN aggregation with FUS and loss of SMN in axons similar to cortical neurons (Supplementary figure 19).

As the reviewer suggested, we examined additional phenotypes like neuronal degeneration and cell viability in our primary cortical neuron models as well as fly models. We found that MBNL1 KD strongly rescues neuronal death as well as morphological defects in primary neurons as well as in *Drosophila* (figure 1, 2 and 7).

We believe that we are providing compelling and strong evidences to support our observations using multiple model systems. We hope that the reviewers will appreciate our efforts and hard work in addressing their concerns. We are including one new main figure (figure 8) and 4 new supplementary figures in our revised manuscript.

Reviewers' comments:

Reviewer #1 (Remarks to the Author):

The revised ms is much improved, and the authors have adequately addressed my concerns.

Reviewer #2 (Remarks to the Author):

Generally, the authors have been responsive and as the manuscript is now much improved. The authors have fully addressed most of the concerns raised by this reviewer by providing additional experiments and clarifications in the manuscript.

However, there are remaining important concerns with original comments #3 and 5 that have not been adequately addressed yet as outlined below.

Comment 3: "Does MBNL mitigate FUS toxicity by restoring SMN levels?"

The current title "Muscleblind mitigates FUS toxicity by modulating stress granule dynamics and restoring SMN levels", does not reflect what the paper actually shows. This title suggests that FUS toxicity, much like SMA, is characterized by low SMN protein levels, and that Muscleblind (expression?) restores normal SMN levels. Instead, the authors show mislocalization/sequestration of SMN into cytoplasmic foci with mutant FUS, leading to reduced axonal SMN protein levels, and that MNBL1 knock down restores SMN axonal localization and also increases SMN mRNA levels. A more appropriate title would be "Muscleblind knockdown mitigates FUS toxicity by modulating stress granule dynamics and restoring SMN localization" or "Muscleblind acts as a modifier (or enhancer) of FUS toxicity by modulating stress granule dynamics and SMN localization".

These two previous reviewer's comments have not been fully addressed:
1) "It seems that SMN may be trapped in cytoplasmic inclusions as shown in Fig. 6, but the present study does not quantify SMN levels in axons."
2) "There is no quantitative WB analysis and the reduction of SMN protein levels appears minor and is not obvious from the ICC staining."

As for 1), this reviewer is still confused by the contradictory response "We are providing high quality images showing that SMN protein is trapped in axons supporting our observations (figure 7). Our immunofluorescence images clearly show reduction of SMN protein in axons". It appears that SMN is trapped in cytoplasmic granules, and therefore reduced in axons. Figure 6b now appears to show quantification of axonal SMN protein levels, as suggested by this reviewer, but this is not made as clear as it should be. In the title and most of the manuscript, "reduced SMN protein" and "loss of SMN" are used, when "mislocalization/sequestration of SMN" and "reduced axonal SMN levels" should be used. I am not being pedantic here, but most of the statements throughout the title, abstract, and main text referring to SMN levels/localization are just misleading or plainly wrong, and should be corrected. The data do not show "reduced SMN levels" and "restored SMN levels" as stated.

As a minor point, % of neurons with SMN aggregates should rather say "puncta" or "foci", unless evidence for solubility and true aggregation is provided.

As another minor point, the figure legend states "Quantification of fluorescence intensity of SMN1 staining along NEFL positive axons confirms that depleting endogenous MBNL1 significantly rescues loss of SMN1 in mutant FUS neurons." There is no loss of SMN1 protein, but mislocalization/sequestration of SMN protein. Loss of the SMN1 gene (!) causes reduced SMN protein levels and SMA.

As another point, there is no SMN1 protein. In humans, both SMN1 and SMN2 gene copies encode SMN protein. Mice have only one Smn gene encoding SMN protein.

As for 2), I would suggest again to measure SMN protein (!) levels in 293T cells after MBNL1 knock down. It is still confusing that the authors claim 2 seemingly unrelated mechanisms: 1) SMN is trapped into aggregates with FUS and released by knock down of MBNL1 2) SMN mRNA levels are increased upon MBNL1 knockdown. The authors state that "Knocking down MBNL1 upregulated SMN RNA levels in mutant FUS expressing cells as compared to control cells (Supplementary Fig. 16a). These findings suggest that MBNL1 knock down is sufficient to restore the levels of SMN otherwise trapped in the cytoplasmic aggregates due to FUS-ALS mutations." It is unclear how these findings are related. Is the rescue due to increased SMN mRNA levels? There is no strong evidence that SMN mRNA levels are reduced in the FUS models. Are these increased levels then also sequestered by mutant FUS? To investigate this, increased SMN protein (!) levels should be shown, since SMN mRNA levels alone are not meaningful? This can be done in HEK293T cells.

As a minor point, the authors also overstate that "Human genetic studies supported by experimental models suggest the involvement of SMN in ALS pathogenesis (83)." This statement goes beyond what ref. 83 discusses, namely the hypothesis of a common critical pathways linked to RNA-transcriptome homeostasis underlying motor neuron degeneration in both these diseases. There is conflicting evidence whether SMN1 and SMN2 copy number variations are linked to ALS.

Comment 5: "Do moderately higher SMN levels indeed rescue defects in neurite outgrowth and SG formation? In summary, the evidence that MBNL rescues FUS pathology via regulating SMN levels is not convincing at this point."

This comment has not been fully addressed. The authors show that SMN overexpression is neuritogenic in motor neurons expressing mutant FUS. Does mutant FUS overexpression cause axon outgrowth defects as implied but not shown? Is this rescued by SMN overexpression, as suggested? Only 4 conditions are shown: Overexpression of FUS R521C and R518K, and co-overexpression of FUS R521C and R518K with SMN. There are no controls shown to indicate a specific axon outgrowth defect caused by mutant FUS overexpression. Does MNBL1 knock down rescue this axonal phenotype too? Supplementary figure 16 is entitled "Knockdown of endogenous muscleblind upregulates SMN RNA levels and reduces FUS positive SGs:" but appears to show instead that SMN overexpression (!) reduces FUS-positive SGs.
Minor comments: Several figures lack scale bars (e.g. Fig. 7, and Suppl. Fig 17 and 18).

In summary, I would suggest the following changes:
1) Change the title to match the actual findings.
2) Edit the text to accurately describe mislocalization/sequestration of SMN protein instead of "loss" or "reduced protein levels" that are not shown. It is also not clear that SMN mRNA levels are restored, since there is no evidence that SMN mRNA or protein levels are significantly reduced in the FUS models as compared to controls.
3) Show if MNBL1 knock down affects SMN protein levels.
4) Demonstrate if mutant FUS overexpression causes axon outgrowth defects as compared to controls, and if this phenotype is rescued by SMN over expression and MNBL1 knock down.

Reviewer #3 (Remarks to the Author):

The authors have satisfactorily addressed and responded to most of the reviewer comments. I still have a few comments/suggestions:
- please provide in supplementary figure the WB images for Fig 3h

- Fig 6b: quantification of fluorescence intensity is not the best way to measure protein levels, and on top of that very few neurons were analyzed (legend says minimum 5-9 neurons)
- Fig 6d: please provide statistics for “scramble shRNA vs Fus-R521C+scramble shRNA” and “scramble shRNA vs Fus-R518k+scramble shRNA” - ANOVA would be a better method to analyze all the groups
- Fig 7 : according to Sup. Fig 18a, the protocol generates a small amount of motor neurons (Hb9+ cells) so if the authors want to claim that the effects happen in motor neurons they need to analyze Hb9+ cells only. The meaning of 2, 16 and 17 for the P525L lines is missing in the legend.

We thank the editor for giving us an opportunity to further revise our manuscript. We are very grateful to the reviewers for the thoughtful comments that tremendously helped us in improving the quality of our current manuscript. We strongly believe that we have properly addressed the issues raised by the reviewers. We are providing a point-to-point response to the reviewers' comments below.

Reviewer #1: This reviewer was satisfied with our first revision. We thank him/her for the positive comments and for considering our manuscript suitable for publication.

Reviewer #2: Although after the first round of reviews, the reviewer stated that "*the authors have been responsive, and the manuscript is now much improved*", and "*The authors have fully addressed most of the concerns raised by this reviewer by providing additional experiments and clarifications in the manuscript*", he/she made very valid points on how to further improve our manuscript. We thank the reviewer praising our work as well as for pointing out the remaining concerns that, we believe, that we have fully addressed in this third round of revisions.

Comment 1: The current title "Muscleblind mitigates FUS toxicity by modulating stress granule dynamics and restoring SMN levels", does not reflect what the paper actually shows. A more appropriate title would be "Muscleblind knockdown mitigates FUS toxicity by modulating stress granule dynamics and restoring SMN localization" or "Muscleblind acts as a modifier (or enhancer) of FUS toxicity by modulating stress granule dynamics and SMN localization".

Response:
We changed the title of our manuscript according to the following reviewer's suggestion to "Muscleblind acts as a modifier of FUS toxicity by modulating stress granule dynamics and SMN localization"

Comment 2: As a minor point, % of neurons with SMN aggregates should rather say "puncta" or "foci", unless evidence for solubility and true aggregation is provided.

Response:
We agree with the reviewer. We have gone through the manuscript and changed the term SMN aggregates to foci or puncta throughout the manuscript text, figures as well as legends.

Comment 3: As another minor point, the figure legend states "Quantification of fluorescence intensity of SMN1 staining along NEFL positive axons confirms that depleting endogenous MBNL1 significantly rescues loss of SMN1 in mutant FUS neurons." There is no loss of SMN1 protein, but mislocalization/sequestration of SMN protein. Loss of the SMN1 gene (!) causes reduced SMN protein levels and SMA.

Response:
We have modified the text as the reviewer suggested and used the term SMN protein mislocalization throughout the text. Please see our response to comment #5 below as well.

Comment 4: As another point, there is no SMN1 protein. In humans, both SMN1 and SMN2 gene copies encode SMN protein. Mice have only one Smn gene encoding SMN protein.

Response:
We agree, and we have changed the SMN1 to SMN throughout the manuscript.

Comment 5: These two previous reviewer's comments have not been fully addressed:

1) "It seems that SMN may be trapped in cytoplasmic inclusions as shown in Fig. 6, but the present study does not quantify SMN levels in axons."
2) "There is no quantitative WB analysis and the reduction of SMN protein levels appears minor and is not obvious from the ICC staining."

As for 2), I would suggest again to measure SMN protein (!) levels in 293T cells after MBNL1 knock down. It is still confusing that the authors claim 2 seemingly unrelated mechanisms: 1) SMN is trapped into aggregates with FUS and released by knock down of MBNL1 2) SMN mRNA levels are increased upon MBNL1 knockdown. The authors state that "Knocking down MBNL1 upregulated SMN RNA levels in mutant FUS expressing cells as compared to control cells (Supplementary Fig. 16a, now supplementary figure 17a). These findings suggest that MBNL1 knock down is sufficient to restore the levels of SMN otherwise trapped in the cytoplasmic aggregates due to FUS-ALS mutations." It is unclear how these findings are related. Is the rescue due to increased SMN mRNA levels? There is no strong evidence that SMN mRNA levels are reduced in the FUS models. Are these increased levels then also sequestered by mutant FUS? To investigate this, increased SMN protein (!) levels should be shown, since SMN mRNA levels alone are not meaningful? This can be done in HEK293T cells.

<u>Response</u>: The reviewer is absolutely right. We agree. To address the comments about SMN protein and RNA levels in FUS, we examined the levels of SMN protein in motor neuron like cells NSC34 cells by Western blotting. We found that expression of FUS R518K and R521C significantly reduced the levels of endogenous SMN protein. We found that shRNA-mediated knockdown of endogenous MBNL1 significantly upregulated the SMN protein levels <u>(Supplementary figure 17a-b).</u> In our IF experiments, we found that SMN is entrapped in FUS positive puncta in the cytoplasm (Figure 6 and Supplementary Figure 17d-e) which is rescued by MBNL1 KD. Based on this observation, we believed that this depicts a potential loss of SMN function.
In summary, we agree that the changes in RNA levels are very modest and we believe that MBNL1-mediated changes in SMN levels occur at the protein level. We have modified our statement in the manuscript accordingly.

Comment 6: As a minor point, the authors also overstate that "Human genetic studies supported by experimental models suggest the involvement of SMN in ALS pathogenesis (83)." This statement goes beyond what ref. 83 discusses, namely the hypothesis of a common critical pathways linked to RNA-transcriptome homeostasis underlying motor neuron degeneration in both these diseases. There is conflicting evidence whether SMN1 and SMN2 copy number variations are linked to ALS.

<u>Response:</u>
We agree. As the reviewer suggested, we have revised our statement regarding the role of SMA in ALS pathogenesis and we are including two additional references on conflicting evidences as well making our statement unbiased.

Comment 7: "Do moderately higher SMN levels indeed rescue defects in neurite outgrowth and SG formation? In summary, the evidence that MBNL rescues FUS pathology via regulating SMN levels is not convincing at this point."

This comment has not been fully addressed. The authors show that SMN overexpression is neuritogenic in motor neurons expressing mutant FUS. Does mutant FUS overexpression cause axon outgrowth defects as implied but not shown? Is this rescued by SMN overexpression, as suggested? Only 4 conditions are shown: Overexpression of FUS R521C and R518K, and co-

overexpression of FUS R521C and R518K with SMN. There are no controls shown to indicate a specific axon outgrowth defect caused by mutant FUS overexpression. Does MNBL1 knock down rescue this axonal phenotype too? Supplementary figure 17 is entitled "Knockdown of endogenous muscleblind upregulates SMN RNA levels and reduces FUS positive SGs:" but appears to show instead that SMN overexpression (!) reduces FUS-positive SGs.

Response:
To address if mutant FUS expression causes axon outgrowth defects and if this is rescued by SMN overexpression we have now carried out axon outgrowth measurement experiments suggested by the reviewer using primary motor neurons. For this assay we included the relevant controls (GFP and GFP tagged SMN) and labeled axons using Tau. We are including this data in Fig 8 e-f in the revised manuscript. Quantification of axon outgrowth data clearly shows SMN overexpression does rescue axon outgrowth defects.

We thank the reviewer for pointing out the confusion in the supplementary figure 16 title (now supplementary figure 17). We have now rephrased the title "Knockdown of endogenous muscleblind upregulates SMN protein and RNA levels and overexpression of SMN in mutant FUS neurons reduces FUS positive SGs" in the revised manuscript.

Minor comments: Several figures lack scale bars (e.g. Fig. 7, and Suppl. Fig 17 and 18).

Response:
We have added the scale bars on every figure in the manuscript.

Comment 8: In summary, I would suggest the following changes:
1) Change the title to match the actual findings.
2) Edit the text to accurately describe mislocalization/sequestration of SMN protein instead of "loss" or "reduced protein levels" that are not shown. It is also not clear that SMN mRNA levels are restored, since there is no evidence that SMN mRNA or protein levels are significantly reduced in the FUS models as compared to controls.
3) Show if MNBL1 knock down affects SMN protein levels.
4) Demonstrate if mutant FUS overexpression causes axon outgrowth defects as compared to controls, and if this phenotype is rescued by SMN over expression and MNBL1 knock down.

Response:
As the reviewer suggested, we have 1) modified the title of our manuscript as suggested by the reviewer 2; 2) replaced the terms "loss" or "reduced protein levels" throughout the text; 3) we are including WB along with quantification showing that MBNL1 KD affect SMN protein levels (Supplementary figure 17a-b) and RNA levels in mouse neuronal cell line NSC34 and HEK293T (Figure 6d and supplementary figure 17c); and 4) providing evidence that mutant FUS overexpression causes axon outgrowth which is rescued by SMN in motor neurons (Figure 8). Similarly, expression of mutant FUS leads to dendritic branching defects and neuronal death which is rescued by MBNL1 KD in primary cortical neurons (Figure 5).

We hope that the reviewer 2 will recognize our sincere efforts to address the concerns in our 2nd and 3rd versions of revised manuscript and will make a favorable decision. We believe that our findings would spark a lot of interest in understanding the basic biology of muscleblind, SMN and FUS proteins.

Reviewer #3: We thank the reviewer for stating “The authors have satisfactorily addressed and responded to most of the reviewer comments”.

Comment 1: Please provide in supplementary figure the WB images for Fig 3h

Response:
We are including representative WB images for figure 3h in the supplementary figure 11d.

Comment 2: Fig 6b: quantification of fluorescence intensity is not the best way to measure protein levels, and on top of that very few neurons were analyzed (legend says minimum 5-9 neurons)

Response:
We agree with the reviewer. We have done Western blotting to support our fluorescence data and we are including the data as supplementary figure 17a-b.

Comment 3: Fig 6d: please provide statistics for “scramble shRNA vs Fus-R521C+scramble shRNA” and “scramble shRNA vs Fus-R518k+scramble shRNA” - ANOVA would be a better method to analyze all the groups

Response:
We are providing statistics for “scramble shRNA vs Fus-R521C+scramble shRNA” and “scramble shRNA vs Fus-R518k+scramble shRNA” as suggested by the reviewer. We are including this info in the figure legends.

Comment 4: Fig 7: according to Sup. Fig 18a (now Sup. Fig 19), the protocol generates a small amount of motor neurons (Hb9+ cells) so if the authors want to claim that the effects happen in motor neurons they need to analyze Hb9+ cells only. The meaning of 2, 16 and 17 for the P525L lines is missing in the legend.

Response:
We thank the reviewer for their valuable feedback. We used three different isogenic clones of FUS P525L iPSC line and these isogenic clones are labeled as 2, 16 and 17. We are including the meaning 2, 16 and 17 for the P525L line in the legend. These lines are well characterized and published by our group (Marrone et al., 2018). We are including this reference.

We agree that we do not have 100% pure motor neuron population. We have improved the manuscript text to state and clarify that all experiments analyzed iPSC-derived neuronal culture, which were mixed cultures containing about 50% motor neurons. We have included immunostaining data showing that almost all cells in our cultures were MAP2 positive, which marks terminally differentiated neurons, and 50% of the cells expressed HB9 and Islet1, which mark motor neurons. The protocol is an improved variation of one that we recently previously reported [1]. To the best of our knowledge, currently there is no iPSC motor neuron differentiation protocol available to reliably obtain 100% pure motor neuron population.

1 Marrone, L. *et al.* Isogenic FUS-eGFP iPSC Reporter Lines Enable Quantification of FUS Stress Granule Pathology that Is Rescued by Drugs Inducing Autophagy. *Stem Cell Reports* **10**, 375-389 (2018).

REVIEWERS' COMMENTS:

Reviewer #2 (Remarks to the Author):

The authors have now addressed most of the critical concerns in the 2nd and 3rd versions of the revised manuscript.

Remaining issues are listed below:

"By immunofluorescence SMN was sequestered into puncta containing mutant FUS in neuronal cytoplasm. Interestingly, we observed that knocking down MBNL1 rescued SMN localization by reducing the number of cytoplasmic puncta where SMN becomes trapped (Fig. 6a and c, Supplementary Fig. 16)."

-> More accurate: As shown by immunofluorescence, SMN was sequestered into puncta containing mutant FUS in neuronal cytoplasm. Interestingly, we observed that knocking down MBNL1 rescued SMN localization by reducing the number of FUS-positive cytoplasmic puncta containing SMN (Fig. 6a and c, Supplementary Fig. 16)

Figure legend for Fig. 6 (c): Quantification of percentage of neurons with SMN aggregates in the cytoplasm. A minimum of 5-10 cells were analyzed from each group from 3 independent experiments. Percentage of neurons with cytoplasmic SMN foci is significantly reduced in mutant FUS neurons (R521C and R518K) co-expressing MBNL1 shRNA compared to cells expressing scramble shRNA.

-> More accurate: Quantification of percentage of neurons with cytoplasmic foci containing both FUS and SMN. A minimum of 5-10 cells were analyzed from each group from 3 independent experiments. Percentage of neurons with FUS and SMN-positive cytoplasmic foci is significantly reduced in mutant FUS neurons (R521C and R518K) co-expressing MBNL1 shRNA compared to cells expressing scramble shRNA.

Figure legend for Supplementary figure 17: Knockdown of endogenous muscleblind upregulates SMN protein and RNA levels and reduces FUS positive SGs:

-> More accurate: Knockdown of endogenous muscleblind upregulates SMN protein and RNA levels and SMN overexpression reduces incorporation of mutant FUS into cytoplasmic stress granules.

"For co-expression experiments 500 ng each of mCherry tagged FUS-R521C or FUS-R518K and 500 ng tagged SMN were used per well."
How much DNA was used for single FUS transfections in Suppl. Fig. 17 d)? Ideally, 500 ng each of mCherry tagged FUS and 500ng of GFP ctrl. plasmid should have been used. Changing amounts or ratios of DNA will impact FUS expression levels in transfected cells.

We thank the editor for accepting to publish our manuscript in principle. We are very grateful to the reviewers for the thoughtful comments that tremendously helped us in improving the quality of our current manuscript. We strongly believe that we have properly addressed the issues raised by the reviewer 2. We are providing a point-to-point response to the reviewer 2 comments below.

Reviewer #1 and 3: We thank both reviewers for the positive comments and recommending our manuscript for publication.

Reviewer 2:

Comment 1: "By immunofluorescence SMN was sequestered into puncta containing mutant FUS in neuronal cytoplasm. Interestingly, we observed that knocking down MBNL1 rescued SMN localization by reducing the number of cytoplasmic puncta where SMN becomes trapped (Fig. 6a and c, Supplementary Fig. 16)."

-> More accurate: As shown by immunofluorescence, SMN was sequestered into puncta containing mutant FUS in neuronal cytoplasm. Interestingly, we observed that knocking down MBNL1 rescued SMN localization by reducing the number of FUS-positive cytoplasmic puncta containing SMN (Fig. 6a and c, Supplementary Fig. 16)

Response: As the reviewer suggested, we modified the figure legend and used the exact wordings.

Comment 2: Figure legend for Fig. 6 (c): Quantification of percentage of neurons with SMN aggregates in the cytoplasm. A minimum of 5-10 cells were analyzed from each group from 3 independent experiments. Percentage of neurons with cytoplasmic SMN foci is significantly reduced in mutant FUS neurons (R521C and R518K) co-expressing MBNL1 shRNA compared to cells expressing scramble shRNA.

-> More accurate: Quantification of percentage of neurons with cytoplasmic foci containing both FUS and SMN. A minimum of 5-10 cells were analyzed from each group from 3 independent experiments. Percentage of neurons with FUS and SMN-positive cytoplasmic foci is significantly reduced in mutant FUS neurons (R521C and R518K) co-expressing MBNL1 shRNA compared to cells expressing scramble shRNA.

Response: As the reviewer suggested, we modified the figure legend and used the exact wordings.

Comment 3: Figure legend for Supplementary figure 17: Knockdown of endogenous muscleblind upregulates SMN protein and RNA levels and reduces FUS positive SGs:

-> More accurate: Knockdown of endogenous muscleblind upregulates SMN protein and RNA levels and SMN overexpression reduces incorporation of mutant FUS into cytoplasmic stress granules.

Response: As the reviewer suggested, we modified the figure legend and used the exact wordings.

Comment 4: "For co-expression experiments 500 ng each of mCherry tagged FUS-R521C or FUS-R518K and 500 ng tagged SMN were used per well."
How much DNA was used for single FUS transfections in Suppl. Fig. 17 d)? Ideally, 500 ng each of mCherry tagged FUS and 500ng of GFP ctrl. plasmid should have been used. Changing amounts or ratios of DNA will impact FUS expression levels in transfected cells.

Response: We have explained this more clearly in the methods section now. For all the experiments we kept the amount of FUS DNA consistent (i.e. 500 ng) along with an empty plasmid or SMN GFP. Therefore, the total DNA concentration was always 1 microgram and the ratio of both the plasmids was kept 1:1 to rule out any impact on FUS expression
